# Robust Inference from Conditional Logistic Regression Applied to Movement and Habitat Selection Analysis

**DOI:** 10.1371/journal.pone.0169779

**Published:** 2017-01-12

**Authors:** Marie-Caroline Prima, Thierry Duchesne, Daniel Fortin

**Affiliations:** 1 Département de Biologie, Université Laval, Québec, Québec, Canada; 2 Département de mathématiques et de statistique, Université Laval, Québec, Québec, Canada; University of Waikato, NEW ZEALAND

## Abstract

Conditional logistic regression (CLR) is widely used to analyze habitat selection and movement of animals when resource availability changes over space and time. Observations used for these analyses are typically autocorrelated, which biases model-based variance estimation of CLR parameters. This bias can be corrected using generalized estimating equations (GEE), an approach that requires partitioning the data into independent clusters. Here we establish the link between clustering rules in GEE and their effectiveness to remove statistical biases in variance estimation of CLR parameters.

The current lack of guidelines is such that broad variation in clustering rules can be found among studies (e.g., 14–450 clusters) with unknown consequences on the robustness of statistical inference. We simulated datasets reflecting conditions typical of field studies. Longitudinal data were generated based on several parameters of habitat selection with varying strength of autocorrelation and some individuals having more observations than others. We then evaluated how changing the number of clusters impacted the effectiveness of variance estimators. Simulations revealed that 30 clusters were sufficient to get unbiased and relatively precise estimates of variance of parameter estimates. The use of destructive sampling to increase the number of independent clusters was successful at removing statistical bias, but only when observations were temporally autocorrelated and the strength of inter-individual heterogeneity was weak. GEE also provided robust estimates of variance for different magnitudes of unbalanced datasets. Our simulations demonstrate that GEE should be estimated by assigning each individual to a cluster when at least 30 animals are followed, or by using destructive sampling for studies with fewer individuals having intermediate level of behavioural plasticity in selection and temporally autocorrelated observations. The simulations provide valuable information to build reliable habitat selection and movement models that allow for robustness of statistical inference without removing excessive amounts of ecological information.

## Introduction

Spatio-temporal changes in the availability of resources to consumers can have profound effects on the patterns of animal distributional dynamics [1–3]. Arthur et al. [4] developed a design to account for such frequent spatio-temporal changes in the availability of habitat features by defining availability separately for each observation of habitat use. Every observed location (case) is paired with several potential locations (controls) that are locally available to the individual at a given time. The resulting dataset is comprised of a binary response variable (1 = case, 0 = control), where each response is associated with habitat covariates, and each case and its associated controls are pooled within the same stratum. This matched case–control design considers that individuals may not have access to their whole home range during the relocation interval [5]. Early habitat selection studies that considered spatio-temporal changes in availability did not take advantage of statistics developed for case-control designs [4,6]. Compton et al. [5] then outlined advantages of using paired or conditional logistic regression (CLR) when resource availability changes over space. Conditional logistic regression compares use with availability at the same place and time, and is now increasingly used in habitat selection studies [7]. Even animal movement is becoming analyzed based on CLR [8–10]. By contrasting characteristics of observed and random steps with CLR, step selection function (*sensu* Fortin et al. [11]) allows for inference on animal movement similar to biased correlated random walks [12].

The enhanced performance of Global Positioning Systems in recent years has increased the relocation frequency of individuals in habitat selection and movement studies [13], such that individuals are commonly relocated 24 or more times a day [9,14,15]. To provide robust inference (i.e., robust estimates of the variance of the regression coefficients), conditional logistic regression has to account for temporal autocorrelation that is inherent to such rich longitudinal datasets. Falsely assuming independence among observations may lead to over- or under-estimation of the variance associated with estimates of the regression coefficient [16]. It has become common practice to use generalized estimating equations (GEE) to cope with temporal autocorrelation in longitudinal data [9,10,17,18]. GEE have also to account for a second source of non-independence in successive observations [3,19]. Indeed, individuals may react differently to various habitat features due to differences in their experience, social status, age, sex, and physical condition [18,20–22]. To fit CLR models with GEE, strata (i.e., groups of observed and random locations) must be split into independent clusters, which implies that observations in one cluster must be statistically independent from those in other clusters. The effectiveness of GEE then depends upon the rules that are used when partitioning the data into independent clusters.

Even though the scheme for partitioning the data is simple, no common practice has been noted in the literature for implementing it. The number of independent clusters that are used varies from one study to another, ranging as broadly as 14 to 450 clusters [9,23]. Some studies create a single cluster per individual [24], whereas others have split the strata of each animal into several clusters [25]. This broad diversity in GEE designs could be explained by the current lack of clear guidelines. In fact, the consequences of such broad variation in clustering rules that can be exerted on the robustness of statistical inferences remains poorly documented, despite the increasing use of GEE in habitat selection and movement studies [26].

Our aim was to determine how clustering rules in GEE affect their ability to decrease the statistical bias in variance estimation due to correlation in longitudinal data. More specifically, 1) we tested for the effect of the number of clusters on robust estimates of variance according to the strength and source of correlation in the response variable (i.e., inter-individual heterogeneity and temporal autocorrelation), 2) we determined when destructive sampling, which consists of removing blocks of strata so that strata in different clusters are temporally uncorrelated for a given individual [11], improves the robust estimate of variance, and 3) we tested the effect of having an unbalanced dataset on robust estimates of variance.

## Materials and Methods

When a GEE is used to estimate a conditional logistic regression, naive and robust estimates of the variance of the regression coefficients are typically computed. To test for the effect of clustering rules on the robust and naive estimates of variance, we simulated datasets that consisted of independent clusters but dependent strata (Dataset simulation) for which we considered different clustering scenarios (Data processing). We then estimated the parameters of the CLR model for each simulated sample, obtained their naive and robust variance estimates and compared the averages of these variance estimates over all samples to the true variance of the CLR coefficient estimates (Statistical analysis).

### Notation and model

Consider *K* independent clusters: one cluster represents successive data from one individual, with all individuals (i.e., clusters) being independent of one another. Consider ${\tilde{Y}}_{1}^{\left(k\right)},\ldots ,{\tilde{Y}}_{T}^{\left(k\right)}$, *T* Bernoulli random variables from which we generate *S* Bernoulli random vectors $Y_{1}^{\left(k\right)},\ldots ,Y_{S}^{\left(k\right)}$, which represent successive data from cluster *k*, *k ϵ* {1,…,*K*}. Each random vector $Y_{j}^{\left(k\right)}$, *j ϵ* {1,…,*S*}, is a vector of {0,1} observations $Y_{j}^{\left(k\right)}={\left(y_{j1}^{\left(k\right)},\ldots ,y_{jN}^{\left(k\right)}\right)}^{T},N\geq 2$, where the number of cases (i.e., $y_{ji}^{\left(k\right)}=1$) is fixed at *m*, *m* ≥ 1 such that

$$\sum_{i=1}^{N}y_{ji}^{\left(k\right)}=m,k\;\epsilon \;\left\{1,\ldots ,K\right\},j\;\epsilon \;\left\{1,\ldots ,S\right\}.$$ (1)

For each random vector $Y_{j}^{\left(k\right)}$, we have *N* vectors of *P* covariates $X_{j}^{\left(k\right)}={{(X}_{j1}^{\left(k\right)},\ldots ,X_{jN}^{\left(k\right)})}^{T}$ such that $X_{ji}^{\left(k\right)}={\left(x_{ji1}^{\left(k\right)},\ldots ,x_{jiP}^{\left(k\right)}\right)}^{T},i\;\epsilon \;\{1,\ldots ,N\}$. For a given cluster *k* and a given stratum *j*, we suppose that

$$P(y_{ji}^{\left(k\right)}=1|X_{ji}^{\left(k\right)})=\frac{e^{\left(\beta^{T}X_{ji}^{\left(k\right)}\right)}}{\sum_{i=1}^{N}e^{\left(\beta^{T}X_{ji}^{\left(k\right)}\right)}},$$ (2)

where ***β*** = (*β*_1_,…,*β*_*P*_)^*T*^ are the coefficients of the *P* covariates. In the following, we refer to the *N* observations $Y_{j}^{\left(k\right)}=(y_{j1}^{\left(k\right)},\ldots ,y_{jN}^{\left(k\right)})$ as a stratum, and a cluster is then composed of *S* strata.

### Robust estimates of variance using GEE

Inference using generalized estimating equation in conditional logistic regression is explained and illustrated in detail in Craiu et al. [27]; here we develop a brief overview of GEE using notation defined in the previous section. Let *μ*_*k*_ = *E* (***Y***^(***k***)^|***X***^(***k***)^), the mean response for cluster *k*, *k* ∈ {1,…,*K*}, $D_{k}=\frac{d\mu_{k}}{d\beta }$, a derivative matrix of the mean response *μ*_*k*_ with respect to the coefficients ***β*** and ***V***_***k***_ the working covariance matrix of ***Y***^(***k***)^ that is a function of *μ*_*k*_ and a correlation structure specified by the user. A point estimate of ***β***, denoted $\hat{\beta }$, is obtained by solving the generalized estimating equation for ***β***:

$$\sum_{k=1}^{K}D_{k}^{T}V_{k}^{-1}\{Y^{\left(k\right)}-\mu_{k}\}=0.$$ (3)

The naive estimate of variance of $\hat{\beta }$ is given by

$$B={\left(\sum_{k=1}^{K}D_{k}^{T}V_{k}^{-1}D_{k}\right)}^{-1}.$$ (4)

It supposes that the user correctly specified the correlation structure. However, because this correlation structure might be mispecified, the naive estimate of variance of ***β*** can be corrected to produce a robust estimate of variance using the following equation:

$${cov(\hat{\beta })}_{robust}=B\left(\sum_{k=1}^{K}D_{k}^{T}V_{k}^{-1}cov(Y^{\left(k\right)})V_{k}^{-1}D_{k}\right)B,$$ (5)

where, $cov(Y_{j}^{k})$ is the true covariance of ***Y***^(***k***)^ estimated using its empirical version

$${\hat{cov}(Y}^{\left(k\right)})=(Y^{\left(k\right)}-\hat{\mu_{k}}){{(Y}^{\left(k\right)}-\hat{\mu_{k}})}^{T}.$$ (6)

Robust and naive estimates of variance of $\hat{\beta }$ are thus the diagonal values of ${cov(\hat{\beta })}_{robust}$ and ***B***, respectively [28]. A detailed example of use of GEE to estimate step selection functions can be found in Craiu et al. [27].

### Dataset simulation

To test for the effect of the number of clusters (*K*) on robust and naive estimates of variances, we created datasets of a binary response variable that was associated with either two or ten dependent covariates (*P*) and organized into *K* clusters of *S* dependent strata, with each stratum being composed of ten observations (*N* = 10) for which one case (*m* = 1) is associated with nine controls. We varied the number of clusters from one dataset to another, but the total number of observations (*N*_*tot*_) was held constant. As introduced earlier, two sources of autocorrelation can emerge in the response variable: 1) observations from one individual can be more similar than observations from two different individuals; and 2) observations of an individual can be more similar when they have been collected closely in time.

To simulate correlated Bernoulli random variables ${\tilde{Y}}_{t}^{\left(k\right)}$, *t ϵ* {1,…,*T*}, we followed seven steps:

1. We generated *K* cluster-level random intercepts *θ*^(*k*)^, independent and identically distributed (*i*.*i*.*d*) sampled in $Ɲ(0,\sigma_{H}^{2})$.
2. We generated *P* * *K* cluster-level random coefficients $b_{p}^{\left(k\right)},\;p\;\epsilon \;\{1,\ldots ,P\}$, *P* ∈ {2,10}, *i*.*i*.*d* sampled in $Ɲ(0,\sigma_{H}^{2})$.
3. For the *k*^*th*^- cluster, we generated *P* * *T* random coefficients $\gamma_{pt}^{\left(k\right)}$, using an AR(1) model as follows: $\gamma_{pt}^{\left(k\right)}=\rho \;\gamma_{pt-1}^{\left(k\right)}+\varepsilon_{pt}^{\left(k\right)}$, where $\gamma_{p0}^{\left(k\right)}\sim Ɲ(0,1)$ and $\varepsilon_{pt}^{\left(k\right)}\sim Ɲ(0,1)$ i.i.d.
4. We then calculated $\beta_{pt}^{\left(k\right)}=\beta_{p}^{fixed}+b_{p}^{\left(k\right)}+\gamma_{pt}^{\left(k\right)}$.
5. We generated *i*.*i*.*d* covariates $X_{pt}^{\left(k\right)}$, each sampled in $Ɲ(0,\sigma_{X}^{2})$.
6. We calculated $W_{t}^{\left(k\right)}=\theta^{\left(k\right)}+\sum_{p=1}^{P}\beta_{pt}^{\left(k\right)}X_{pt}^{\left(k\right)}$ and $p_{t}^{\left(k\right)}=e^{W_{t}^{\left(k\right)}}/(1+e^{W_{t}^{\left(k\right)}})$.
7. We generated a series of Bernoulli random variables ${\tilde{Y}}_{t}^{\left(k\right)}$, such that $P({\tilde{Y}}_{t}^{\left(k\right)}=1)=p_{t}^{\left(k\right)}$.

Second, we formed each stratum *j*, *j ϵ* {1,…,*S*}, by successively sampling one case (i.e., ${\tilde{Y}}_{t}^{\left(k\right)}=1$) and nine controls (i.e., ${\tilde{Y}}_{t}^{\left(k\right)}=0$) and their associated covariates $X_{1t}^{\left(k\right)}$ … $X_{Pt}^{\left(k\right)}$ within the *k*^*th*^ cluster’s series of Bernoulli random variables ${\tilde{Y}}_{t}^{\left(k\right)}$. We denote the obtained strata $Y_{j}^{\left(k\right)}=(y_{j1}^{\left(k\right)},\ldots ,y_{jN}^{\left(k\right)})$.

Several fixed parameters were held constant for all simulations: $\sigma_{X}^{2}$ = 0.5; *T* = 20 000; *N*_*tot*_ = 600; when *P* = 2: $\beta_{1}^{fixed}=0.75$ and $\beta_{2}^{fixed}=0.5$; when *P* = 10: $\beta_{1}^{fixed}=0.75$; $\beta_{2}^{fixed}=0.75$; $\beta_{3}^{fixed}=0.75$; $\beta_{4}^{fixed}=0.5$; $\beta_{5}^{fixed}=0.5$; $\beta_{6}^{fixed}=0.5$; $\beta_{7}^{fixed}=0.2$; $\beta_{8}^{fixed}=0.2$; $\beta_{9}^{fixed}=0.2$ and $\beta_{10}^{fixed}=0.2$. The number of clusters and the number of strata per cluster varied such that: *K* = {3,5,10,20,30,50} and *S* = *N*_*tot*_/*K*. We varied the strength of inter-individual heterogeneity $\left(\sigma_{H}^{2}\right)$ and temporal autocorrelation (ρ) such that their values cover the usual range of values that are observed in mixed logistic regression in practice. ρ ranges between 0 and 1, and the simulations were based on ρ = {0,0.3,0.5,0.7}. Whereas $\sigma_{H}^{2}$ can range in ℝ^+^, in practice it typically takes values lower than 2. The simulations were thus based on $\sigma_{H}^{2}=\{0,0.2,0.5,1,1.5,2.5\}$.

### Data processing

We tested the effect of *K* on the naive and robust estimates of variance in different scenarios. To do so, we simulated different datasets where the total number of cases (*N*_*tot*_) was held constant but the number of clusters varied following the method described in Dataset simulation. As a result, the number of strata in each cluster depended upon the number of clusters in the dataset. We proceeded in this manner, because in practice, the number of observations is often fixed (e.g., depends on the predetermined schedule of GPS collars). Besides, we were interested in testing the effect of clustering rules on the estimators of the variance of regression coefficient estimates, rather than on the coefficient estimates themselves. The statistical properties depend upon the number of clusters [28], and the number of strata should have much less influence on variance estimates. We included either inter-individual heterogeneity (simulated by between cluster heterogeneity) or temporal autocorrelation (simulated by temporal correlation within clusters), or both in the response variable. We varied the strength of heterogeneity and temporal correlation by respectively changing the values of $\sigma_{H}^{2}$ and ρ: a low value of $\sigma_{H}^{2}\;or\;\rho$ indicates low heterogeneity between clusters or low temporal correlation within clusters, and vice-versa. Once we had created a dataset of *K* clusters, which were each composed of *S* strata, we ran the GEE analysis that is described in the following section (see Statistical analysis).

**Fig 1:**
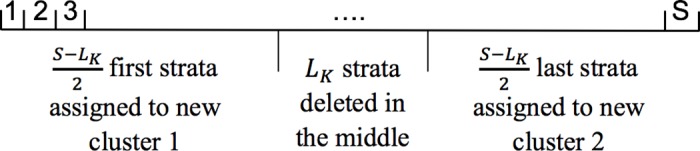
Details on how to resample datasets using destructive sampling. *S* represents the number of strata from one individual. *L*_*K*_ represents the lag i.e., the number of strata to remove to meet the assumption of temporal independence.

Destructive sampling is a common strategy that is used to increase the number of clusters for GEE analysis. Thus, we tested the effect of this method on the robust and naive estimates of variances when varying the number of clusters. Initially, we simulated 500 datasets of *K* clusters and *S* strata with either between cluster heterogeneity or within cluster temporal autocorrelation or both (see Statistical analysis). Following Forester et al. [25], we then estimated the lag beyond which there is no longer significant temporal correlation for each dataset. The maximum lag (*L*_*K*_) among the 500 was used to resample each dataset: for each cluster, we kept the first (*S* − *L*_*K*_)/2 successive strata that we assigned to a new cluster. We then dropped the next *L*_*K*_ successive strata and kept the last (*S* − *L*_*K*_)/2 successive strata that we had assigned to a new cluster (Fig 1). Thus, we obtained 2*K* clusters that were composed of (*S* − *L*_*K*_)/2 strata. Once we had reorganized the dataset, we ran the GEE analysis from the section Statistical analysis.

It is also common to have an unbalanced dataset (i.e., the number of observations that were collected per individual varies), which we modelled as a dataset of *K* clusters with different number of strata *S*^(*k*)^. We tested the effect of having unbalanced datasets on the robust and naive estimates of variance when varying the number of clusters. We created a dataset of *K* clusters and *S* strata with either between cluster heterogeneity or within cluster temporal autocorrelation, or both. We then proceeded to one of the following unbalancing: 1) we selected one-third of the *K* clusters in the initial dataset and retained only the first half of their strata, i.e., 2*K*/3 clusters with *S* strata and *K*/3 clusters with *S*/2 strata, thereafter referred to as weakly unbalanced dataset; 2) we selected two-thirds of the *K* clusters in the initial dataset and retained only the first quarter of the strata for the first third and the first half of the strata for the second third, i.e. *K*/3 clusters with *S* strata, *K*/3 clusters with *S*/2 strata and *K*/3 clusters with *S*/4 strata, thereafter referred to as strongly unbalanced dataset. We analyzed the resulting unbalanced dataset.

### Statistical analysis

From the simulated binary response vector $Y_{j}^{\left(k\right)}$ and its associated covariates $X_{1j}^{\left(k\right)}$, …, $X_{Pj}^{\left(k\right)}$, *P ∈* {2, 10} we estimated ${\hat{\beta }}_{p},p$ ϵ {1, …, P} by solving Eq 3, and computed their respective robust (denoted *V*_*R*_) and naive (denoted *V*_*N*_) variance estimates using the function *coxph* in the ‘survival’ package [29] which is available from the Comprehensive R Archive Network (CRAN).

We ran *R* = 500 simulations and obtained 500 estimates of ${\hat{\beta }}_{pr},\;p$ ϵ {1, …, P}, *r* ϵ {1, …, 500}, for each scenario. We first checked that coefficient estimates ${\hat{\beta }}_{pr}$ remained consistent regardless of clustering schemes by averaging the 500 estimates (S3 Fig). Then, the Monte Carlo estimation of the true variances (denoted *V*_*T*_) of estimates ${\hat{\beta }}_{pr}$, were calculated using

$$V_{T_{p}}=\frac{1}{R-1}\sum_{r=1}^{R}{\left({\hat{\beta }}_{pr}-\frac{1}{R}\sum_{r=1}^{R}{\hat{\beta }}_{pr}\right)}^{2},$$ (7)

To evaluate if the robust and naive estimates of variance are good estimators of the true variance, we calculated the average ratios $V_{R_{p}}/V_{T_{p}}$ and $V_{N_{p}}/V_{T_{p}}$ over the 500 simulations [16]. Ratios that were close to 1 reflect small estimation errors. An unbiased estimator should have an average ratio that is not significantly different from 1. For the sake of simplicity, we thereafter drop index *p*.

## Results

### Naive estimate of variance

**Fig 2:**
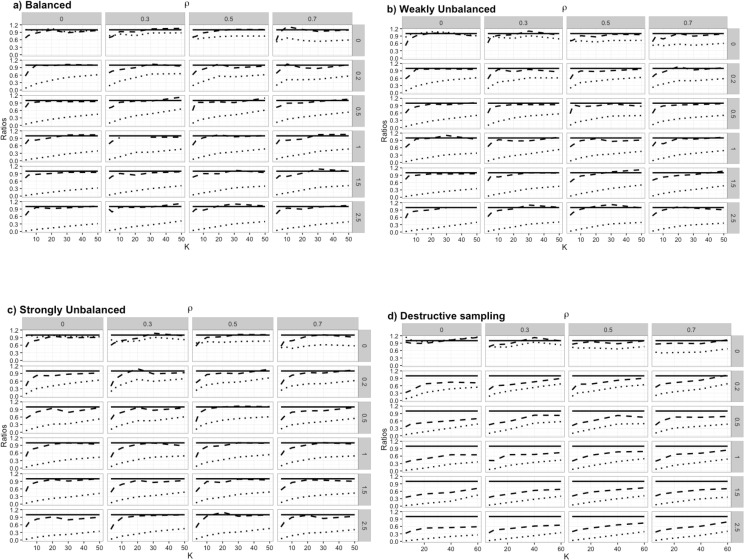
**Comparison of average ratios between robust estimates of variance (*V*_*R*_/*V*_*T*_, dashed lines) or naive estimates of variance over true variance (*V*_*N*_/*V*_*T*_, dotted lines) of coefficient** ${\hat{\beta }}_{1}$ **when *P* = 10 for different number of clusters (*K*), as a function of temporal autocorrelation (*ρ*) and inter-individual heterogeneity (**$\sigma_{H}^{2}$ **on the right side of the panels), as well as different data processing: a) Balanced, each cluster has the same number of strata (*S* = *N*/*K*); b) Weakly Unbalanced, *K*/3 clusters have *S*/2 strata and 2*K*/3 clusters have *S* strata; c) Strongly Unbalanced, *K*/3 clusters have *S*/4 strata, *K*/3 clusters have *S*/2 strata and *K*/3 clusters have *S* strata; d) Destructive sampling, each initial cluster of *S* strata has been split into 2 clusters, a variable number of strata had been dropped in between to meet the assumption of independence between clusters.** Robust or naive estimates of variance are unbiased when ratios are not significantly different from 1 at the 5% level (solid line).

When datasets were simulated without correlation in the response variable (i.e., *ρ* = 0 and $\sigma_{H}^{2}=0$), the naive variance was nearly equal to the true variance (average *V*_*N*_/*V*_*T*_ ≈ 1), independent of the number of covariates (i.e., *P* = {2,10}), the number of clusters and the manner in which the data were processed (balanced, weakly unbalanced, strongly unbalanced or destructive sampling, Fig 2). When considering the other scenarios (i.e., *ρ* > 0 or $\sigma_{H}^{2}>0$), the naive variance systematically underestimated the true variance by at least 17% for any of the model’s covariates (i.e., average *V*_*N*_/*V*_*T*_ never exceeded 0.83 for any ${\hat{\beta }}_{p},p\in \{1,\ldots ,P\},\;P=\{2,10\}$), regardless of type of data processing (Fig 2 for first coefficient (${\hat{\beta }}_{1}$) of model including ten covariates, S2 Fig for the remaining ${\hat{\beta }}_{p},\;1\leq p\leq P$).

### Inter-individual heterogeneity

When inter-individual heterogeneity was included in the response variable (i.e., *ρ* = 0 and $\sigma_{H}^{2}>0$), the bias of the robust estimate of variance depended upon the number of clusters (Figs 2 and S2). Regardless of the number of covariates (i.e., *P* = {2,10}) and whether or not the number of observations per cluster was fixed, the average ratio *V*_*R*_/*V*_*T*_ increased with the number of clusters towards an asymptote of 1, which was essentially reached with 20 independent clusters. With further increases in the number of clusters, the average ratio fluctuated around the value 1, meaning that the lowest bias was attained (Fig 2A, 2B and 2C). With 20 clusters, however, the robust estimate of variance still had rather low precision (i.e., large fluctuations between simulations independently of the number of parameters, S1 Fig), and precision continued to increase up until approximately 30 independent clusters were used (S1 Fig).

When using destructive sampling (i.e., cluster split into smaller clusters by removing strata), the robust estimate of variance systematically underestimated the true variance for datasets that included inter-individual heterogeneity, but not temporal autocorrelation (i.e., $\sigma_{H}^{2}>0$ and *ρ* = 0, Fig 2D). Indeed, the robust variance underestimated the true variance by at least 18% (i.e., average *V*_*R*_/*V*_*T*_ never exceeded 0.82 for any ${\hat{\beta }}_{p}$, *p* ∈ {1,…,*P*}), regardless of the number of covariates, the number of clusters and the strength of inter-individual heterogeneity (Fig 2D for ${\hat{\beta }}_{1}$ of model including ten covariates, S2D Fig for the remaining ${\hat{\beta }}_{p}$, 1 ≤ *p* ≤ *P*).

### Temporal autocorrelation with or without inter-individual heterogeneity

When observations of the response variable were temporally autocorrelated (i.e., *ρ* > 0), the bias in the robust estimate of variance could be largely corrected with the use of at least 20 independent clusters regardless of the number of covariates and inter-individual heterogeneity (i.e., *P* = {2,10} and $\sigma_{H}^{2}\geq 0$, Figs 2 and S2). Also the precision of the robust estimate of variance largely increased until 30 independent clusters were used (S1 Fig). These results hold for both balanced and unbalanced datasets (Figs 2, S1 and S2).

When using a destructive sampling scheme with autocorrelated response variables (i.e., *ρ* > 0), we obtained a robust estimate of variance without significant bias only for a certain range of inter-individual heterogeneity and a certain number of clusters after having split the data. Specifically, 60 clusters were necessary to get unbiased robust estimate of variance when $\sigma_{H}^{2}\leq 0.2$ independently of the strength of temporal autocorrelation. When $\sigma_{H}^{2}>0.2$, the robust variance systematically underestimated the true variance regardless of the number of clusters included in the analysis. This finding holds independently of the number of covariates included in the regression model (Figs 2 and S2).

### Coefficient estimates

S3 Fig shows that the sampling design (data processing, number of clusters) does not have any impact on the average value of the coefficient estimates ($\hat{\beta }$), which remain consistent estimators of the marginal covariate effects. They do illustrate, however, how the difference between the marginal effects and the conditional effects (values of $\beta_{p}^{fixed},p\;\epsilon \;\{1,\ldots ,P\}$, used in the simulations) increases as the heterogeneity or autocorrelation increase (see detailed discussion of this phenomenon in Craiu et al [19] and Fieberg et al. [17]).

## Discussion

Conditional logistic regression (CLR) is frequently used to analyze animal movements and habitat selection [7], but the lack of clear guidelines that would insure the robustness of statistical models may hamper the gain of ecological knowledge. Our simulations can provide guidance to minimize the risk of bias when estimating the variance of CLR parameters from correlated field observations. With the rapid technological advances that have taken place in recent years (e.g., GPS collars getting smaller, geographic information system with progressively higher resolution; [13]), the need to correct for biases in CLR variance estimates induced by autocorrelation is likely to become increasingly common and for a larger range of species, especially in light of recent studies that highlight the advantages of using resource and step selection functions that are derived from CLR [12,25]. Our simulation study shows how to obtain robust resource and step selection functions estimates of variance parameters with such datasets. Simulations of longitudinal data revealed that: 1) a rather small number of clusters is required to obtain unbiased variance estimation of CLR parameter estimates even when the number of covariates is large; 2) clusters can be created with destructive sampling, but only under specific circumstances; and 3) robust estimates of variance for CLR parameters can be obtained even with unbalanced datasets. We discuss each of these simulation outputs to provide general guidelines for the use of CLR.

Simulations show that the robust estimate of variance becomes unbiased when the number of independent clusters is higher than 20, regardless of the strength of inter-individual heterogeneity or temporal autocorrelation and the number of parameters considered in habitat selection studies. However, variation in robust estimates of variance kept decreasing even when the number of independent clusters exceeded 20, but the gain in precision became much less noticeable past 30 clusters. Because the precision of the robust variance increased with the number of clusters, analyses should be conducted with as many independent clusters as possible to attain maximum precision [30]. Logistic and financial constraints, however, often restrict the number of individuals that can be monitored in ecological studies. Ziegler et al. [31] suggested that at least 30 independent clusters should be used when they are formed of 4 strata for a low to moderate degree of correlation to fit logistic regression with GEE. They further suggested the use of an even greater number of clusters for a high degree of correlation. Yet the authors did not base their conclusions on simulations as we did. We tested a broad range of correlations and still found that 30 independent clusters remained sufficient to draw robust inferences in habitat selection and movement studies that were based on CLR, even when habitat selection is based on several parameters and data are sampled at high rates with strong behavioural plasticity among individuals. This finding can be helpful to fix and justify the number of individuals to monitor when setting-up habitat selection or movement studies.

The number of independent individuals may not always be sufficiently large to obtain reasonably robust variances (i.e., less than 30 independent individuals), in which case the dataset can be resampled using destructive sampling to increase the number of clusters according that the sampling frequency is high and the behavioural plasticity among individuals is low. If applied when there is no temporal autocorrelation or when individuals have largely distinct behaviours, the robust estimate of variance remains biased, and conclusions regarding resource selection behaviour may be unreliable. Thus, an assessment of the presence of temporal autocorrelation (using an autocorrelation function for example, see [25]) and inter-individual heterogeneity (using individual-level random coefficients, see [19]) should be performed before using destructive sampling.

Whereas destructive sampling should remove statistical bias in temporally autocorrelated datasets with low heterogeneity among individuals, the process can reduce statistical power when a large proportion of the data are dropped. For example, removing 95% of the initial dataset led to a change in conclusions on habitat selection by woodland caribou (*Rangifer tarandus caribou*) compared to analyses that were based upon the entire dataset [16]. Reducing sample size not only decreases statistical power in such extreme cases [16,32], it can also lead to the loss of biological information [33]. Therefore, the analysis should consider the compromise between the need to obtain robust inferences on CLR parameters by excluding data to create statistically independent clusters, and the need to maintain high power to clarify the movement or habitat selection behaviours by retaining as many observations as possible. The number of observations should be dropped in accordance with the number of clusters that are necessary to get robust inference. In retrospect, a number of studies might have discarded an excessive number of field observations. For example, Babin et al. [34] resampled their initial dataset, which was composed of GPS locations from 8 individuals, by dropping repeatedly segments of 20 successive locations until they obtained 112 clusters of 7 strata per individual. By doing so, they dropped 75% of the initial data while they could have dropped less than 5% by creating 64 clusters (i.e., 8 clusters for each individual) that would potentially be needed to obtain robust estimates of variance for the CLR parameters.

The simulations also demonstrated the effectiveness of GEE in correcting for biases in the variance of CLR parameters even when the number of observations differs among individuals. We showed for two different magnitudes of unbalanced datasets that 30 clusters were still sufficient to correct for the bias. Fitzmaurice *et al*. [30] pointed out, however, that GEE might not be reliable when the design is extremely unbalanced. In fact, when datasets become highly unbalanced by including individuals with only few observations, issues may arise, not only with respect to the statistical analysis, but also with the ecological information. Because animals with few observations may offer relatively poor information about space use patterns in the first place [35], a common approach is then to discard individuals with too few observations [36]. Moreover, those individuals might not be comparable to individuals followed over extended time periods because animal-habitat relationships tend to vary over time [37,38]. This is why, in practice, most studies have datasets with a number of observations that are rather similar among individuals [11,39,40], in which case our simulations showed that GEE would be an effective approach to remove biases in CLR variance estimates and robust inferences can be made without losing biological information by removing individuals.

## Supporting Information

S1 Appendix Zipped folder containing R codes used to do the simulations and a description file (Readme.txt). (ZIP)

S1 Fig**Ninety-five percent confidence intervals of average ratios between robust estimates of variance over true variance (light grey) of coefficients** ${\hat{\beta }}_{p}$ **for different number of covariates (*P*) and different number of clusters (*K*), as a function of different strengths of temporal autocorrelation (*ρ*) and inter-individual heterogeneity (**$\sigma_{H}^{2}$ **on the left side of the panels) as well as different data processing: a) Balanced, b) Weakly Unbalanced, c) Strongly Unbalanced and d) Destructive sampling.** Confidence intervals have been calculated using a non-parametric method: upper and lower bounds are the 0.975 and 0.025 quantiles of the 500 observed *V*_*R*_/*V*_*T*_’s, respectively. Average ratios between robust estimates of variance and true variances (*V*_*R*_/*V*_*T*_) of coefficient ${\hat{\beta }}_{p}$ are represented by dashed lines on the figure.(PDF)

S2 Fig**Comparison of average ratios between robust estimates of variance (*V*_*R*_/*V*_*T*_, dashed lines) or naive estimates of variance over true variance (*V*_*N*_/*V*_*T*_, dotted lines) of coefficients** ${\hat{\beta }}_{p}$**, for different number of covariates (*P*) and different number of clusters (*K*), as a function of temporal autocorrelation (*ρ*) and inter-individual heterogeneity (**$\sigma_{H}^{2}$ **on the left side of the panels) as well as different data processing: a) Balanced, b) Weakly Unbalanced, c) Strongly Unbalanced and d) Destructive sampling.** Robust or naive estimates of variance are unbiased when ratios are not significantly different from 1 (solid line).(PDF)

S3 Fig**Average estimates of** ${\hat{\beta }}_{p}$ **for different number of covariates (*P*) and different number of clusters (*K*), as a function of temporal autocorrelation (*ρ*) and inter-individual heterogeneity (**$\sigma_{H}^{2}$ **on the left side of the panels) as well as different data processing: a) Balanced, b) Weakly Unbalanced, c) Strongly Unbalanced and d) Destructive sampling.** Fixed values of *β*_*p*_, *p* ∈ {1,…,*P*} were equal to: $\beta_{1}^{fixed}=0.75$ and $\beta_{2}^{fixed}=0.5$ when *P* = 2 and $\beta_{1}^{fixed}=0.75$; $\beta_{2}^{fixed}=0.75$; $\beta_{3}^{fixed}=0.75$; $\beta_{4}^{fixed}=0.5$; $\beta_{5}^{fixed}=0.5$; $\beta_{6}^{fixed}=0.5$; $\beta_{7}^{fixed}=0.2$; $\beta_{8}^{fixed}=0.2$; $\beta_{9}^{fixed}=0.2$ and $\beta_{10}^{fixed}=0.2$ when *P* = 10.(PDF)
