# Supplementary material for: Robust Inference from Conditional Logistic Regression Applied to Movement and Habitat Selection Analysis
Source: PLoS One. 2017 Jan 12;12(1):e0169779. doi: 10.1371/journal.pone.0169779 (PMC5233429; doi:10.1371/journal.pone.0169779)
Supplement: S1 Fig — Ninety-five percent confidence intervals of average ratios between robust estimates of variance over true variance (light grey) of coefficients β^p for different number of covariates (P) and different number of clusters (K), as a function of different strengths of temporal autocorrelation (ρ) and inter-individual heterogeneity (σH2 on the left side of the panels) as well as different data processing: a) Balanced, b) Weakly Unbalanced, c) Strongly Unbalanced and d) Destructive sampling. Confidence intervals have been calculated using a non-parametric method: upper and lower bounds are the 0.975 and 0.025 quantiles of the 500 observed VR/VT’s, respectively. Average ratios between robust estimates of variance and true variances (VR/VT) of coefficient β^p are represented by dashed lines on the figure. (PDF) [file pone.0169779.s002.pdf]

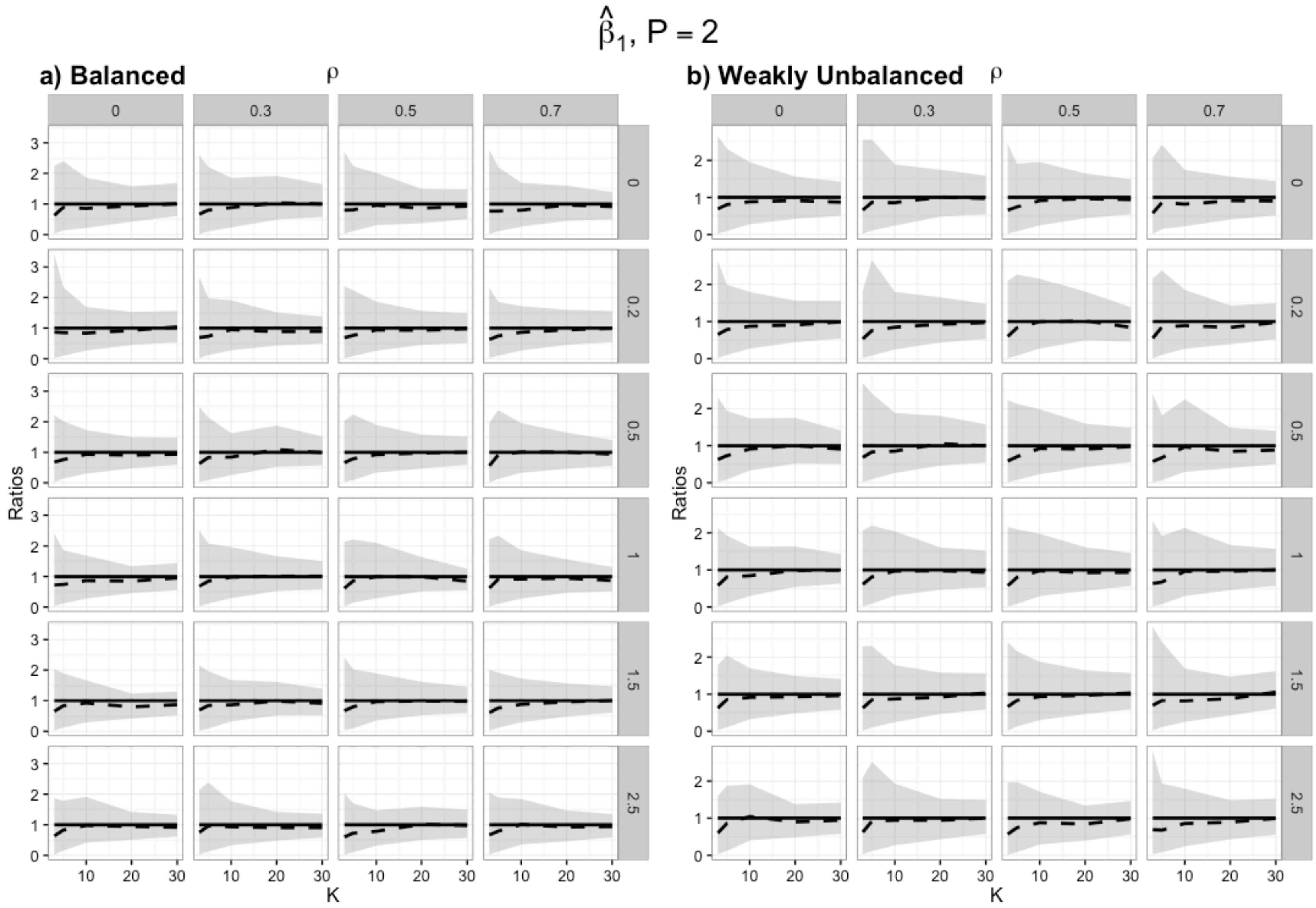

$\hat{\beta}_1$, P = 2
a) Balanced
b) Weakly Unbalanced
ρ
0
0.3
0.5
0.7
0.2
1
1.5
2.5
Ratios
K
10
20
30

1

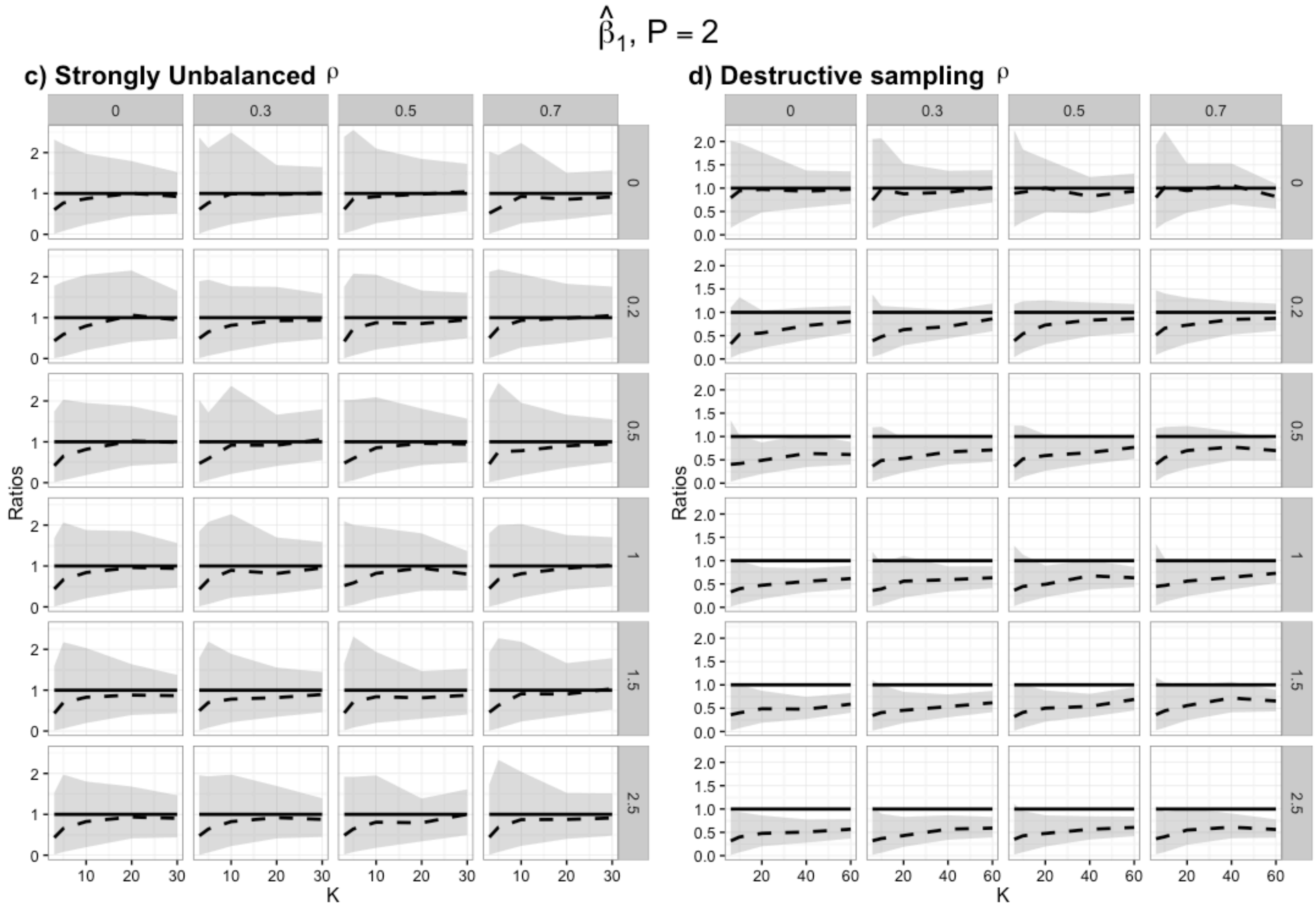

$\hat{\beta}_1$, P = 2
c) Strongly Unbalanced ρ
d) Destructive sampling ρ
0
0.3
0.5
0.7
0.2
1
1.5
2.5
Ratios
K

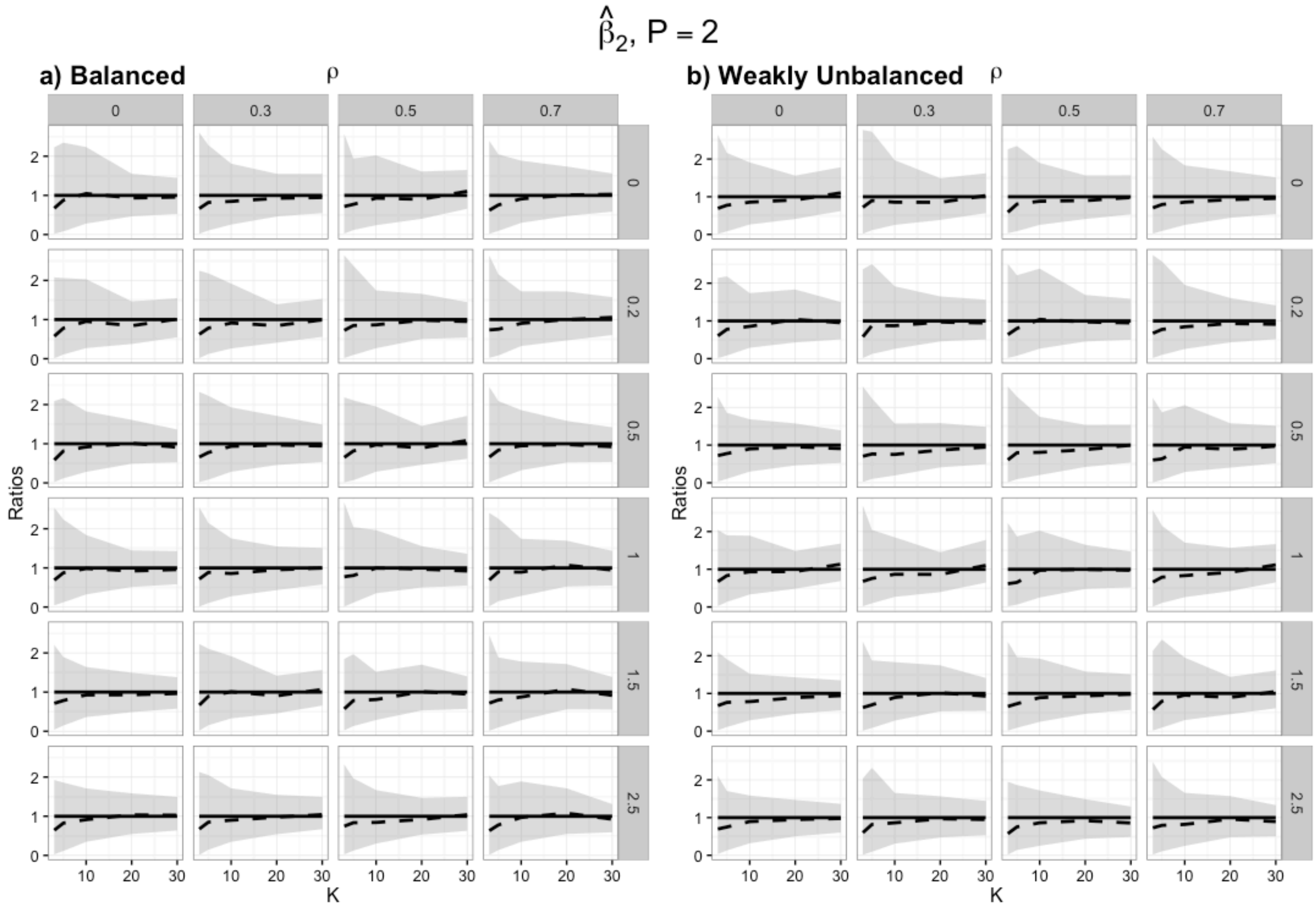

$\hat{\beta}_2$, P = 2
a) Balanced
b) Weakly Unbalanced
ρ
0
0.3
0.5
0.7
0.2
1
1.5
2.5
Ratios
K
10
20
30

3

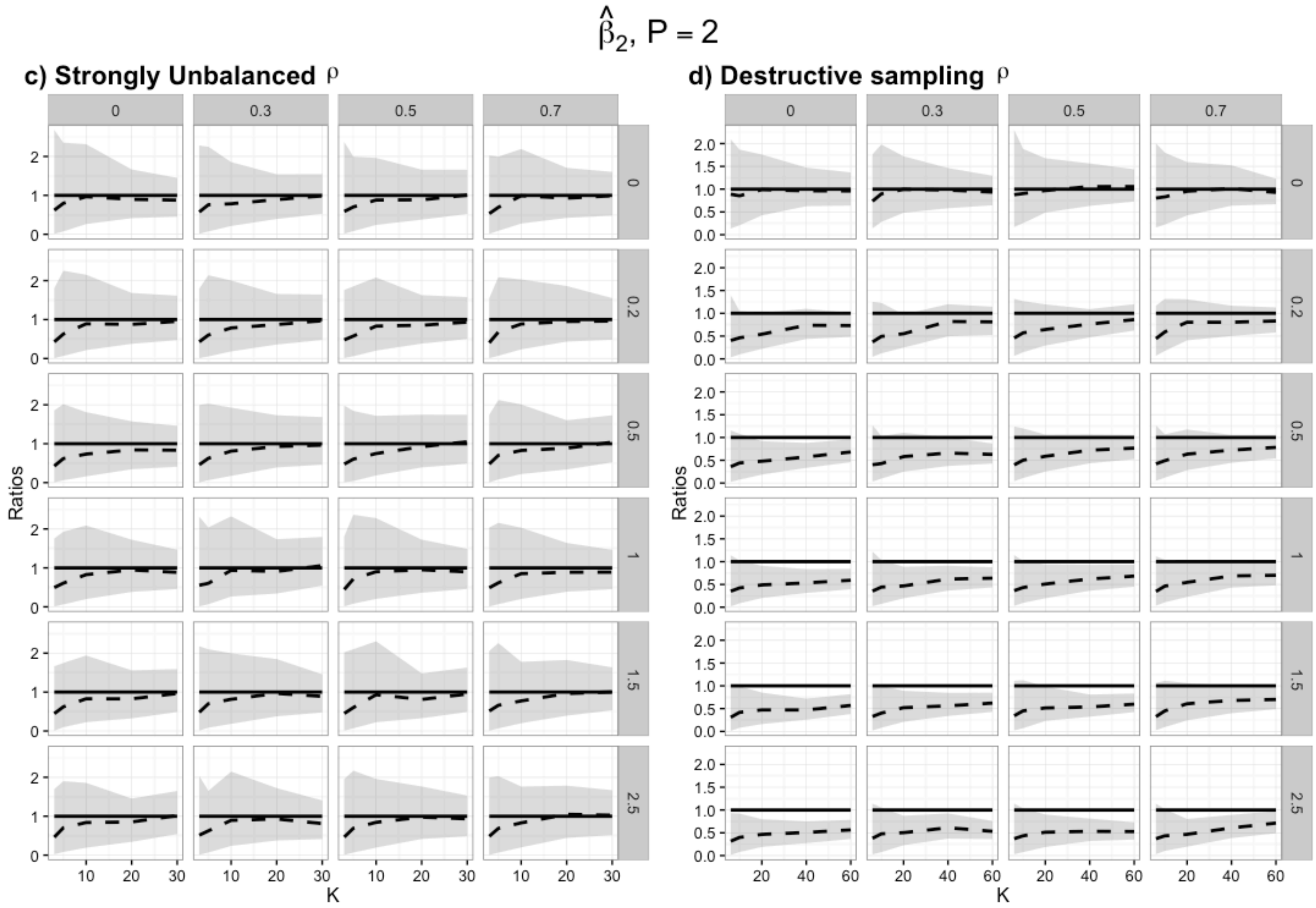
$\hat{\beta}_2$, P = 2
c) Strongly Unbalanced ρ
d) Destructive sampling ρ
0
0.3
0.5
0.7
0
0.2
0.5
1
1.5
2.5
Ratios
K

4

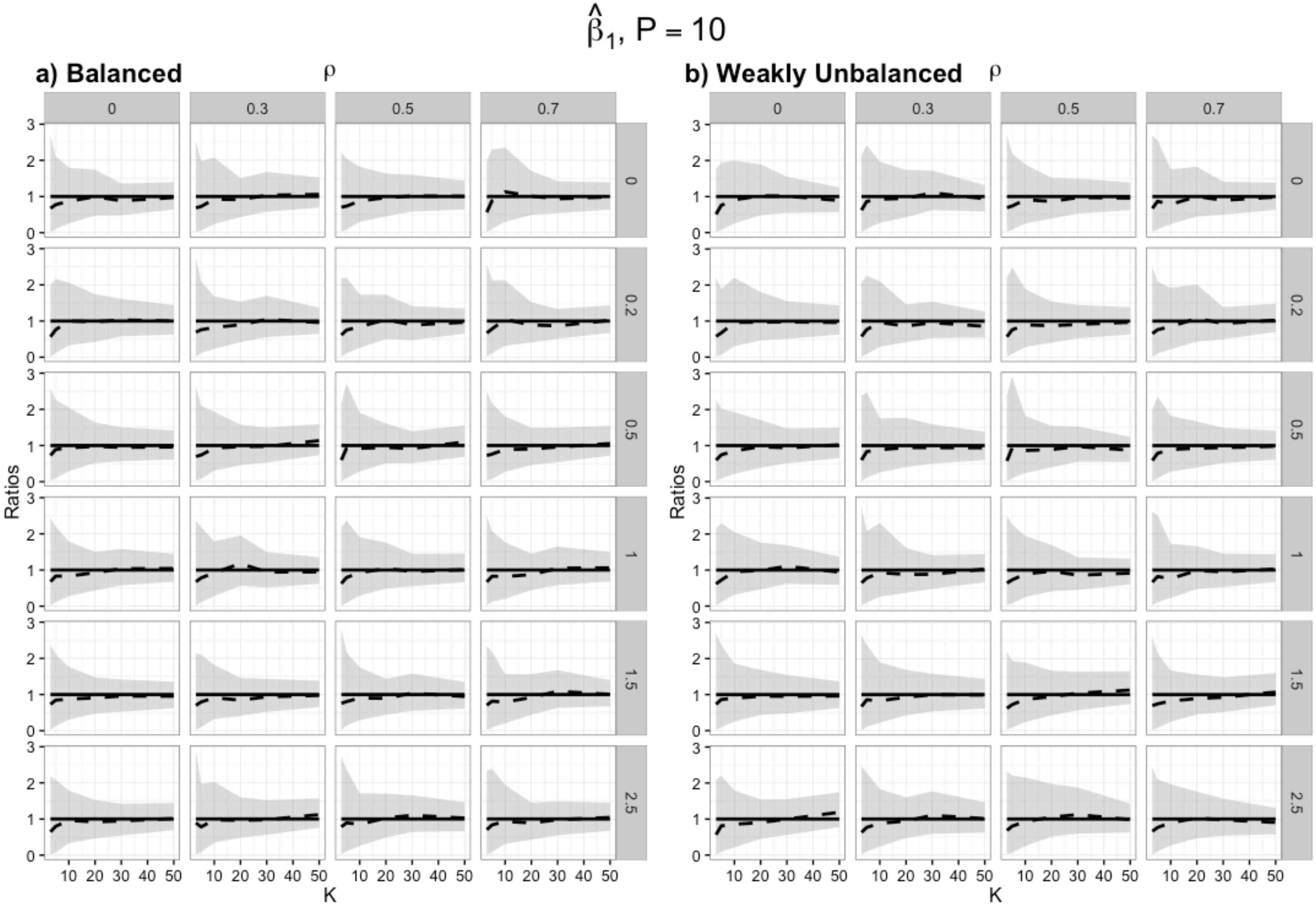

$\hat{\beta}_1$, P = 10
a) Balanced
b) Weakly Unbalanced
ρ
0
0.3
0.5
0.7
0
0.2
0.5
1
1.5
2.5
Ratios
K
10 20 30 40 50
0
1
2
3

5

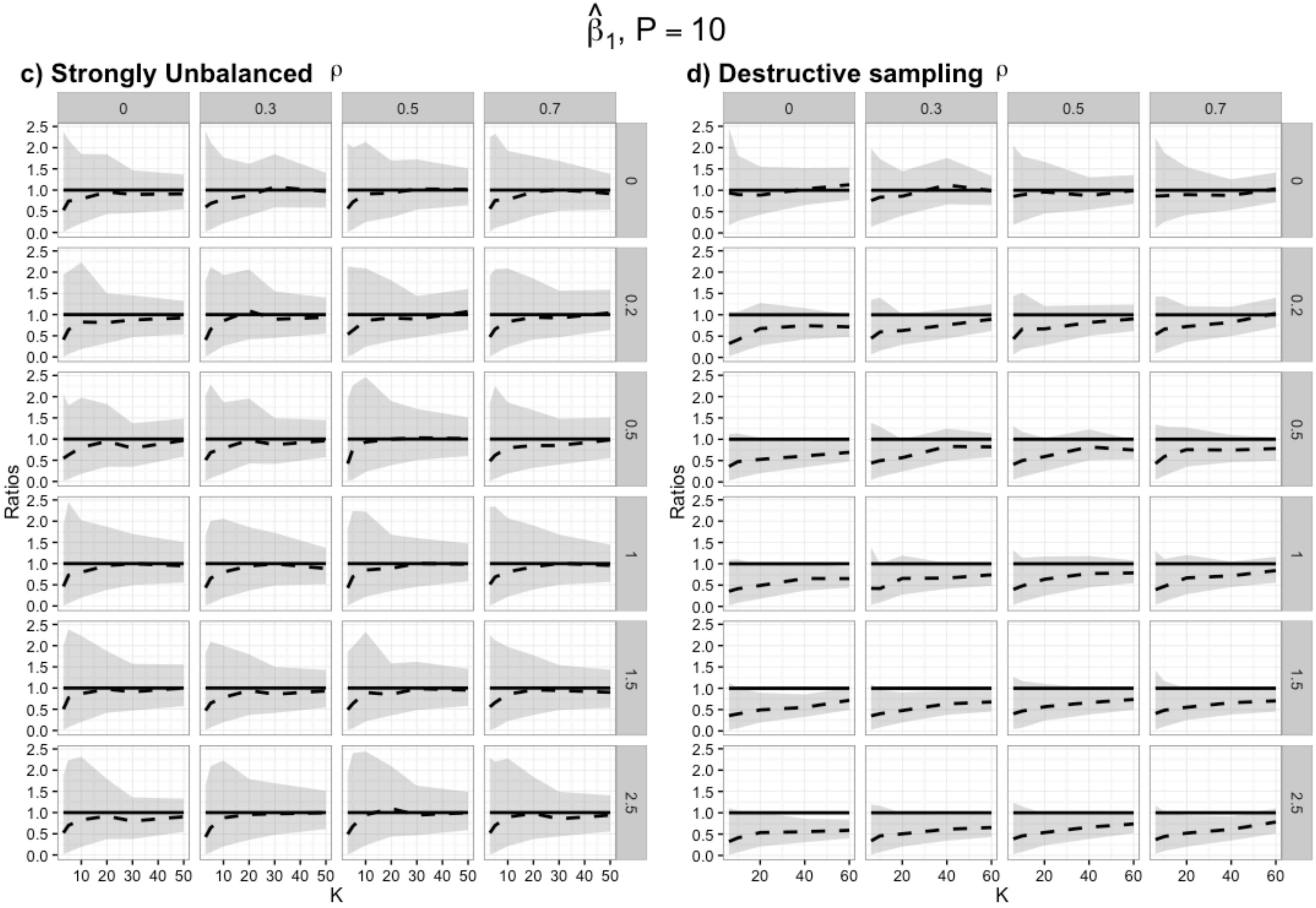
$\hat{\beta}_1$, P = 10
c) Strongly Unbalanced ρ
d) Destructive sampling ρ
0
0.3
0.5
0.7
0
0.2
0.5
1
1.5
2.5
Ratios
0.0 0.5 1.0 1.5 2.0 2.5
10 20 30 40 50
20 40 60
K

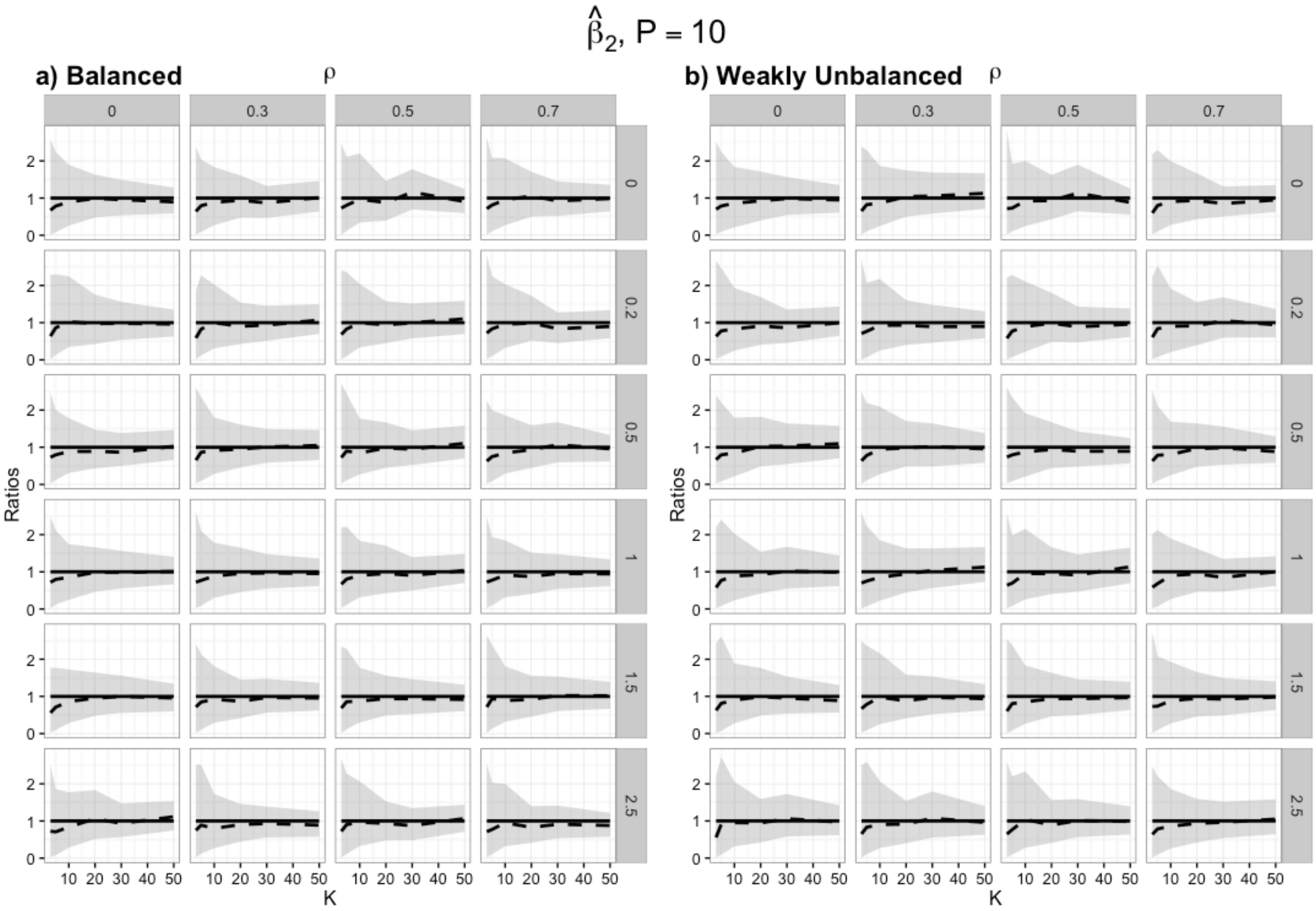

$\hat{\beta}_2$, P = 10
a) Balanced
b) Weakly Unbalanced
ρ
0
0.3
0.5
0.7
0
0.2
0.5
1
1.5
2.5
Ratios
K
10 20 30 40 50

7

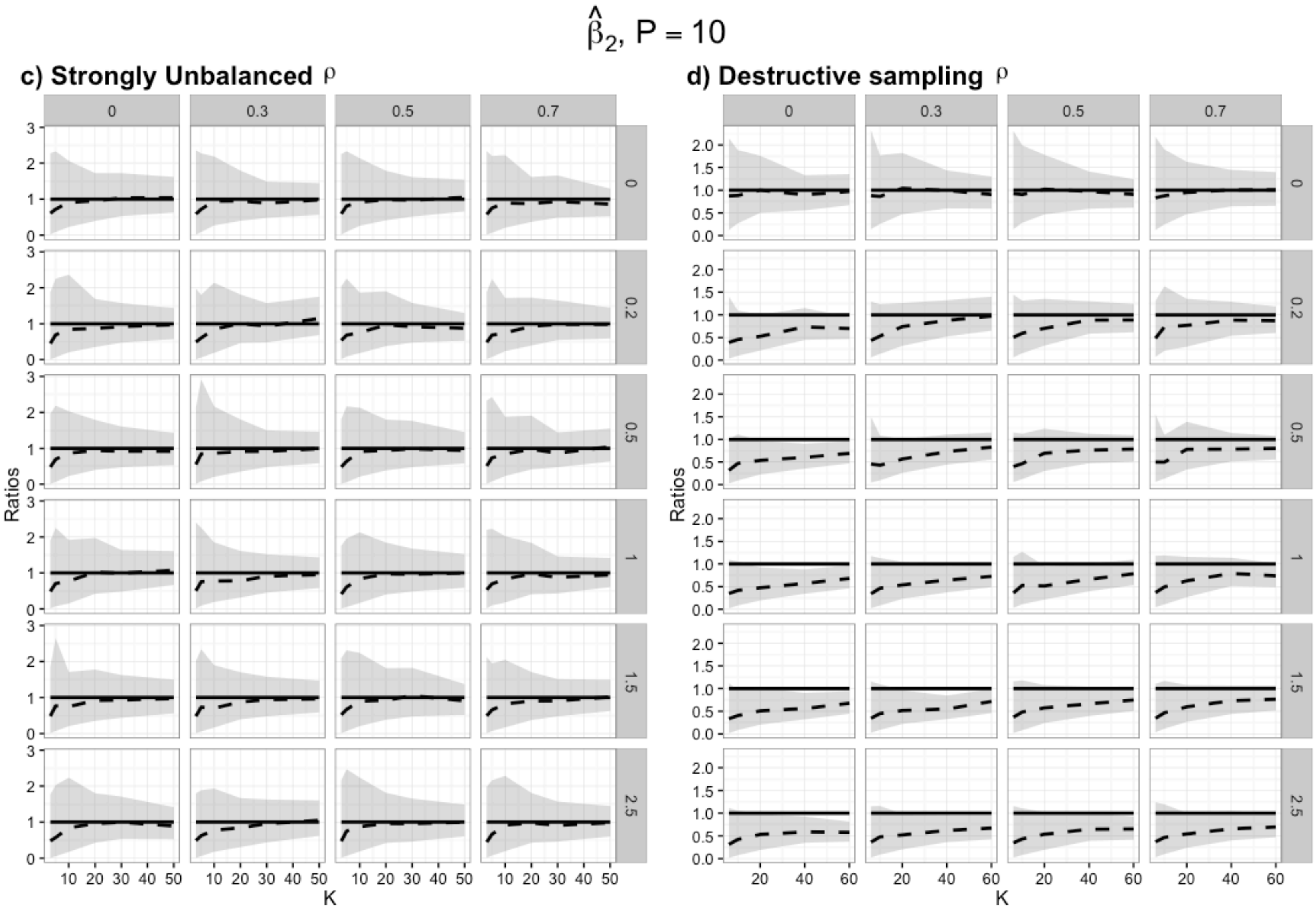

$\hat{\beta}_2$, P = 10
c) Strongly Unbalanced ρ
d) Destructive sampling ρ
0
0.3
0.5
0.7
0.2
1
1.5
2.5
Ratios
K

8

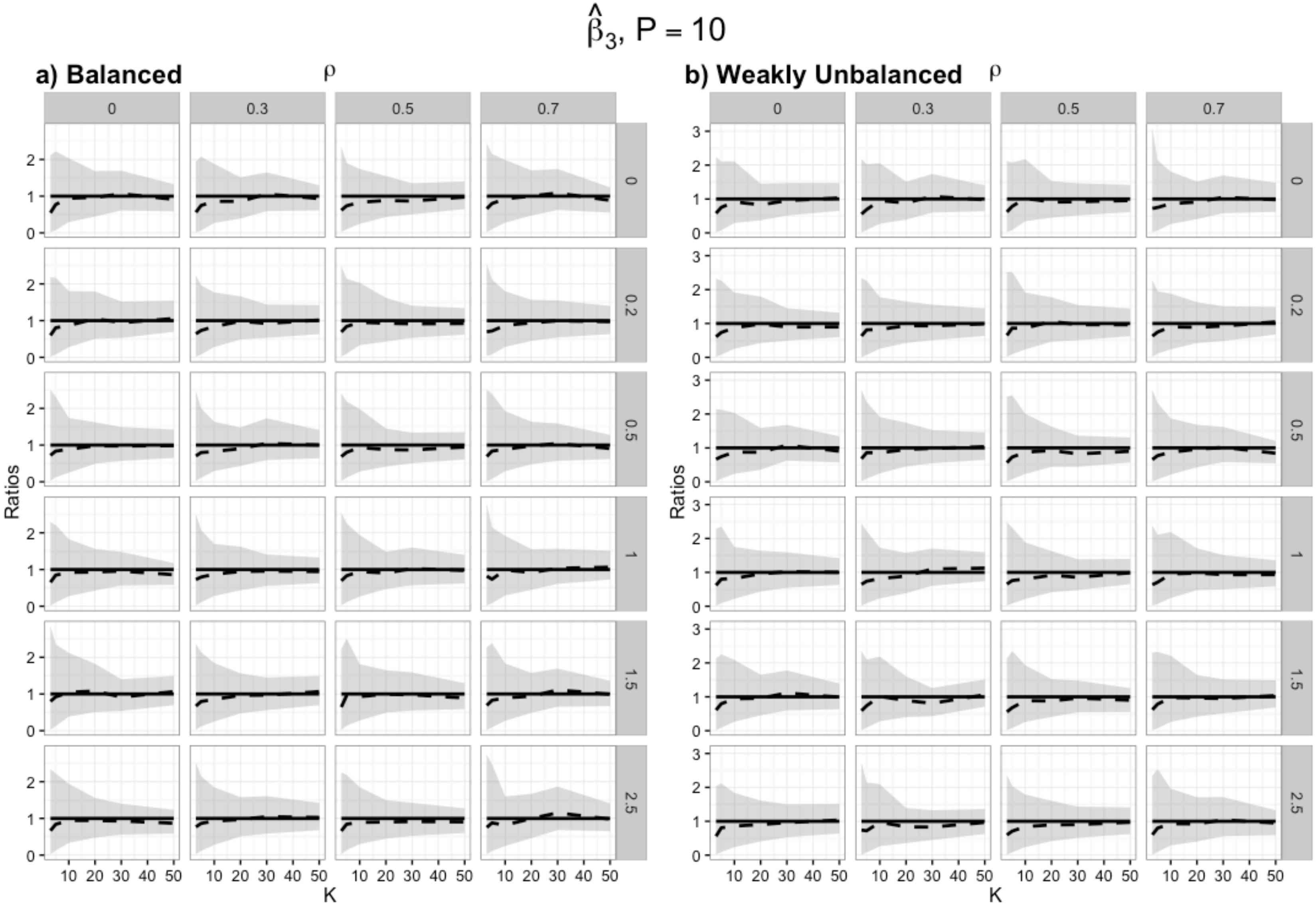
$\hat{\beta}_3$, P = 10
a) Balanced
b) Weakly Unbalanced
ρ
0
0.3
0.5
0.7
0
0.2
0.5
1
1.5
2.5
Ratios
K

9

$\hat{\beta}_3$, P = 10

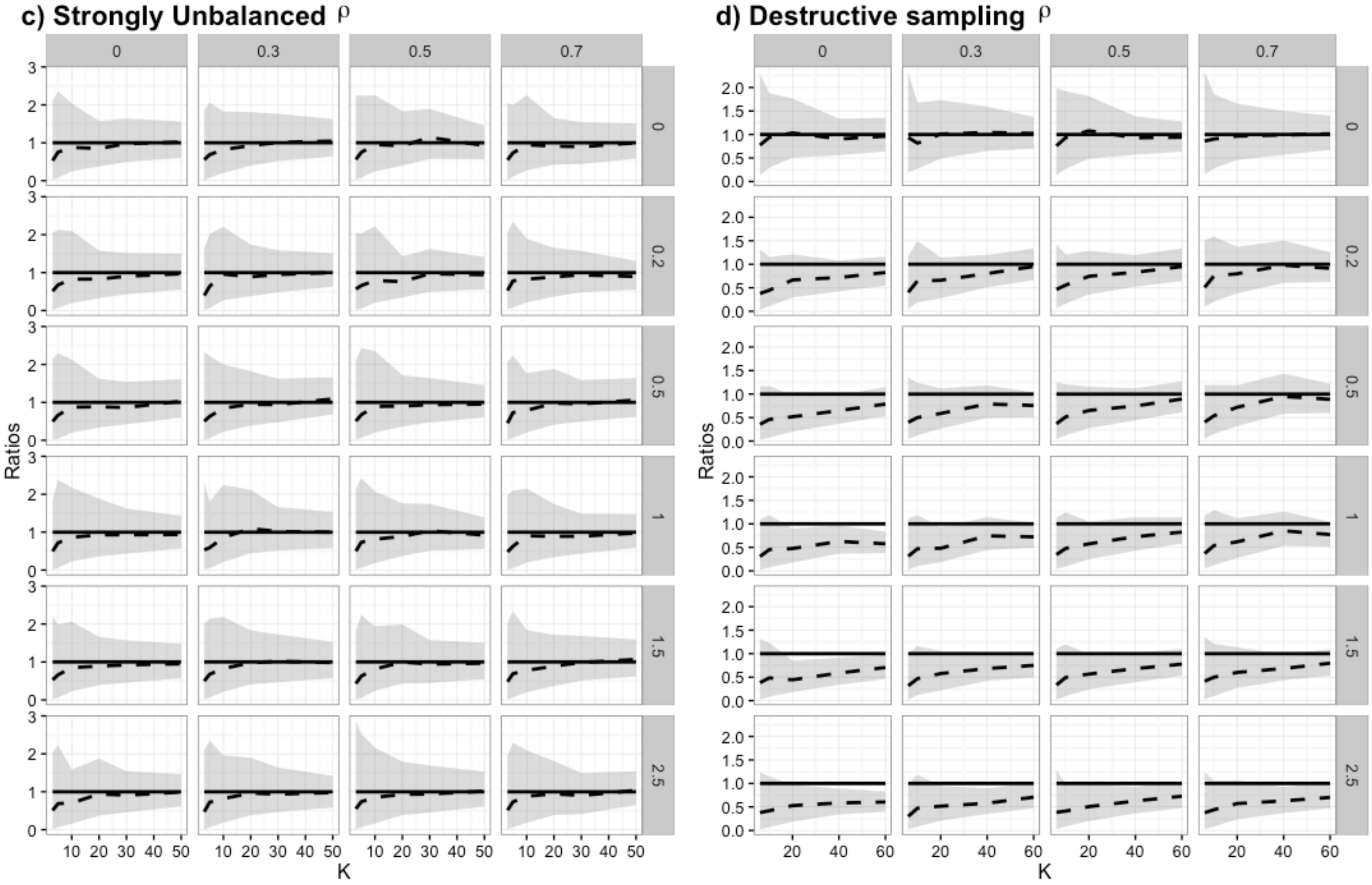

10

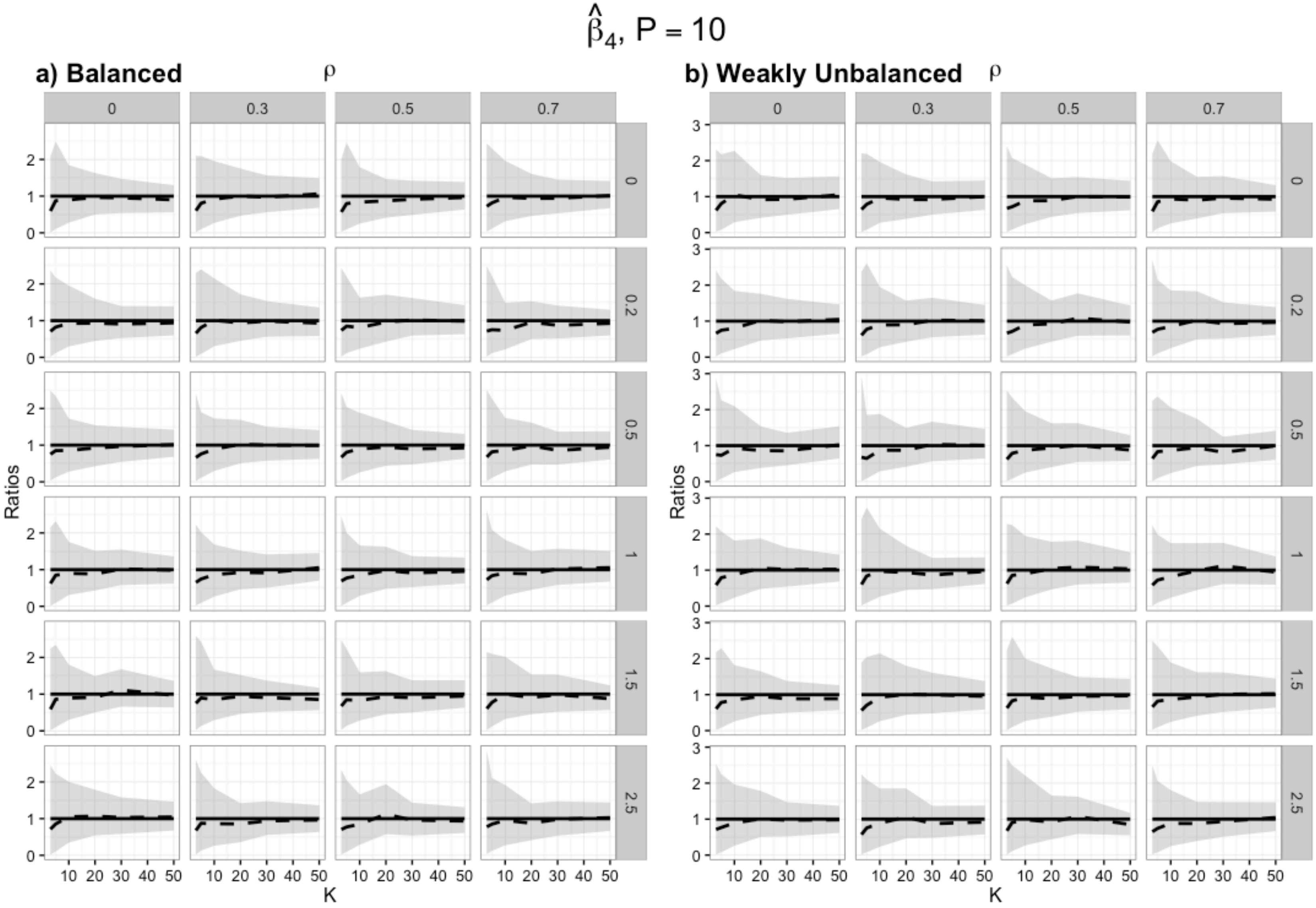

$\hat{\beta}_4$, P = 10
a) Balanced
b) Weakly Unbalanced
ρ
0
0.3
0.5
0.7
0.2
1
1.5
2.5
Ratios
K
10 20 30 40 50

11

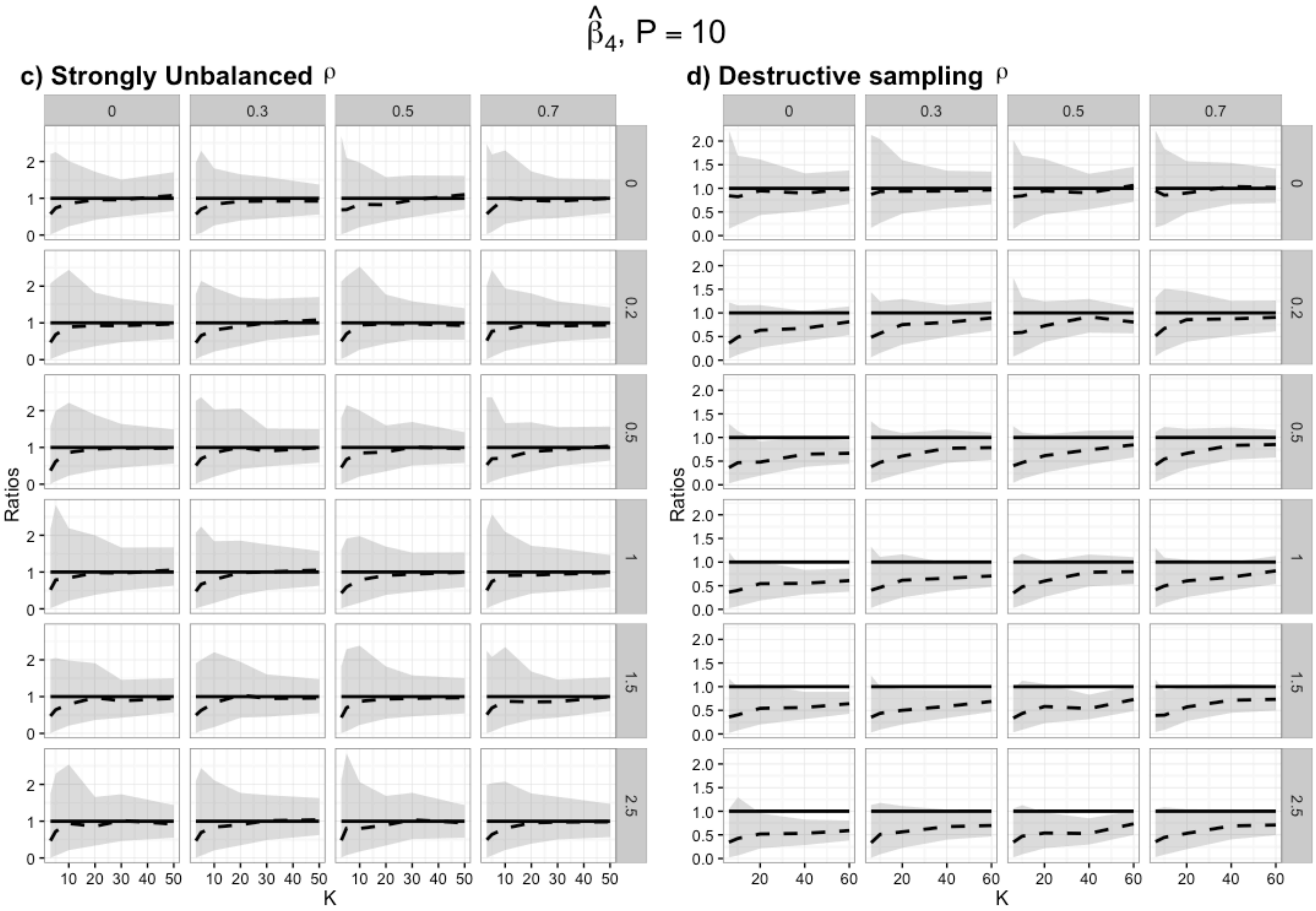

$\hat{\beta}_4$, P = 10
c) Strongly Unbalanced ρ
d) Destructive sampling ρ
0
0.3
0.5
0.7
0
0.2
0.5
1
1.5
2.5
Ratios
K
10 20 30 40 50
20 40 60
0.0 0.5 1.0 1.5 2.0

12

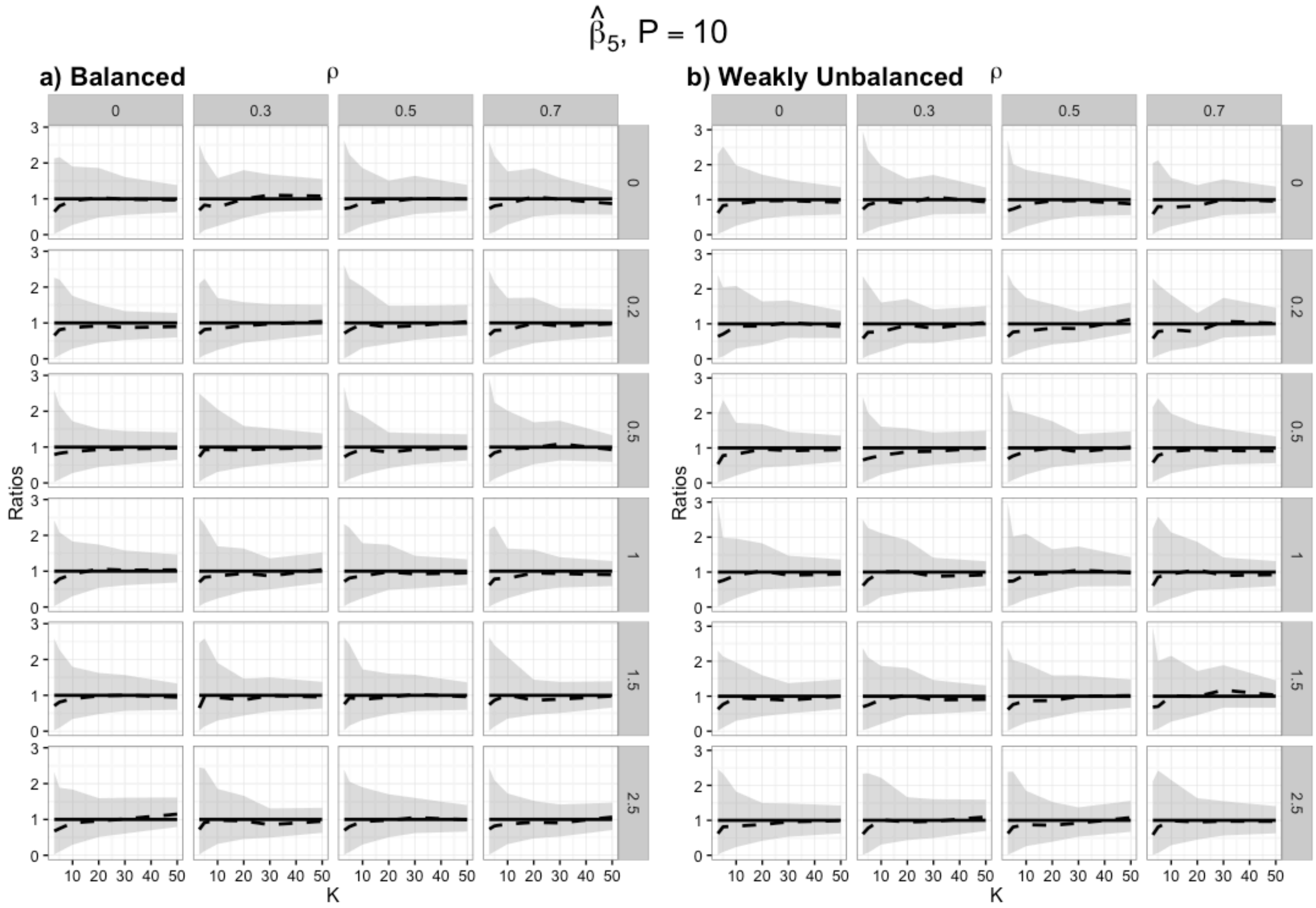
$\hat{\beta}_5$, P = 10
a) Balanced
b) Weakly Unbalanced
ρ
0
0.3
0.5
0.7
0
0.2
0.5
1
1.5
2.5
Ratios
0
1
2
3
10 20 30 40 50
K

13

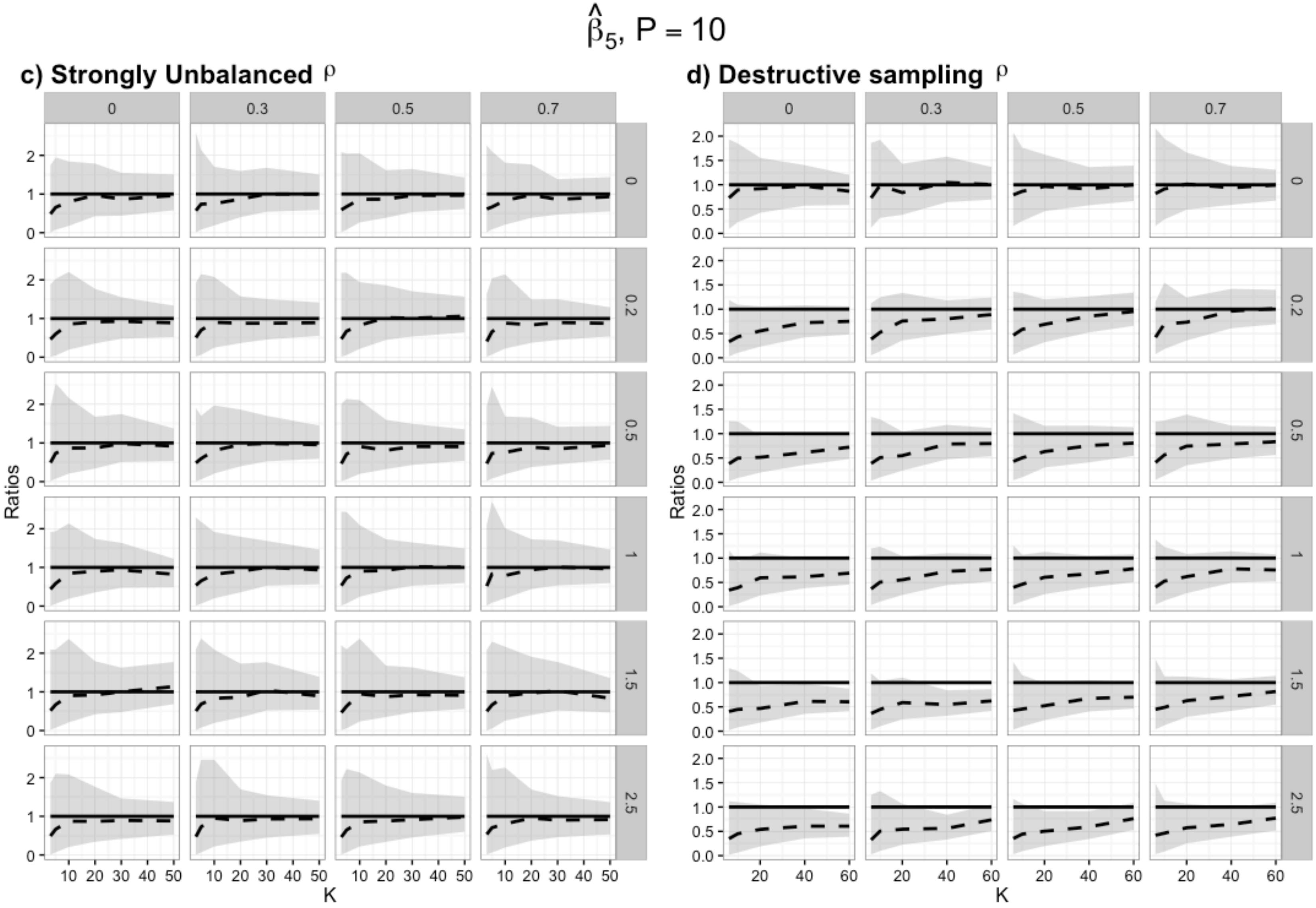

$\hat{\beta}_5$, P = 10
c) Strongly Unbalanced ρ
d) Destructive sampling ρ
0
0.3
0.5
0.7
0.2
1
1.5
2.5
Ratios
K

14

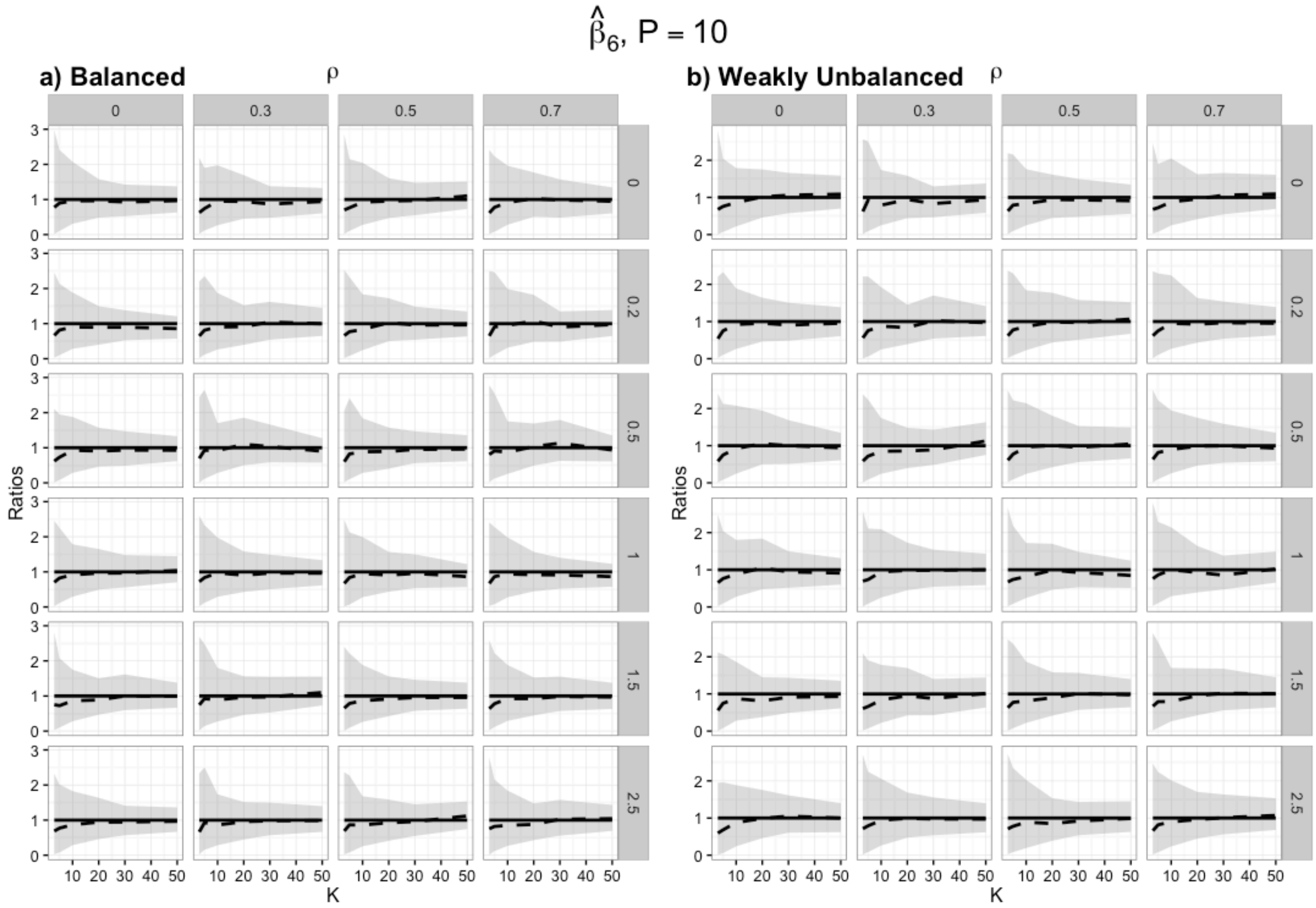
$\hat{\beta}_6$, P = 10
a) Balanced
b) Weakly Unbalanced
ρ
0
0.3
0.5
0.7
0
0.2
0.5
1
1.5
2.5
Ratios
K
10 20 30 40 50

15

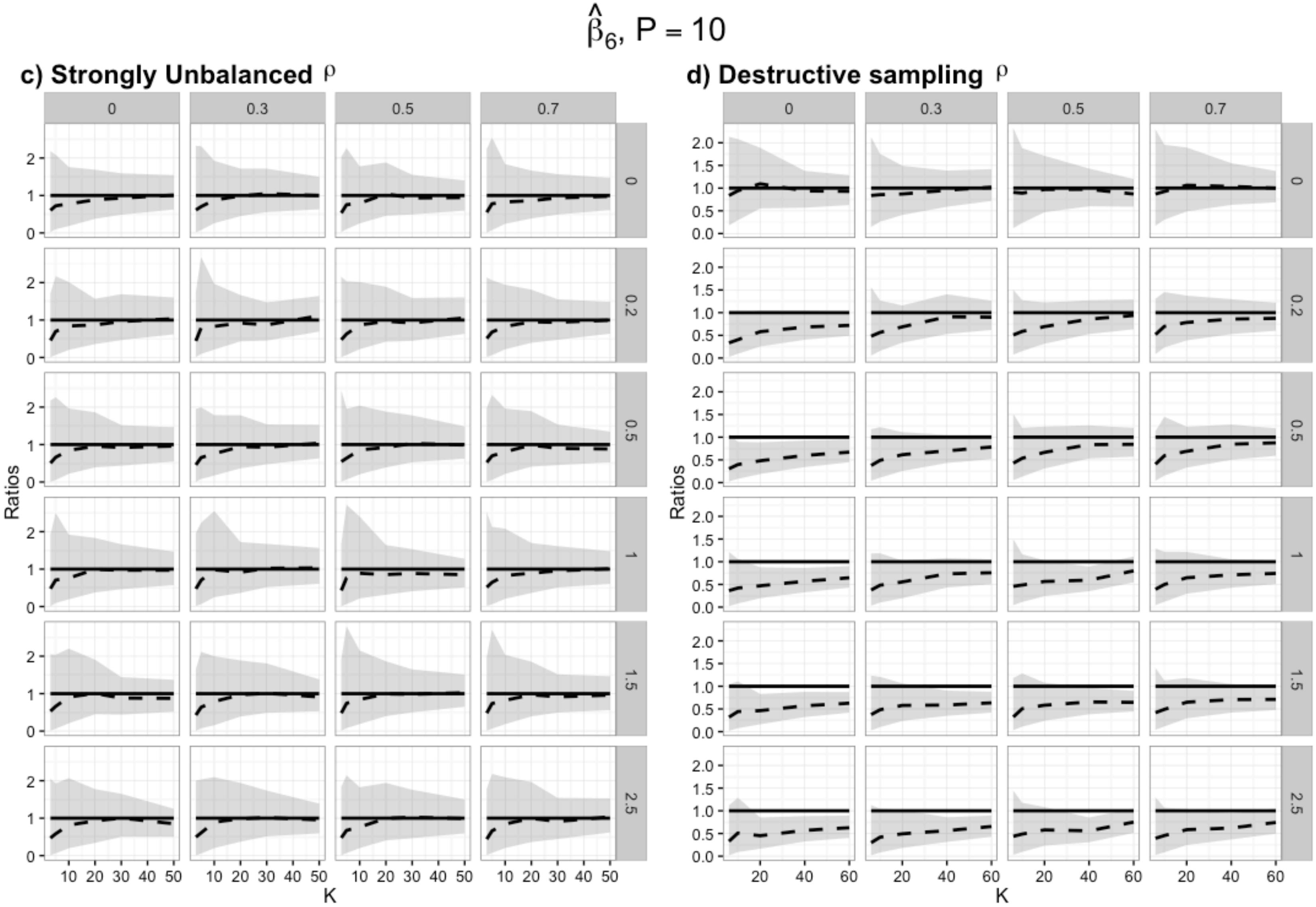
$\hat{\beta}_6$, P = 10
c) Strongly Unbalanced ρ
d) Destructive sampling ρ
0
0.3
0.5
0.7
0
0.2
0.5
1
1.5
2.5
Ratios
K
10 20 30 40 50
20 40 60

16

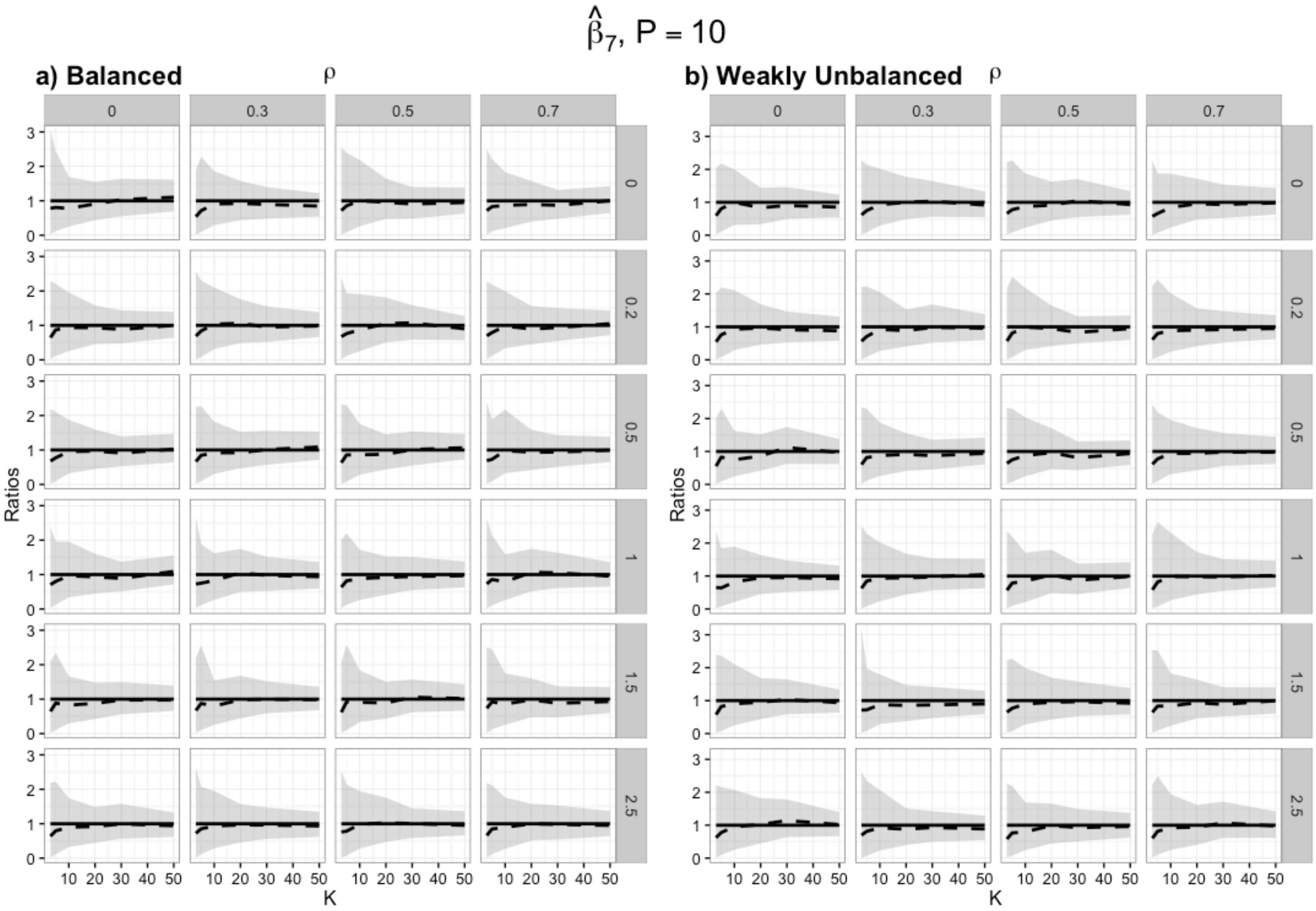

$\hat{\beta}_7$, P = 10
a) Balanced
b) Weakly Unbalanced
ρ
0
0.3
0.5
0.7
0
0.2
0.5
1
1.5
2.5
Ratios
K
10 20 30 40 50
0 1 2 3

17

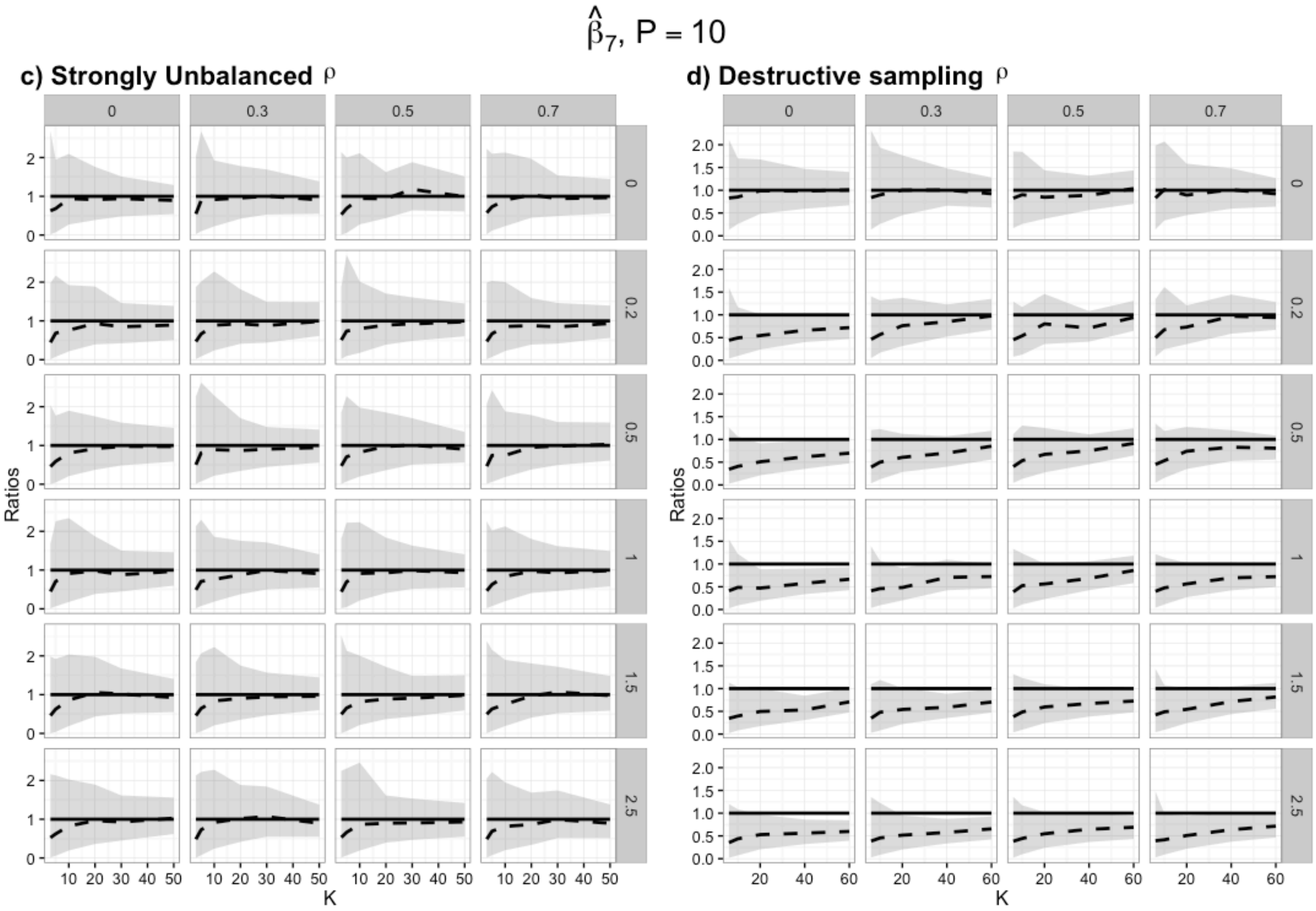
$\hat{\beta}_7$, P = 10
c) Strongly Unbalanced ρ
d) Destructive sampling ρ
0
0.3
0.5
0.7
0.2
1
1.5
2.5
Ratios
K
10 20 30 40 50
20 40 60

18

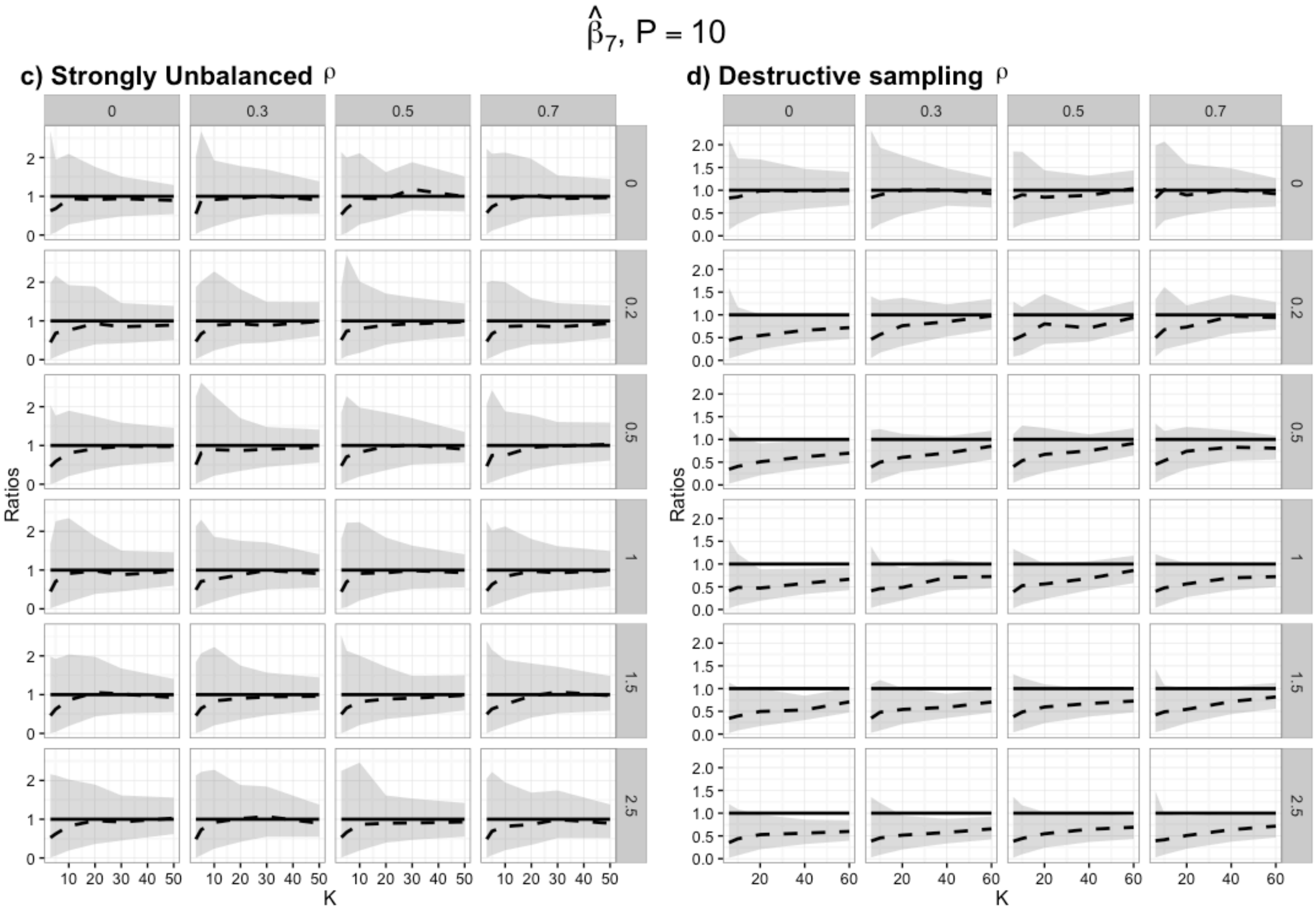
$\hat{\beta}_7$, P = 10
c) Strongly Unbalanced ρ
d) Destructive sampling ρ
0
0.3
0.5
0.7
0.2
1
1.5
2.5
Ratios
K

19

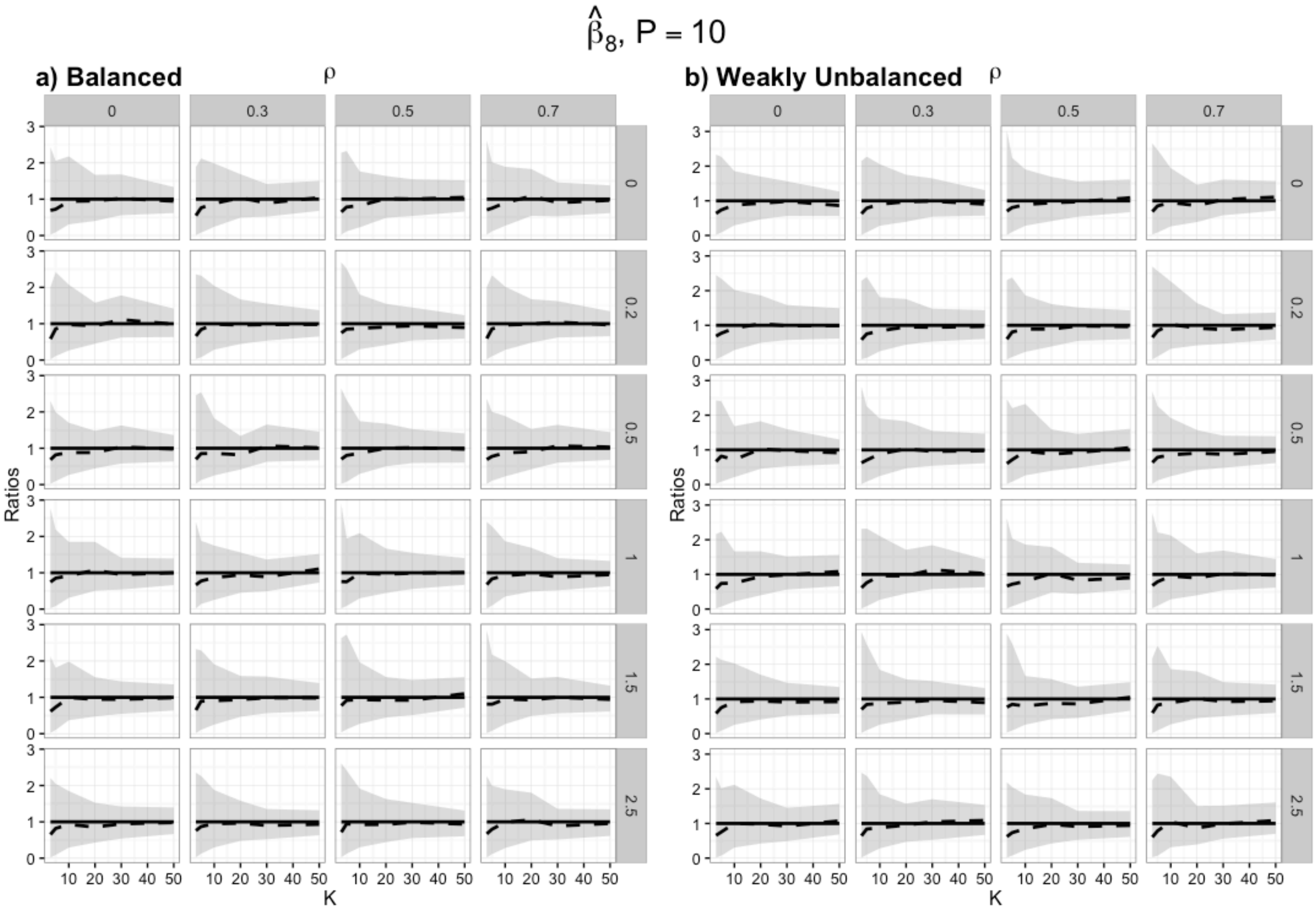

$\hat{\beta}_8$, P = 10
a) Balanced
b) Weakly Unbalanced
ρ
0
0.3
0.5
0.7
0
0.2
0.5
1
1.5
2.5
Ratios
K
10 20 30 40 50

20

$\hat{\beta}_8$, P = 10

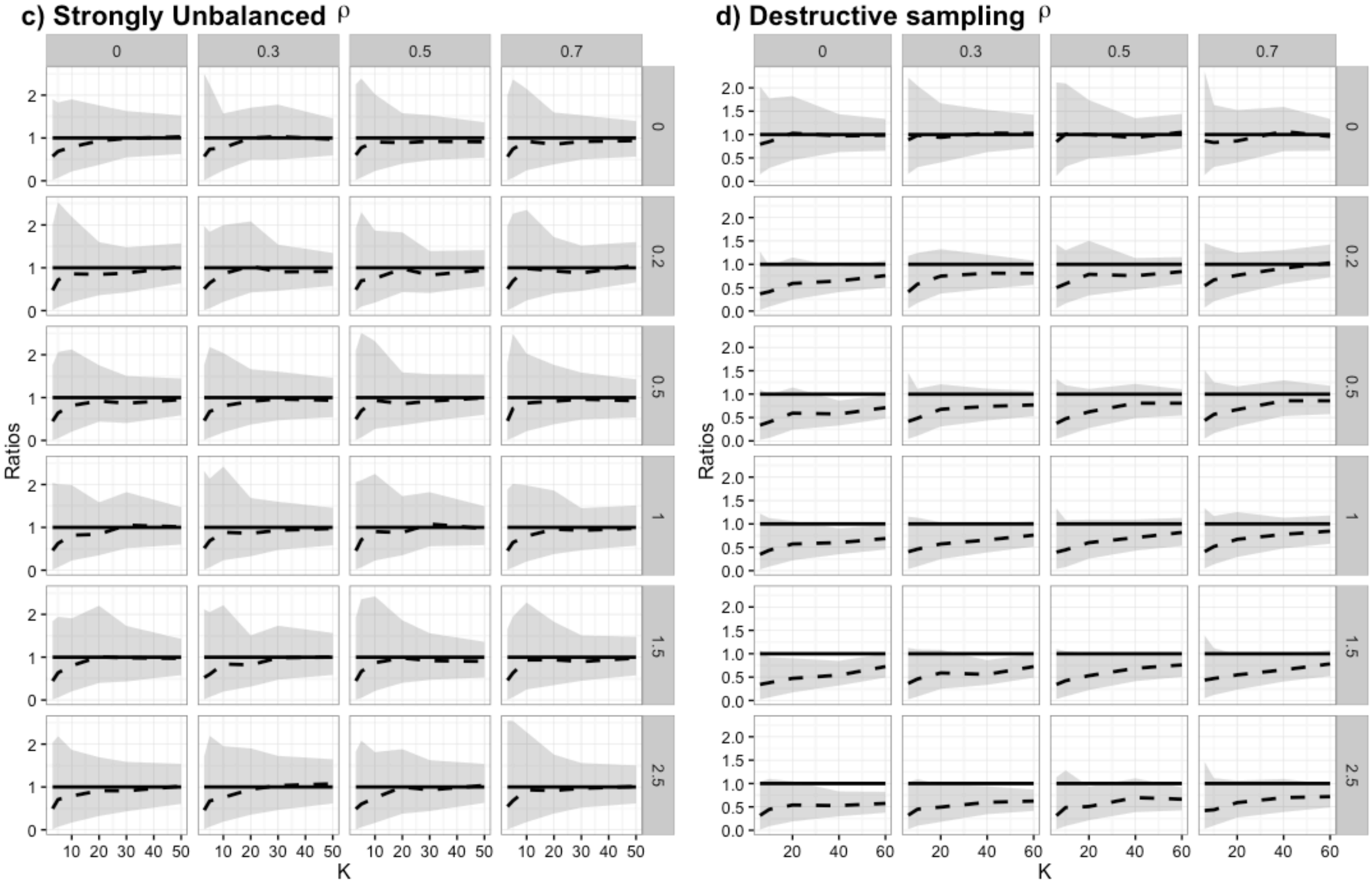

21

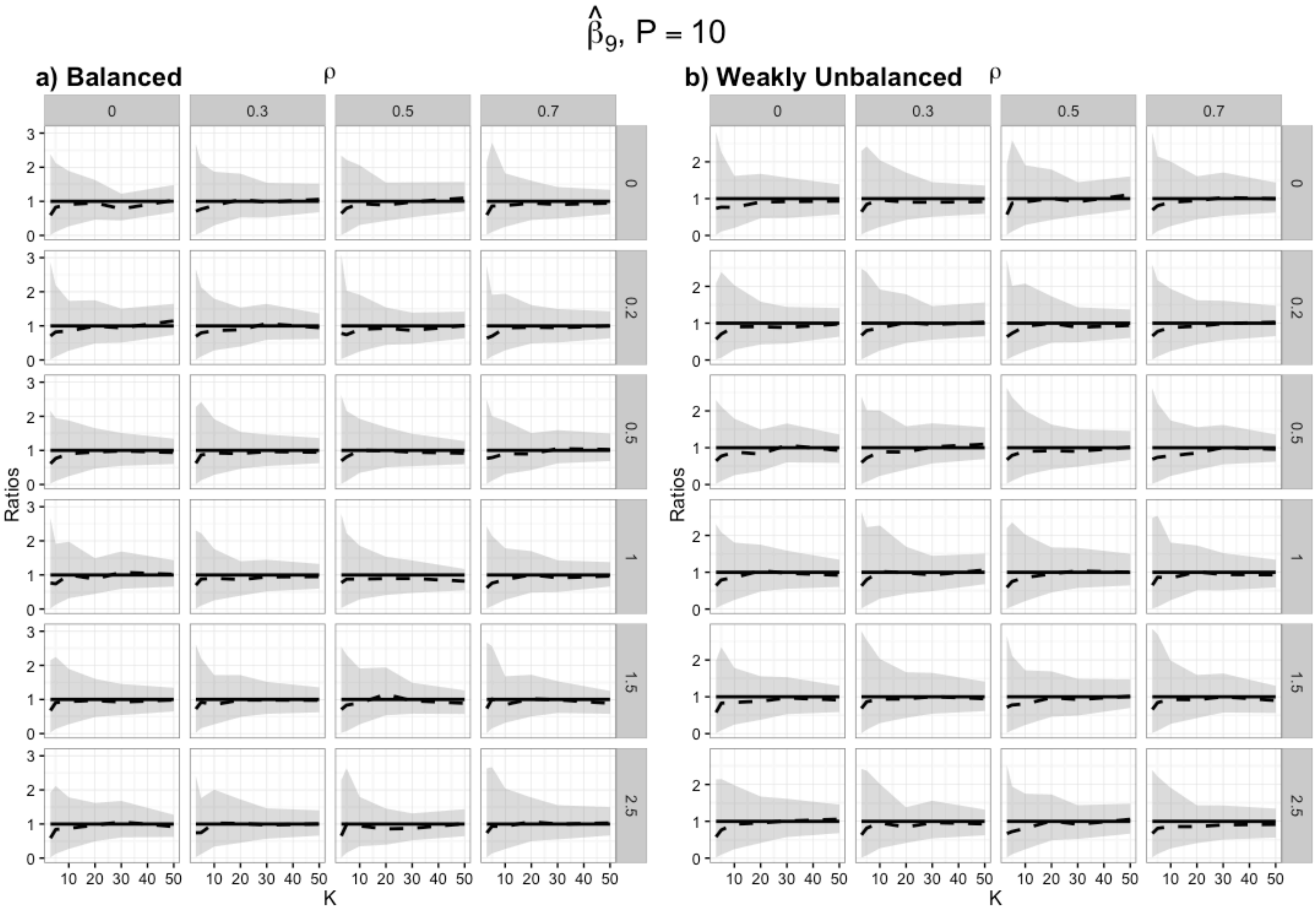

$\hat{\beta}_9$, P = 10
a) Balanced
b) Weakly Unbalanced
ρ
0
0.3
0.5
0.7
0
0.2
0.5
1
1.5
2.5
Ratios
K
10 20 30 40 50

22

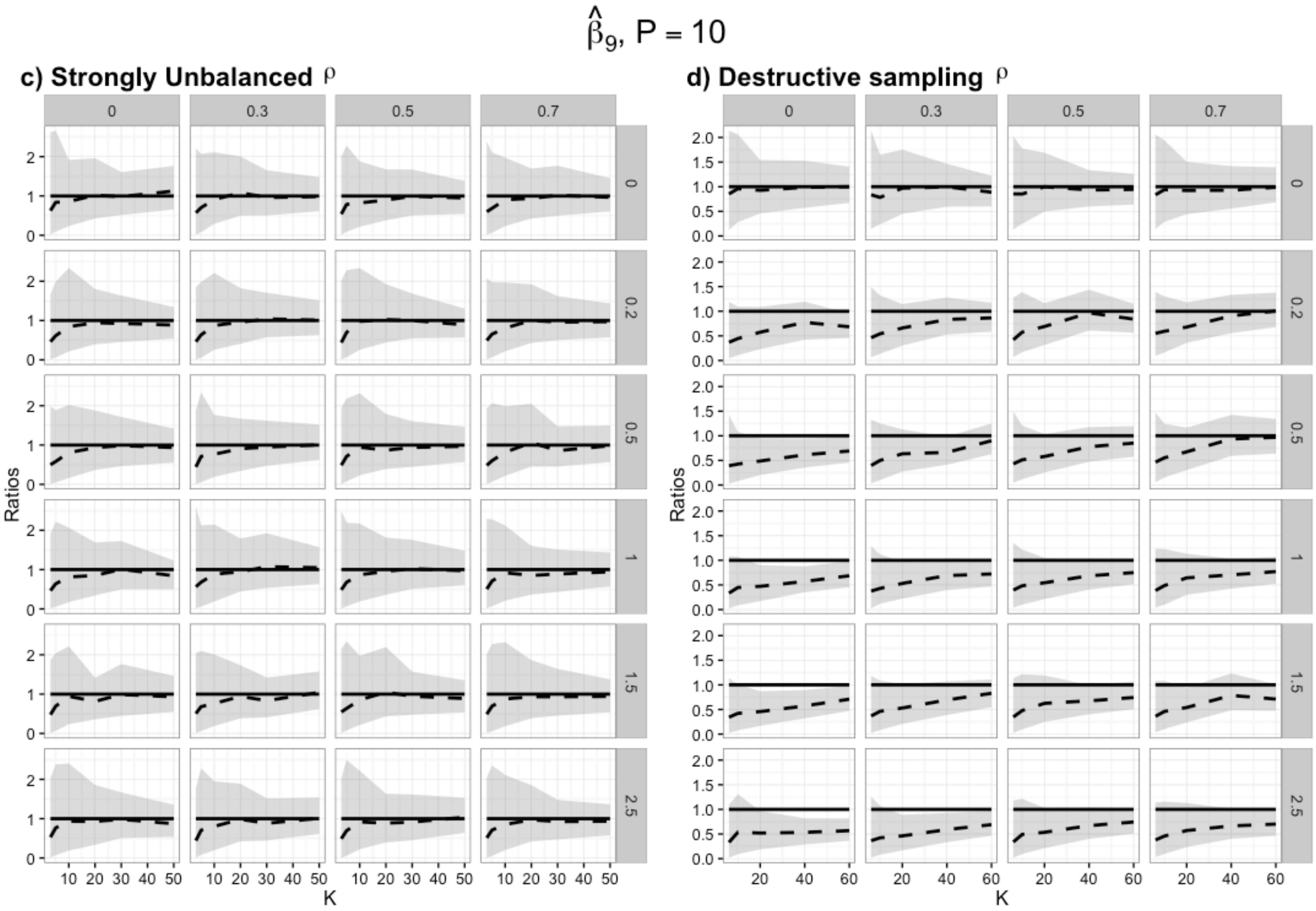

$\hat{\beta}_9$, P = 10
c) Strongly Unbalanced ρ
d) Destructive sampling ρ
0
0.3
0.5
0.7
0.2
1
1.5
2.5
Ratios
K

23

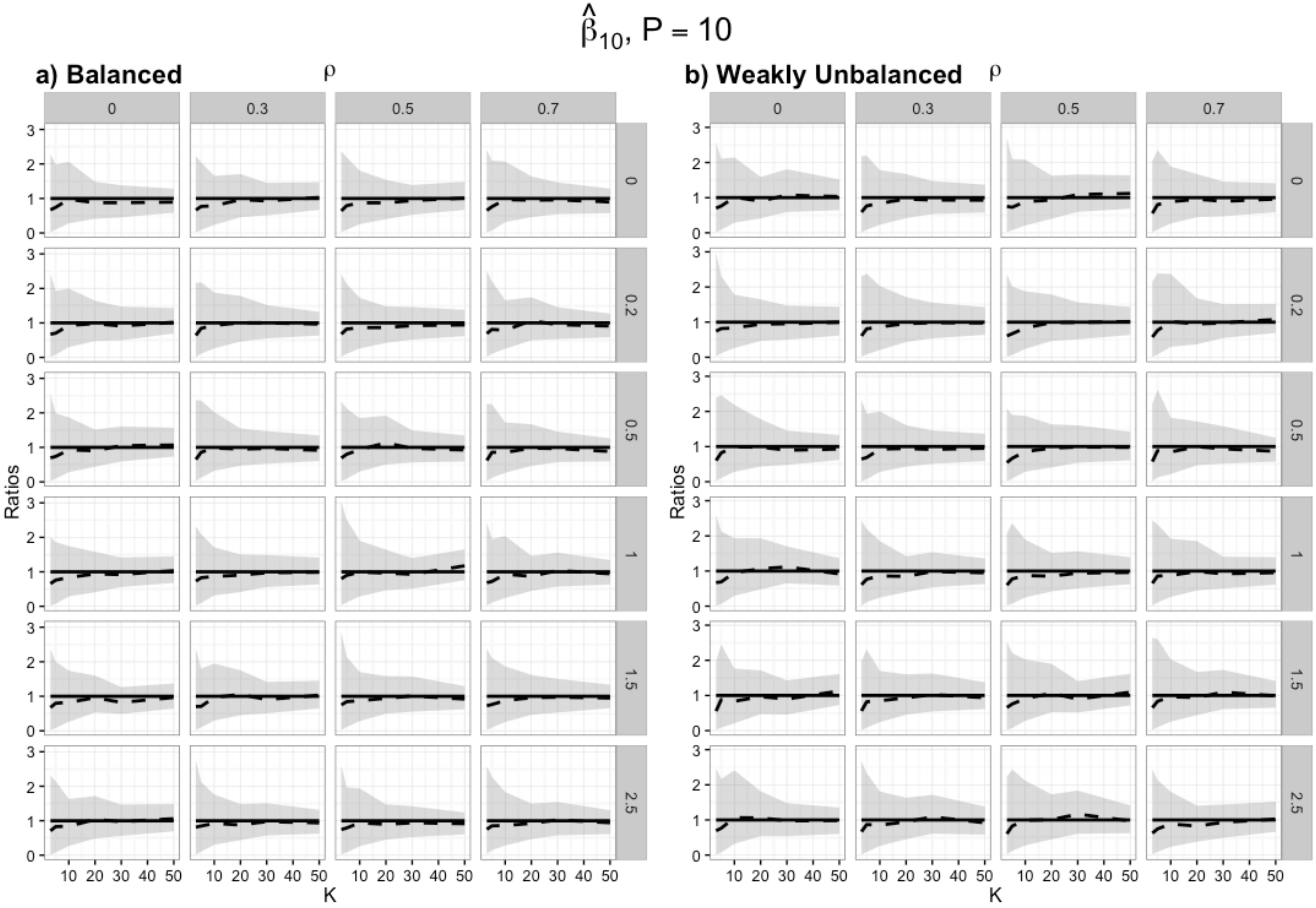
$\hat{\beta}_{10}$, P = 10
a) Balanced
b) Weakly Unbalanced
ρ
0
0.3
0.5
0.7
0.2
1
1.5
2.5
Ratios
K
10 20 30 40 50

24

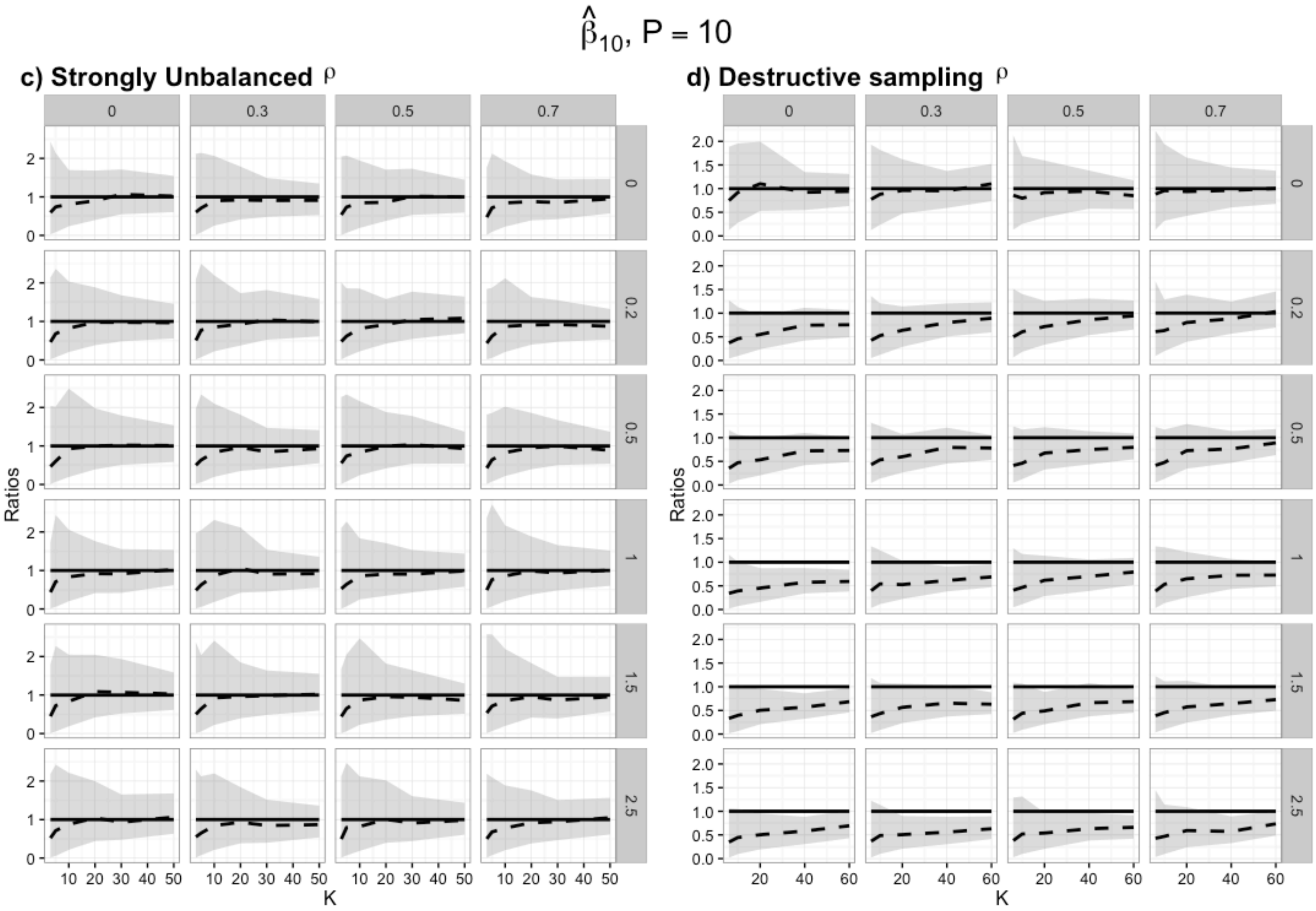
$\hat{\beta}_{10}$, P = 10
c) Strongly Unbalanced ρ
d) Destructive sampling ρ
0
0.3
0.5
0.7
0.2
1
1.5
2.5
Ratios
K

25
