# Supplementary material for: Robust Inference from Conditional Logistic Regression Applied to Movement and Habitat Selection Analysis
Source: PLoS One. 2017 Jan 12;12(1):e0169779. doi: 10.1371/journal.pone.0169779 (PMC5233429; doi:10.1371/journal.pone.0169779)
Supplement: S2 Fig — Comparison of average ratios between robust estimates of variance (VR/VT, dashed lines) or naive estimates of variance over true variance (VN/VT, dotted lines) of coefficients β^p, for different number of covariates (P) and different number of clusters (K), as a function of temporal autocorrelation (ρ) and inter-individual heterogeneity (σH2 on the left side of the panels) as well as different data processing: a) Balanced, b) Weakly Unbalanced, c) Strongly Unbalanced and d) Destructive sampling. Robust or naive estimates of variance are unbiased when ratios are not significantly different from 1 (solid line). (PDF) [file pone.0169779.s003.pdf]

$\hat{\beta}_1$, P = 2

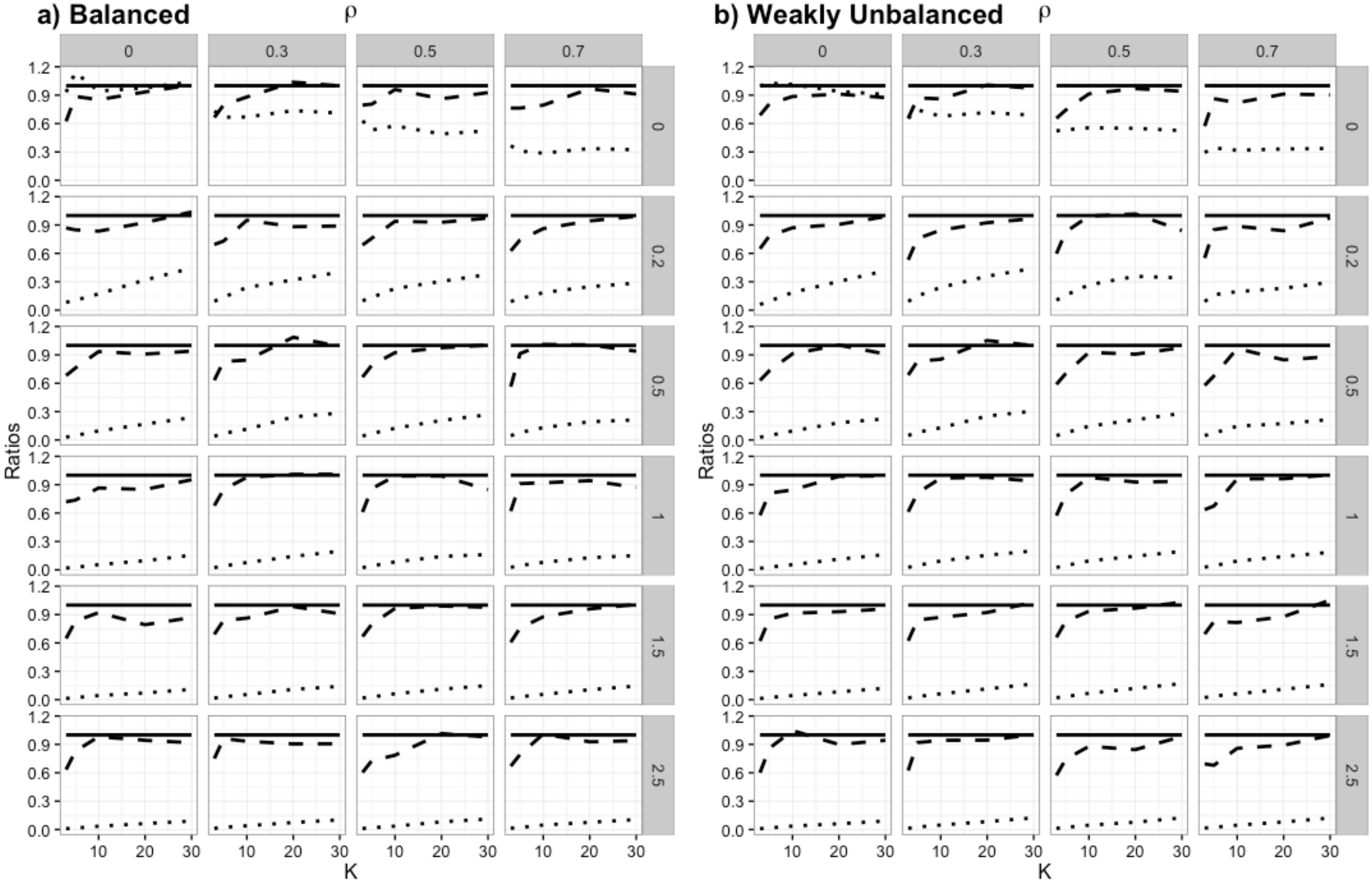

a) Balanced
b) Weakly Unbalanced
ρ
0
0.3
0.5
0.7
0
0.2
0.5
1
1.5
2.5
Ratios
K

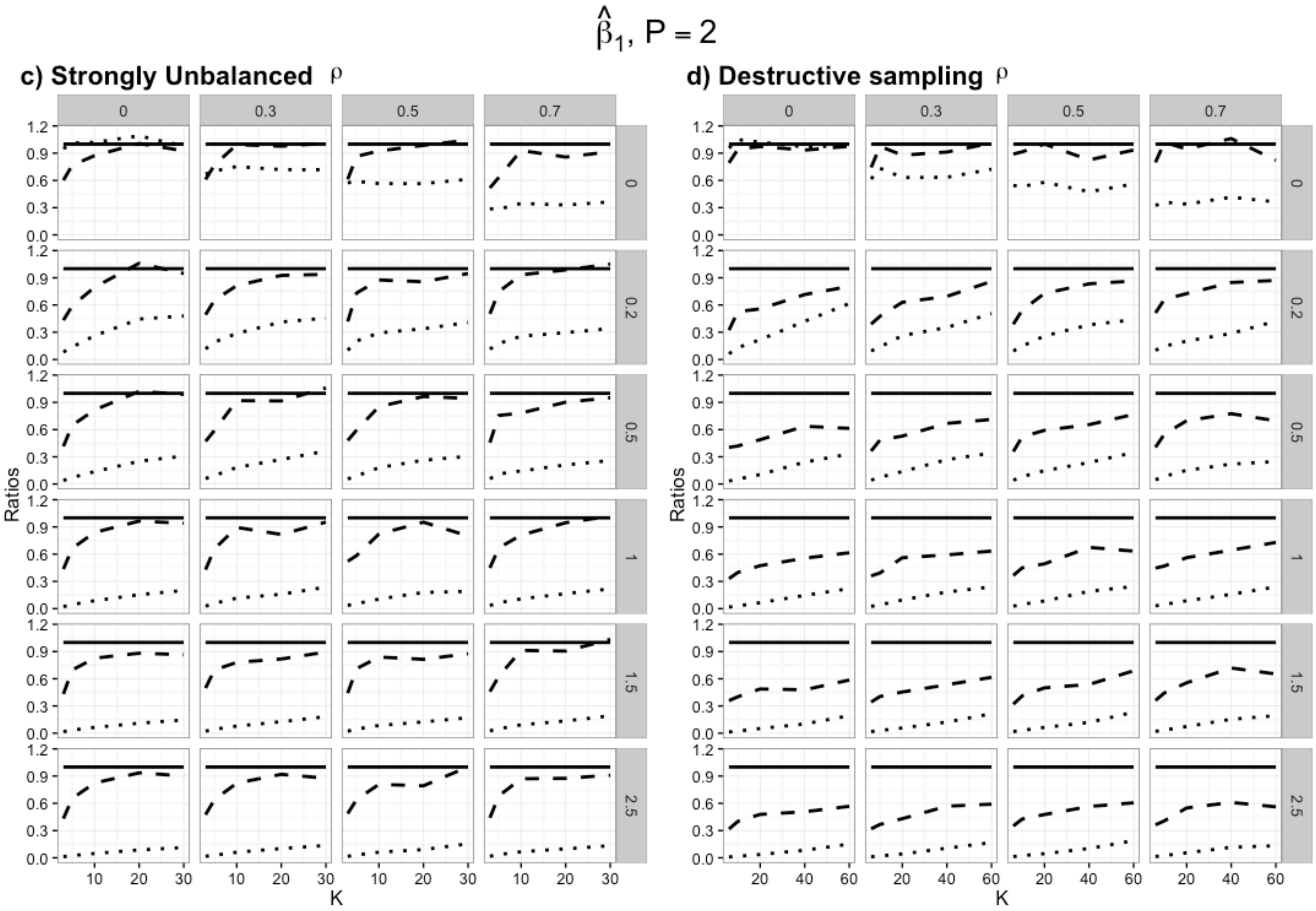
$\hat{\beta}_1$, P = 2
c) Strongly Unbalanced ρ
d) Destructive sampling ρ
0
0.3
0.5
0.7
0.2
1
1.5
2.5
Ratios
K
1.2
0.9
0.6
0.3
0.0
10
20
30
40
60

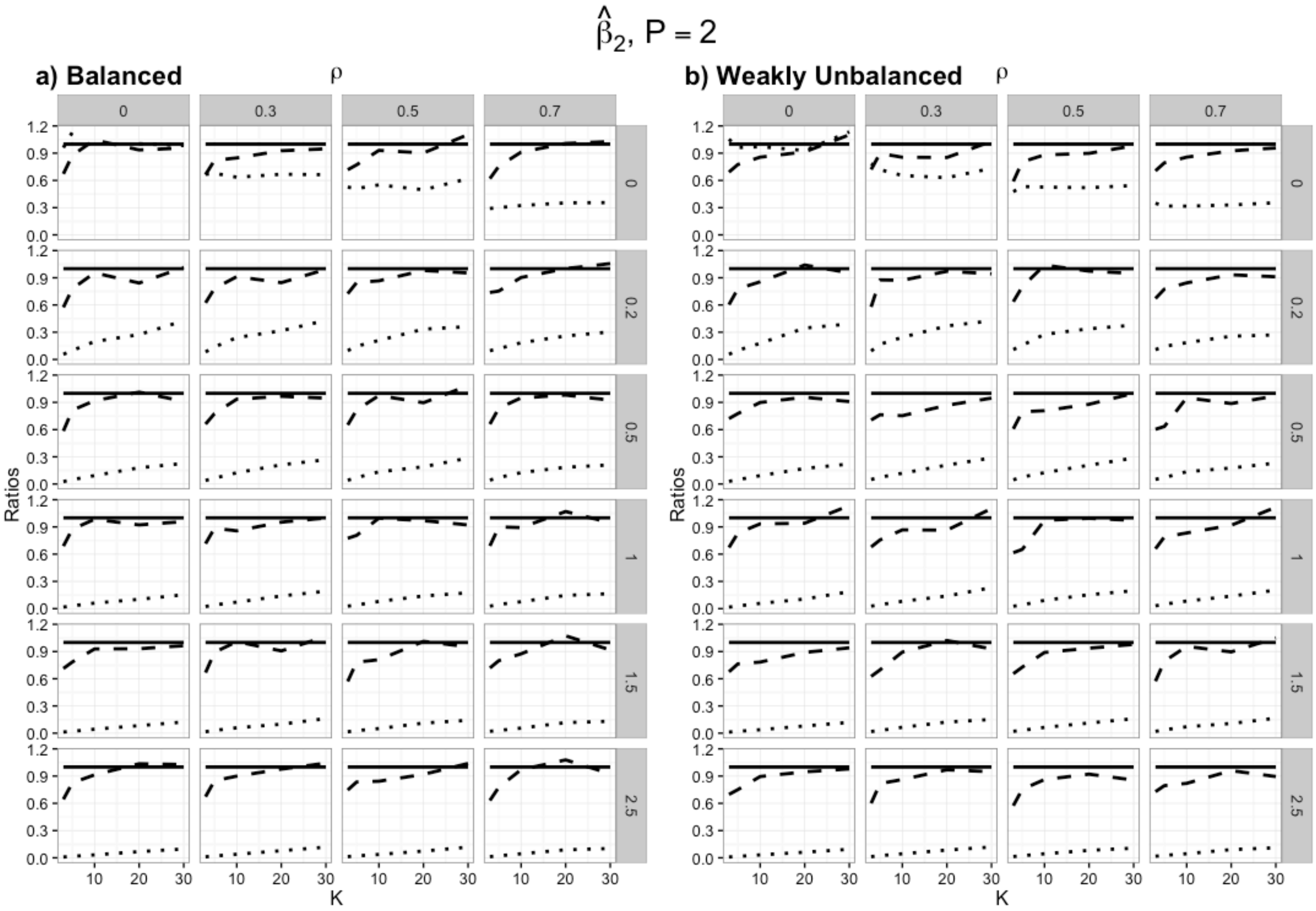
$\hat{\beta}_2$, P = 2
a) Balanced
b) Weakly Unbalanced
ρ
0
0.3
0.5
0.7
0
0.2
0.5
1
1.5
2.5
Ratios
K
10
20
30
0.0
0.3
0.6
0.9
1.2

$\hat{\beta}_2$, P = 2

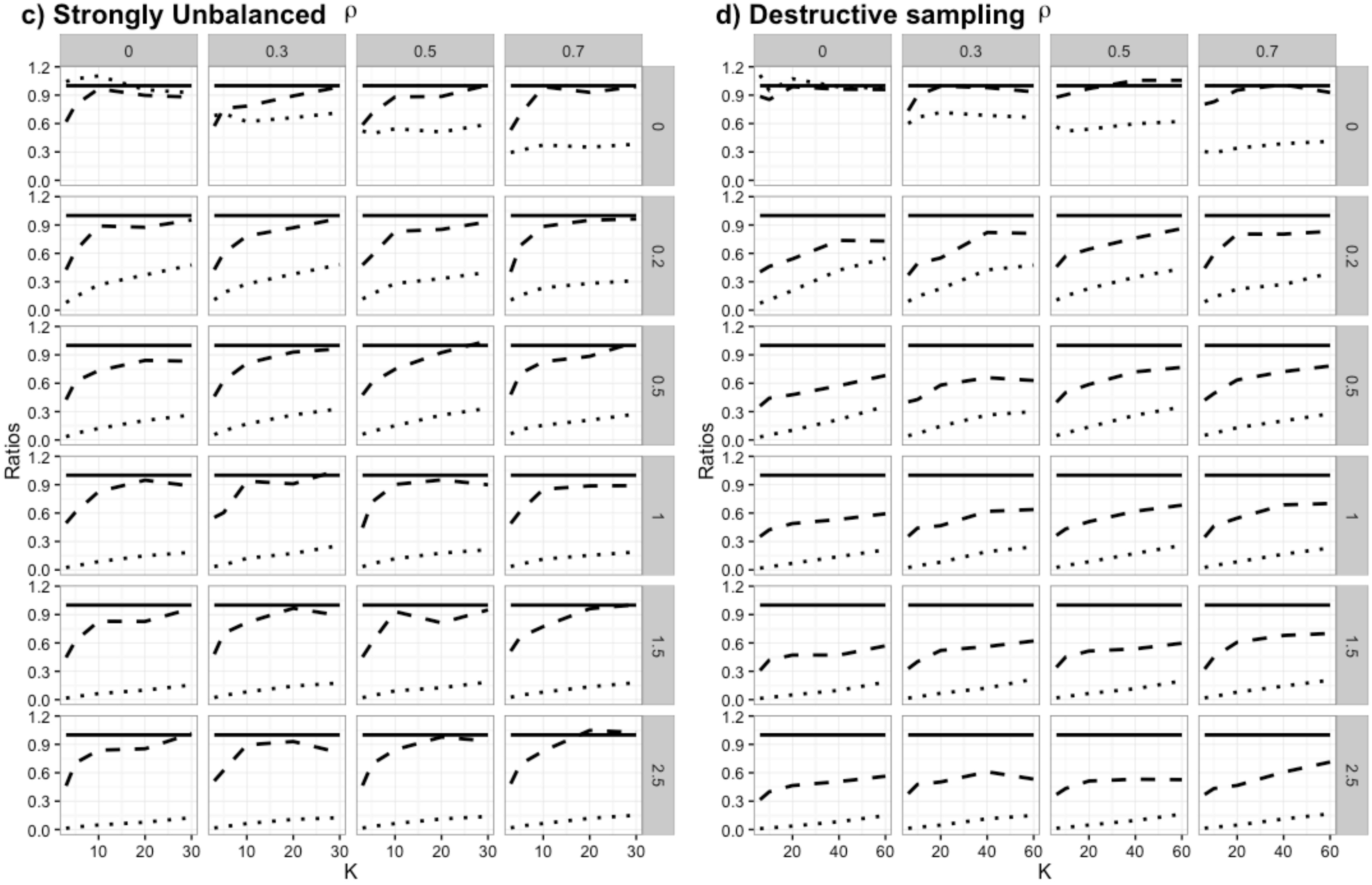
c) Strongly Unbalanced
ρ
0
0.3
0.5
0.7
0
0.2
0.5
1
1.5
2.5
Ratios
K
d) Destructive sampling
ρ
0
0.3
0.5
0.7
0
0.2
0.5
1
1.5
2.5
Ratios
K

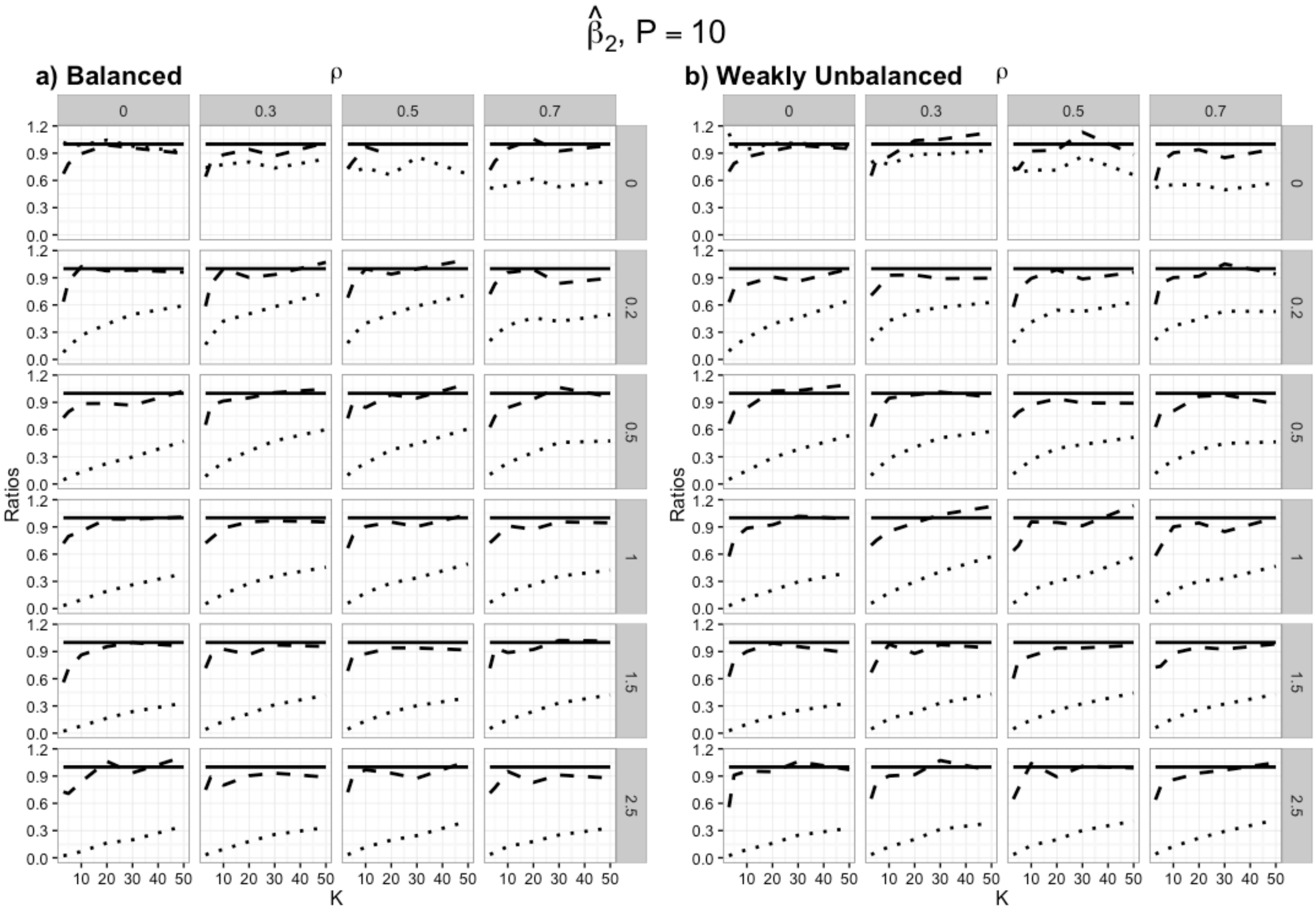

$\hat{\beta}_2$, P = 10
a) Balanced
b) Weakly Unbalanced
ρ
0
0.3
0.5
0.7
0.2
1
1.5
2.5
Ratios
K
1.2
0.9
0.6
0.3
0.0
10 20 30 40 50

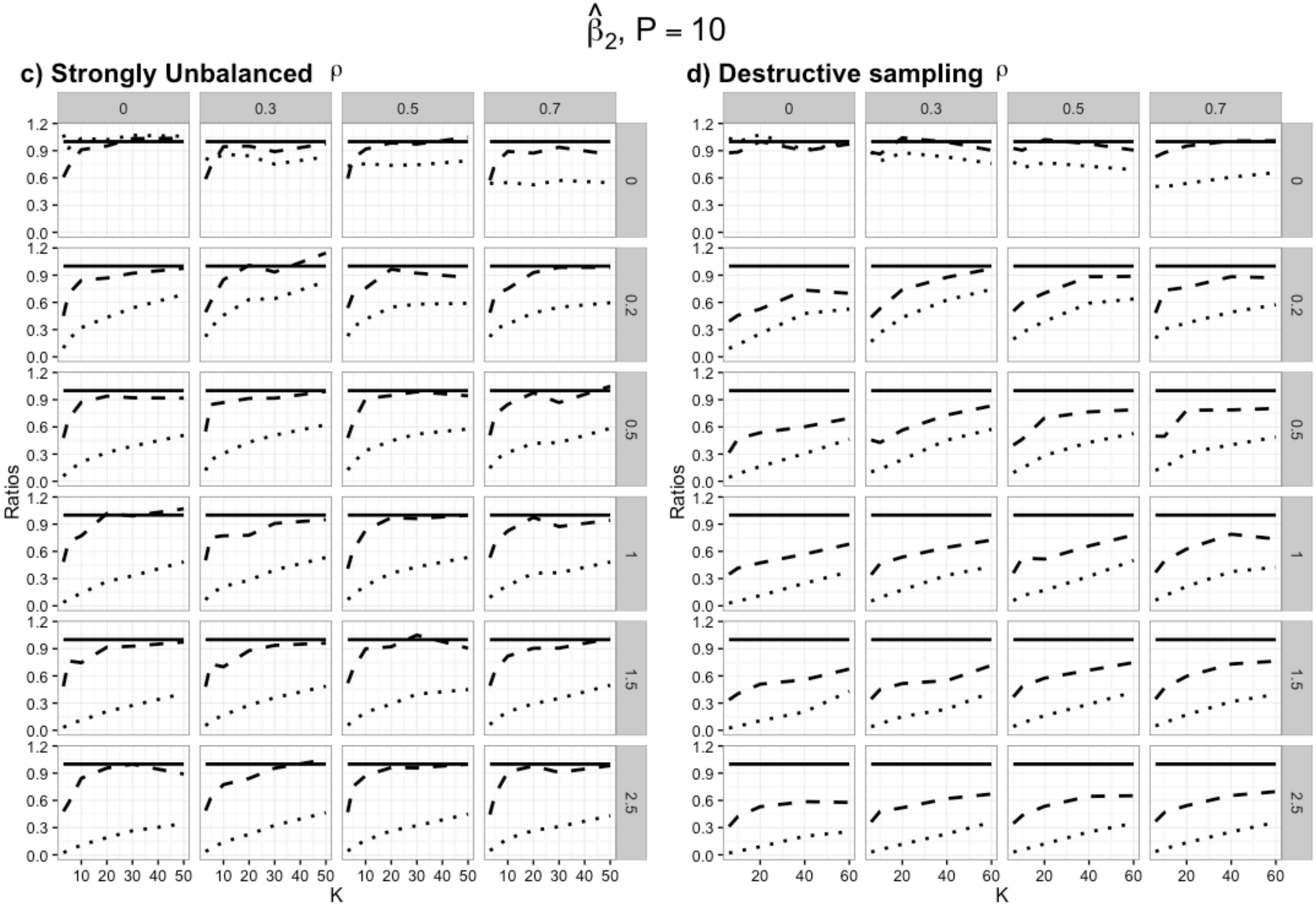

$\hat{\beta}_2$, P = 10
c) Strongly Unbalanced
d) Destructive sampling
ρ
0
0.3
0.5
0.7
0.2
1
1.5
2.5
1.2
0.9
0.6
0.0
Ratios
10
20
30
40
50
60
K

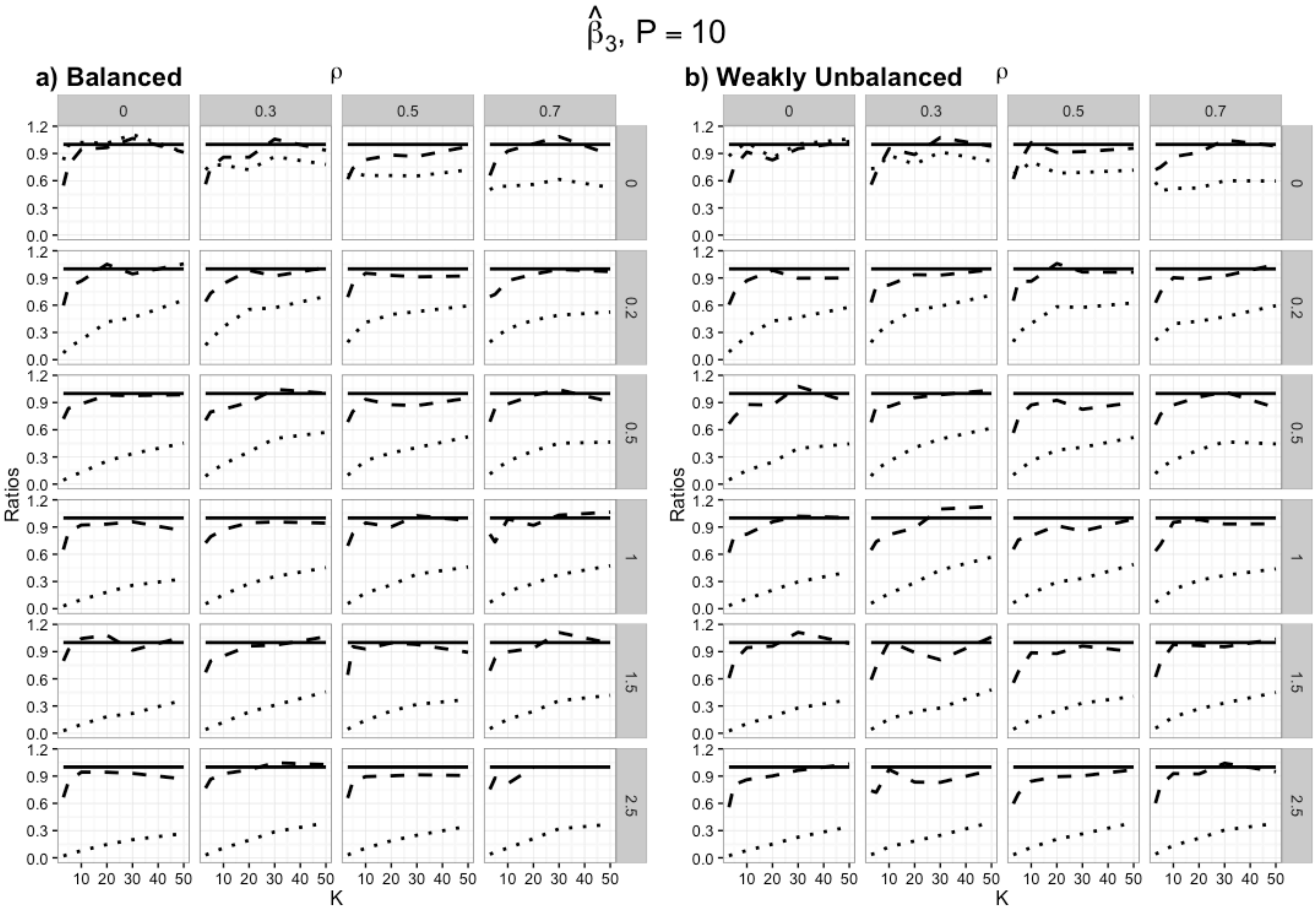

$\hat{\beta}_3$, P = 10
a) Balanced
b) Weakly Unbalanced
ρ
0
0.3
0.5
0.7
0
0.2
0.5
1
1.5
2.5
Ratios
K
1.2
0.9
0.6
0.3
0.0
10 20 30 40 50

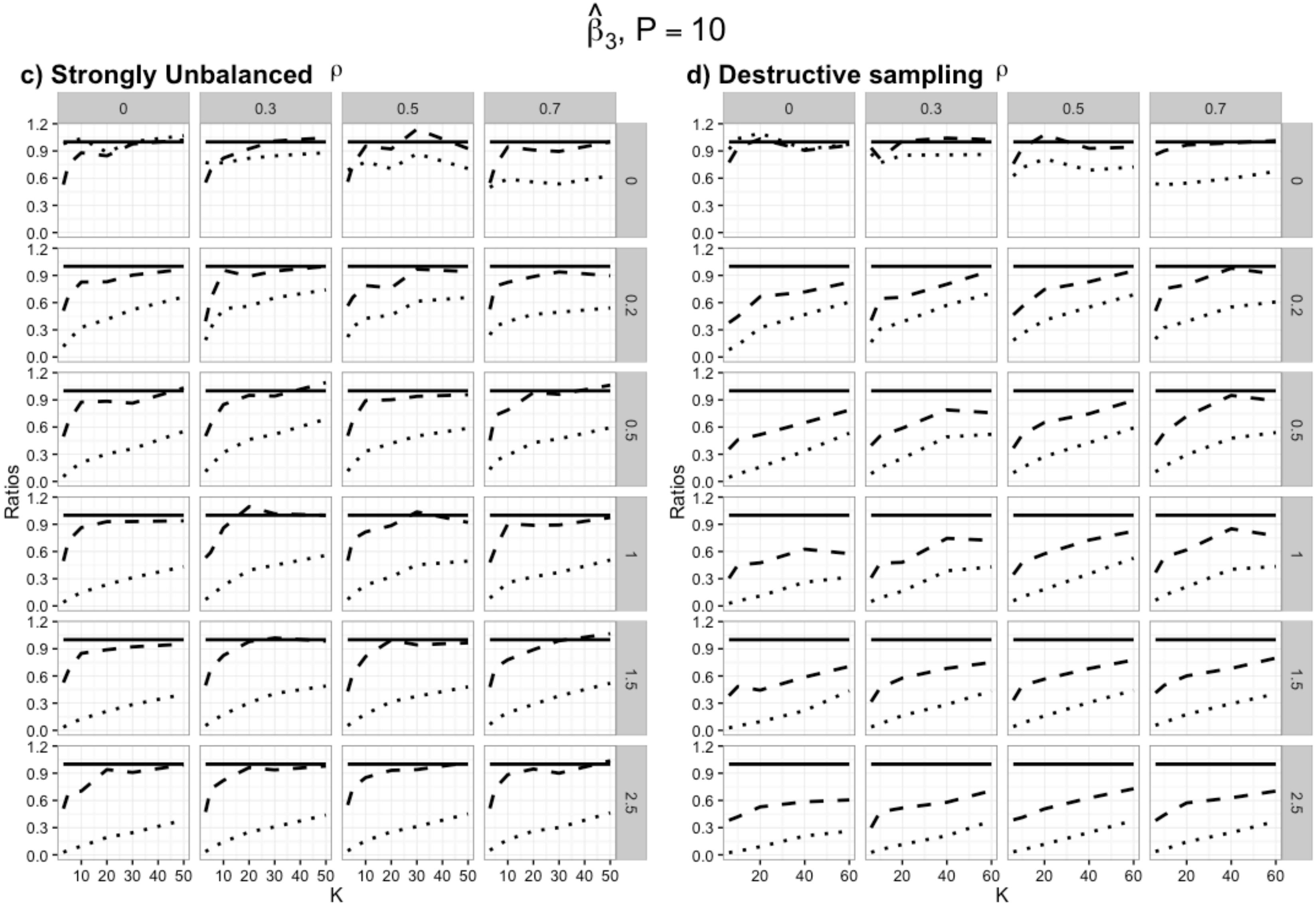
$\hat{\beta}_3$, P = 10
c) Strongly Unbalanced
d) Destructive sampling
ρ
0
0.3
0.5
0.7
0.2
1
1.5
2.5
Ratios
K
1.2
0.9
0.6
0.3
0.0
10
20
30
40
50
60

$\hat{\beta}_4$, P = 10

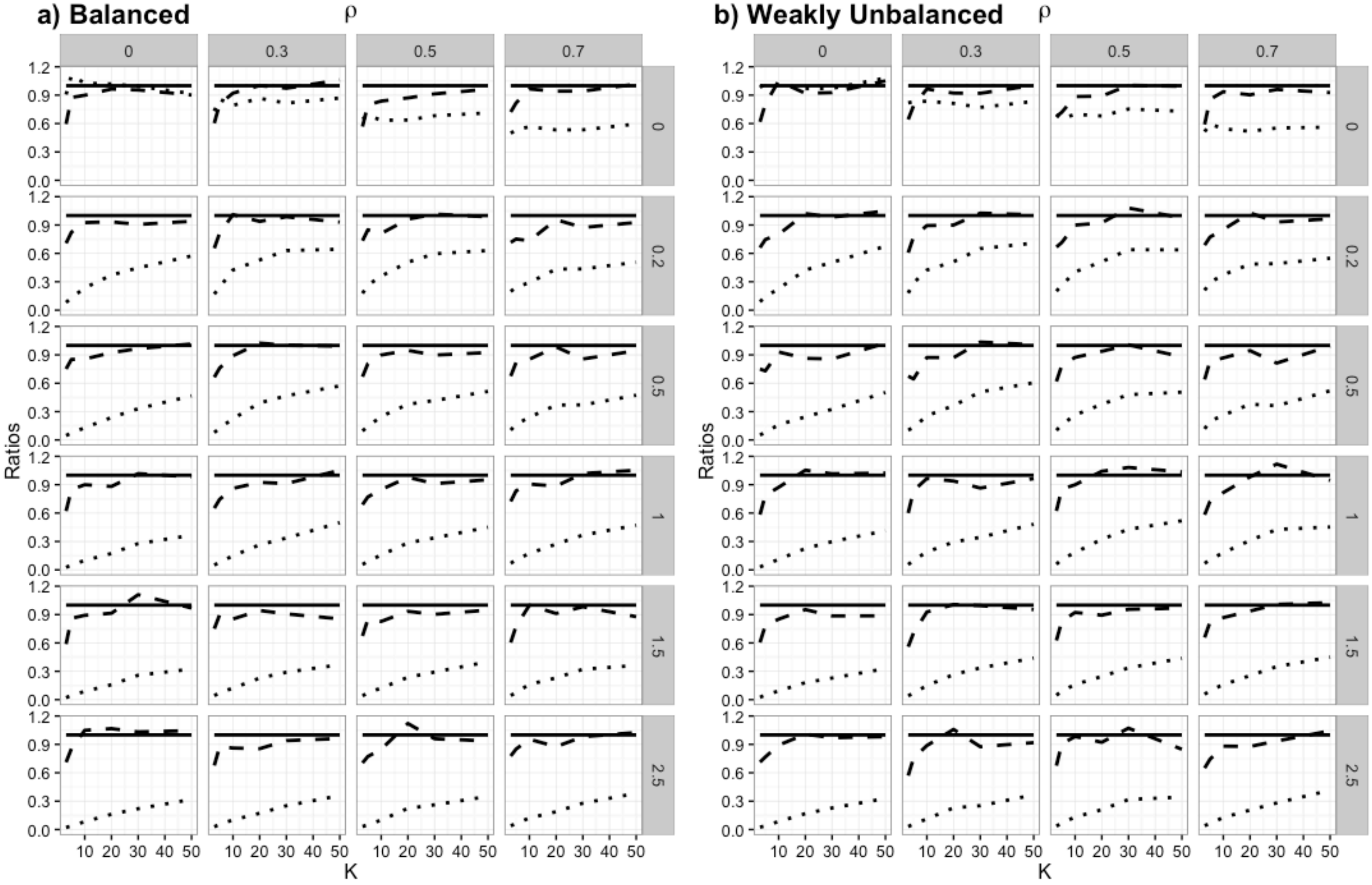

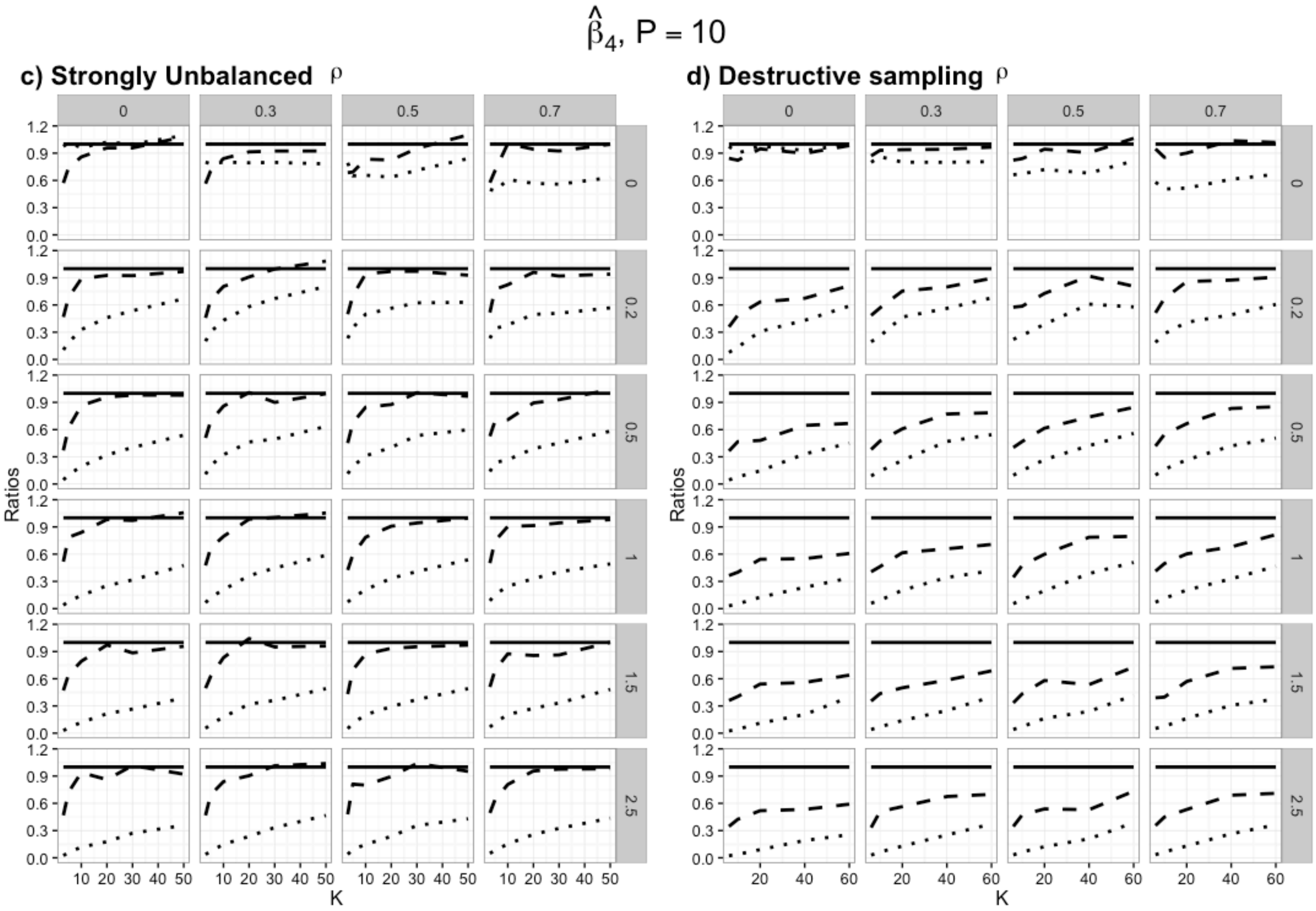

$\hat{\beta}_4$, P = 10
c) Strongly Unbalanced ρ
d) Destructive sampling ρ
0
0.3
0.5
0.7
0
0.2
0.5
1
1.5
2.5
Ratios
1.2
0.9
0.6
0.3
0.0
10 20 30 40 50
20 40 60
K

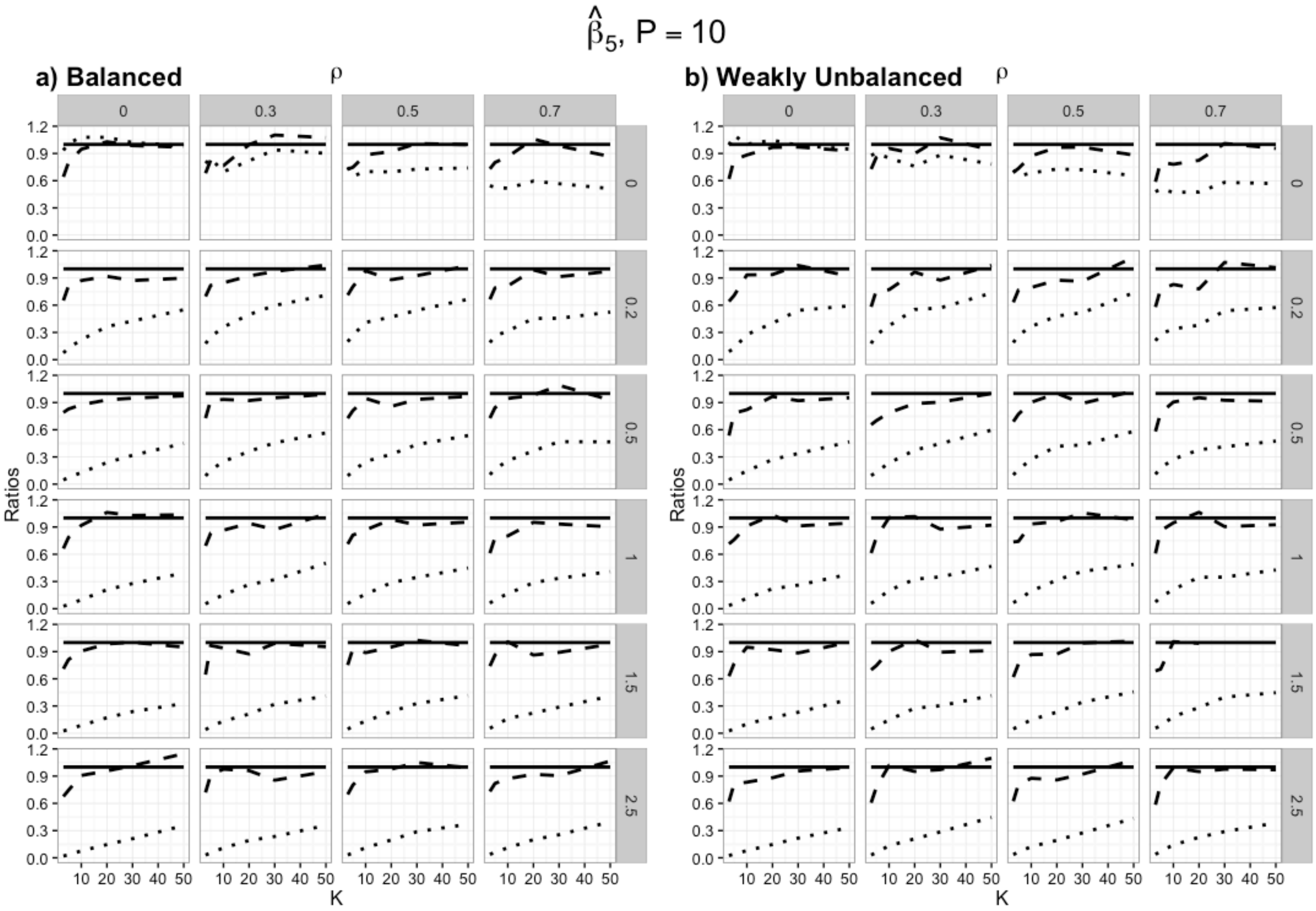

$\hat{\beta}_5$, P = 10
a) Balanced
b) Weakly Unbalanced
ρ
0
0.3
0.5
0.7
0.2
1
1.5
2.5
Ratios
K
0.0
0.3
0.6
0.9
1.2
10
20
30
40
50

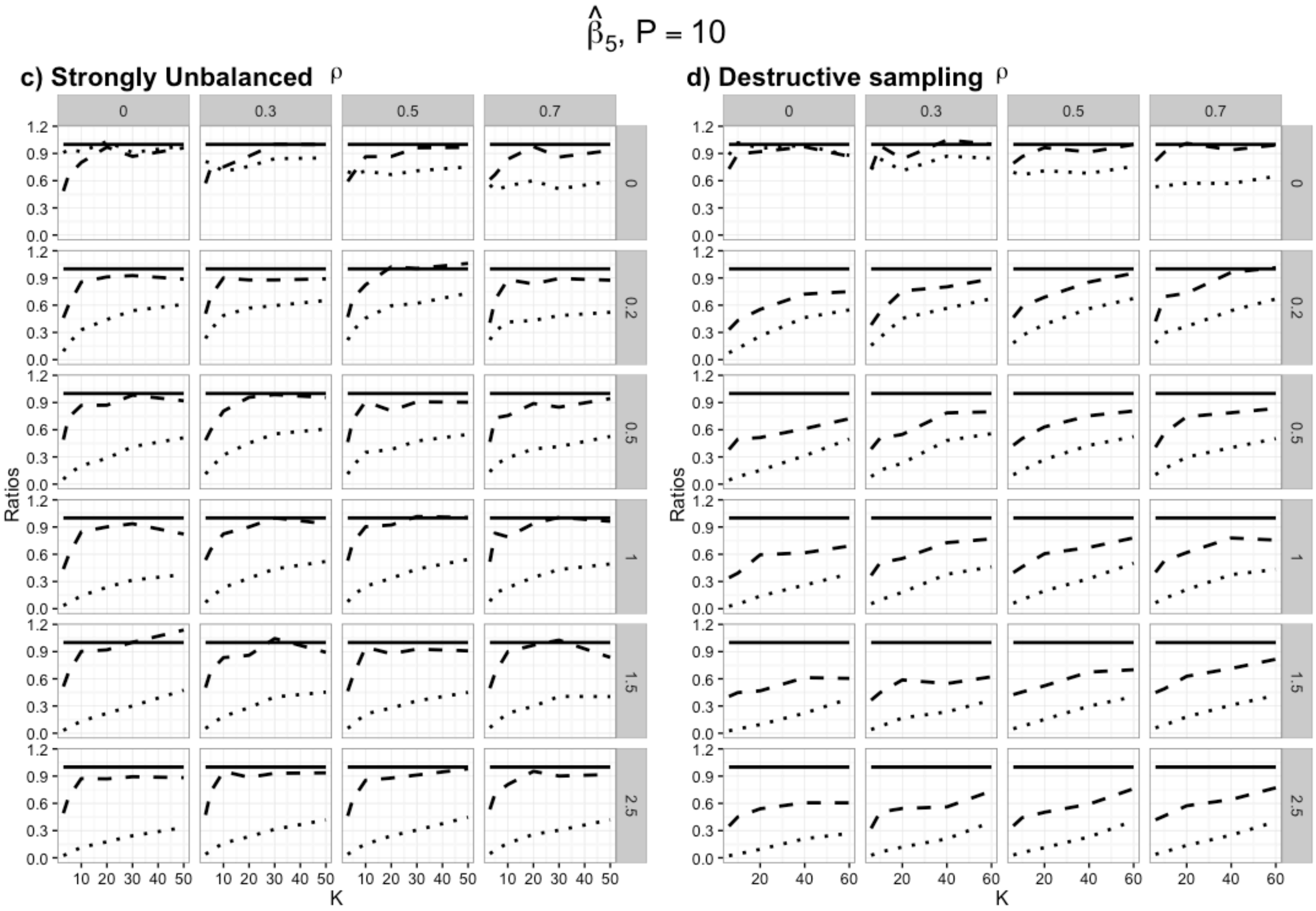

$\hat{\beta}_5$, P = 10
c) Strongly Unbalanced ρ
d) Destructive sampling ρ
0
0.3
0.5
0.7
0.2
1
1.5
2.5
Ratios
K
0.0
0.3
0.6
0.9
1.2
10
20
30
40
50
60

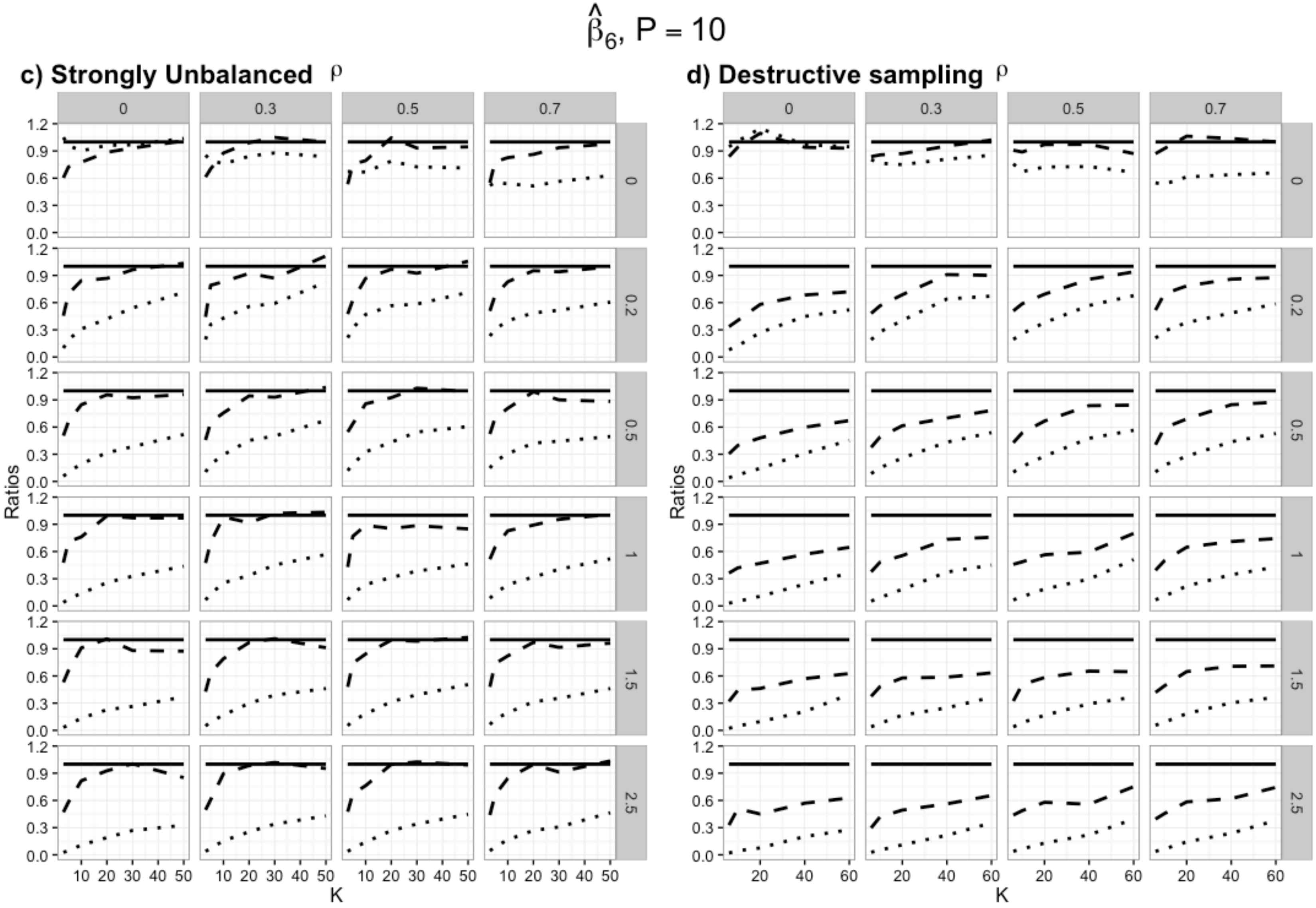

$\hat{\beta}_6$, P = 10
c) Strongly Unbalanced
d) Destructive sampling
ρ
0
0.3
0.5
0.7
0.2
1
1.5
2.5
Ratios
K

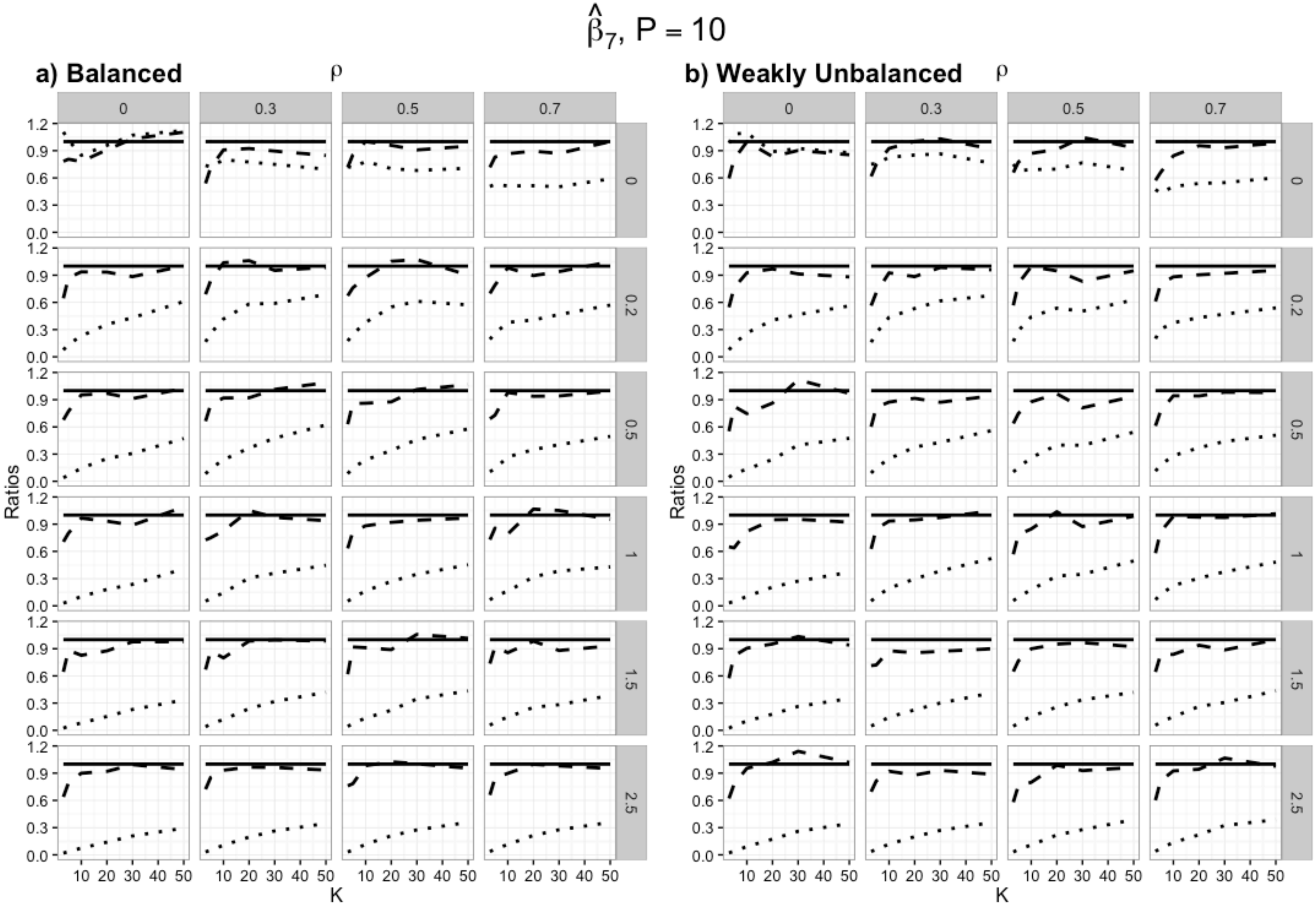
$\hat{\beta}_7$, P = 10
a) Balanced
b) Weakly Unbalanced
ρ
0
0.3
0.5
0.7
0.2
1
1.5
2.5
Ratios
K
1.2
0.9
0.6
0.3
0.0
10 20 30 40 50

$\hat{\beta}_7$, P = 10

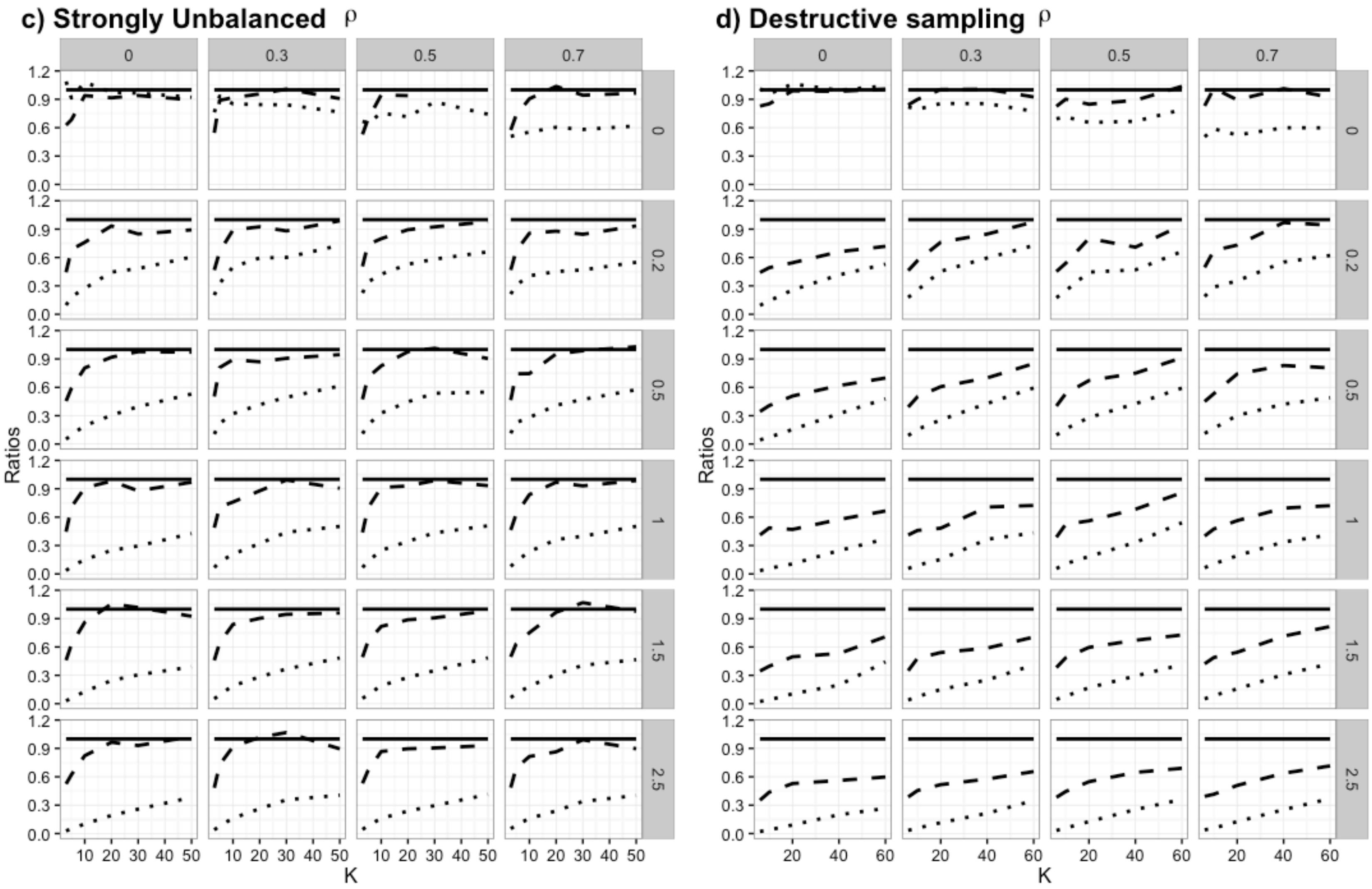

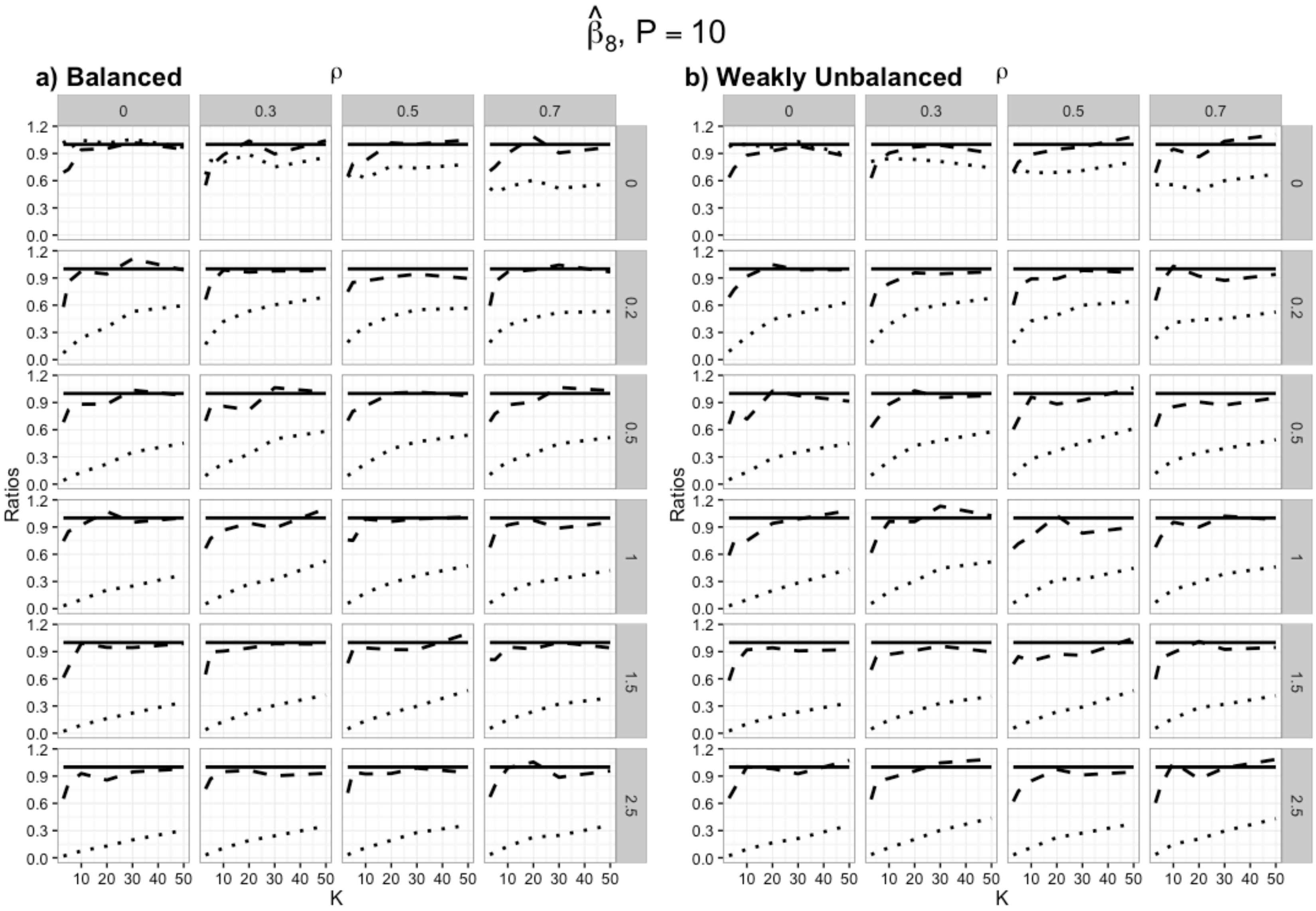
$\hat{\beta}_8$, P = 10
a) Balanced
b) Weakly Unbalanced
ρ
0
0.3
0.5
0.7
0.2
1
1.5
2.5
1.2
0.9
0.6
0.3
0.0
Ratios
10 20 30 40 50
K

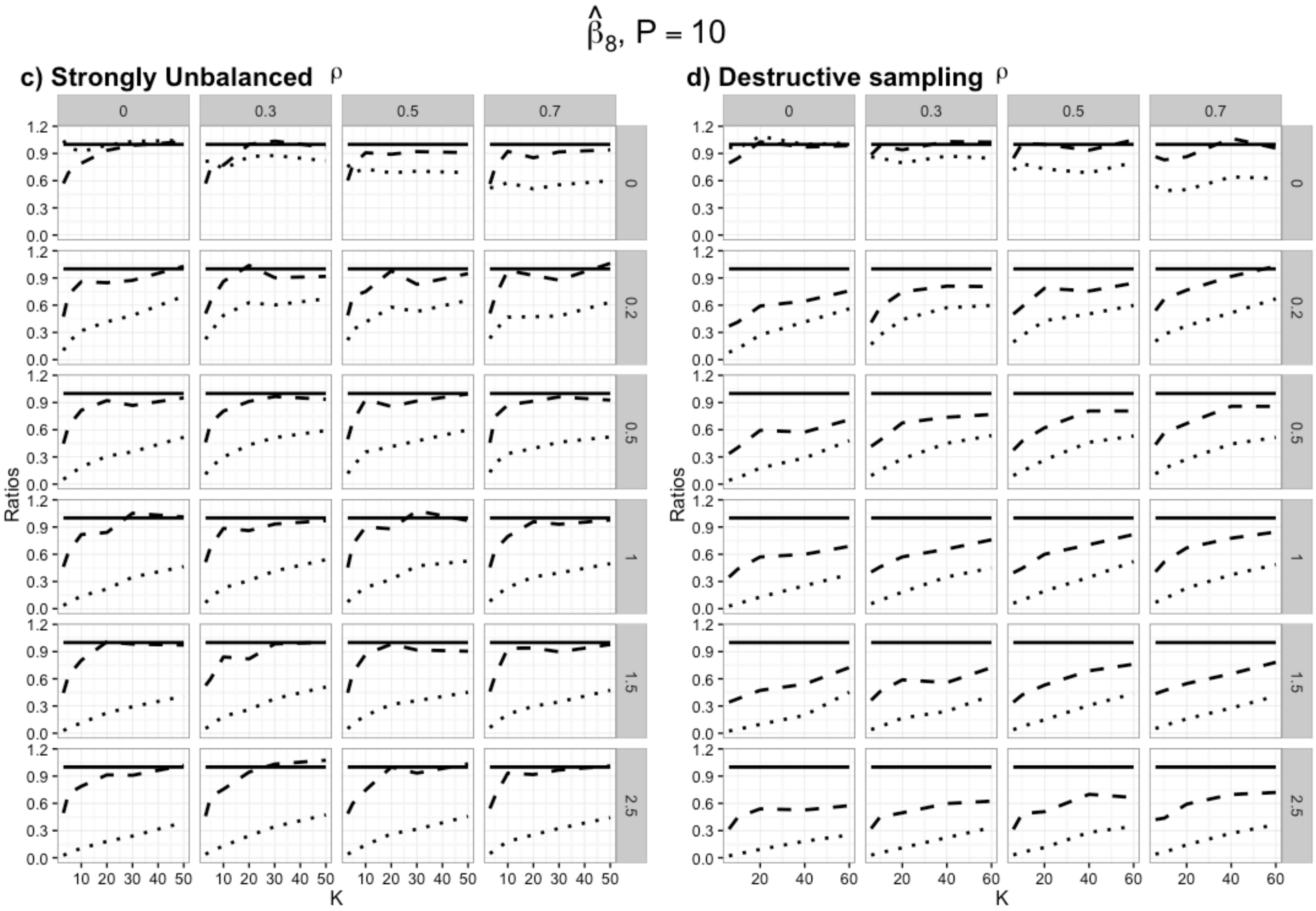

$\hat{\beta}_8$, P = 10
c) Strongly Unbalanced
d) Destructive sampling
ρ
0
0.3
0.5
0.7
0
0.2
0.5
1
1.5
2.5
Ratios
K
1.2
0.9
0.6
0.3
0.0
10 20 30 40 50
20 40 60

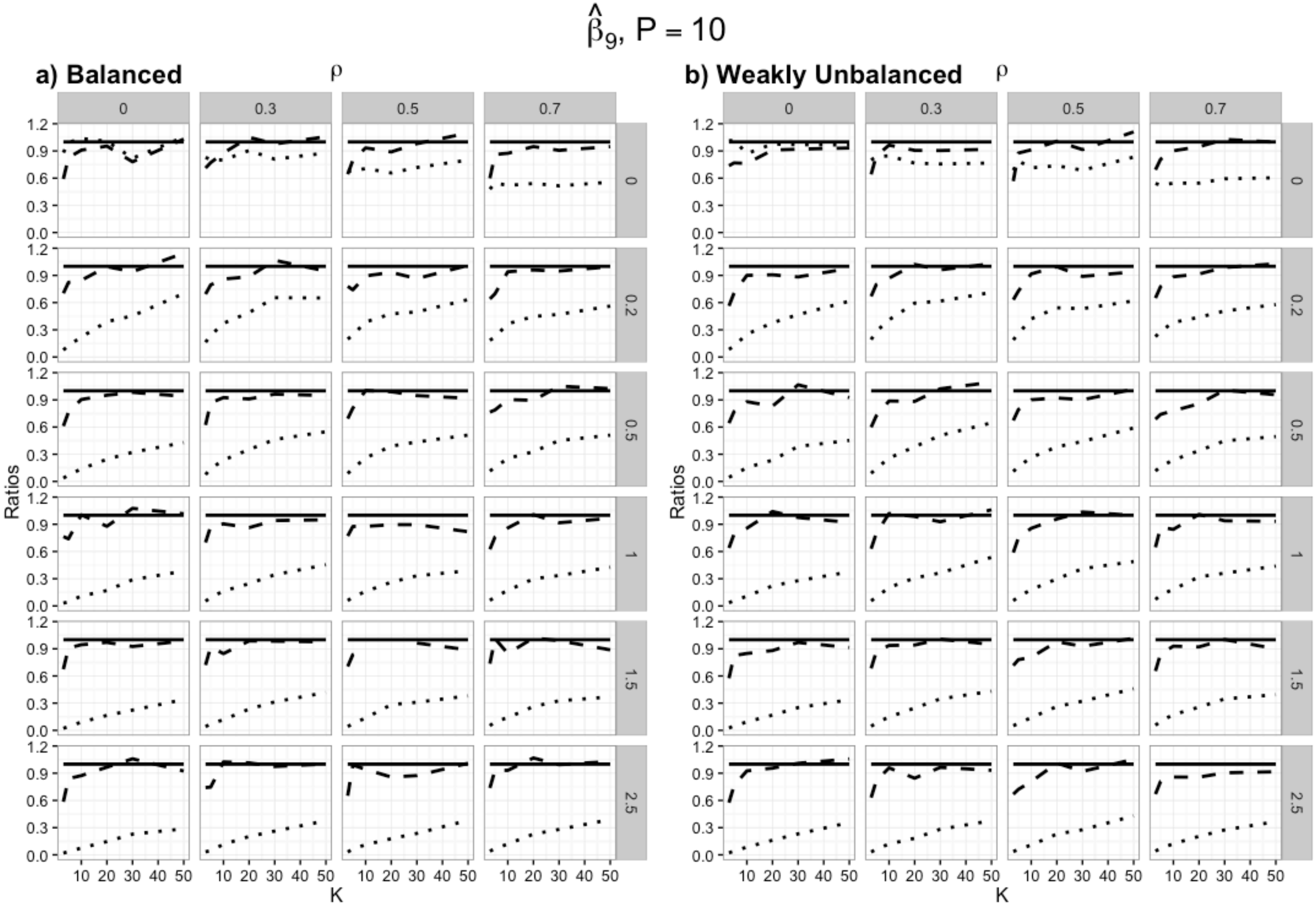

$\hat{\beta}_9$, P = 10
a) Balanced
b) Weakly Unbalanced
ρ
0
0.3
0.5
0.7
0.2
1
1.5
2.5
Ratios
K
0.0
0.3
0.6
0.9
1.2
10
20
30
40
50

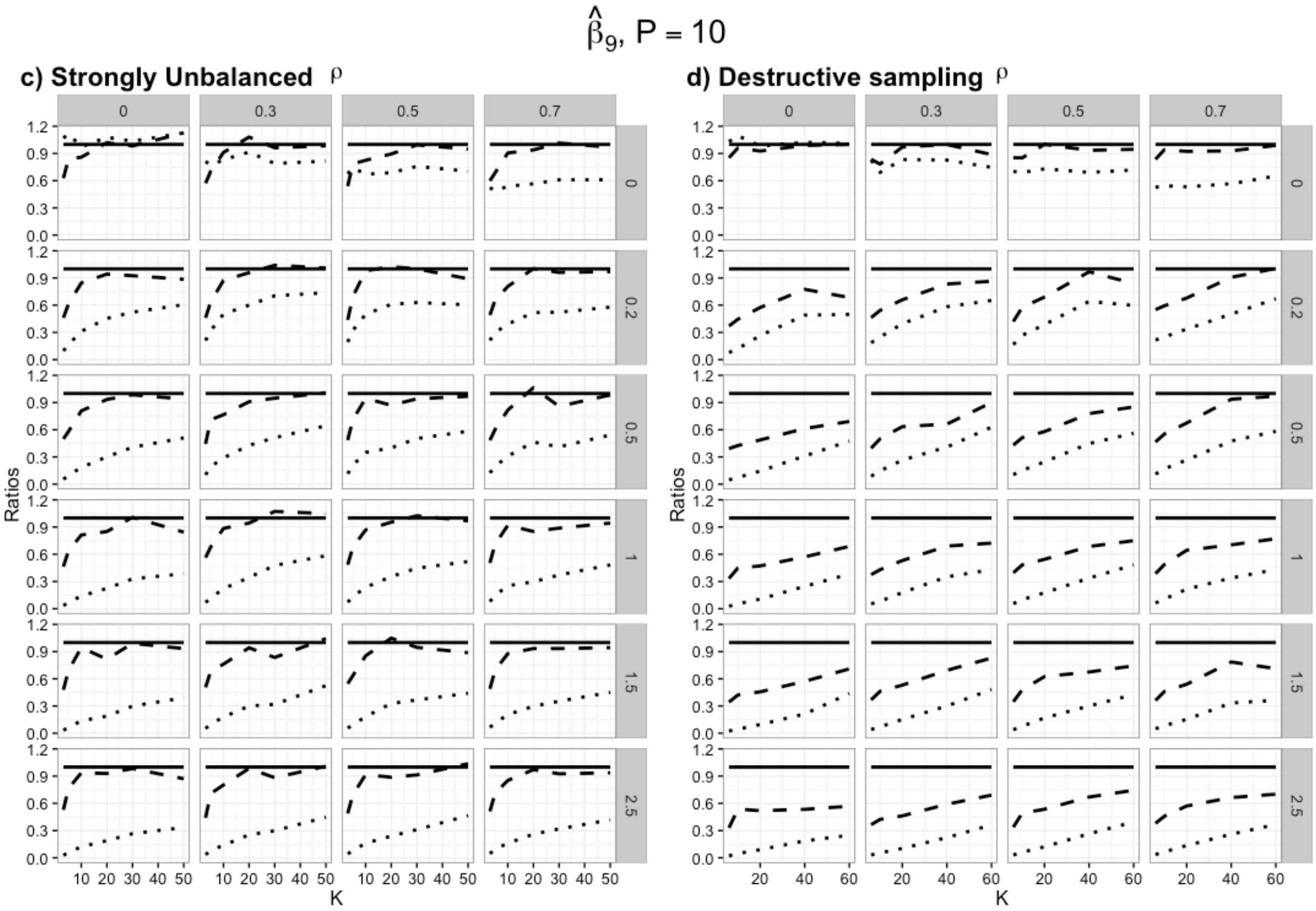
$\hat{\beta}_9$, P = 10
c) Strongly Unbalanced
d) Destructive sampling
ρ
0
0.3
0.5
0.7
0.2
1
1.5
2.5
Ratios
K
0.0
0.3
0.6
0.9
1.2
10 20 30 40 50
20 40 60

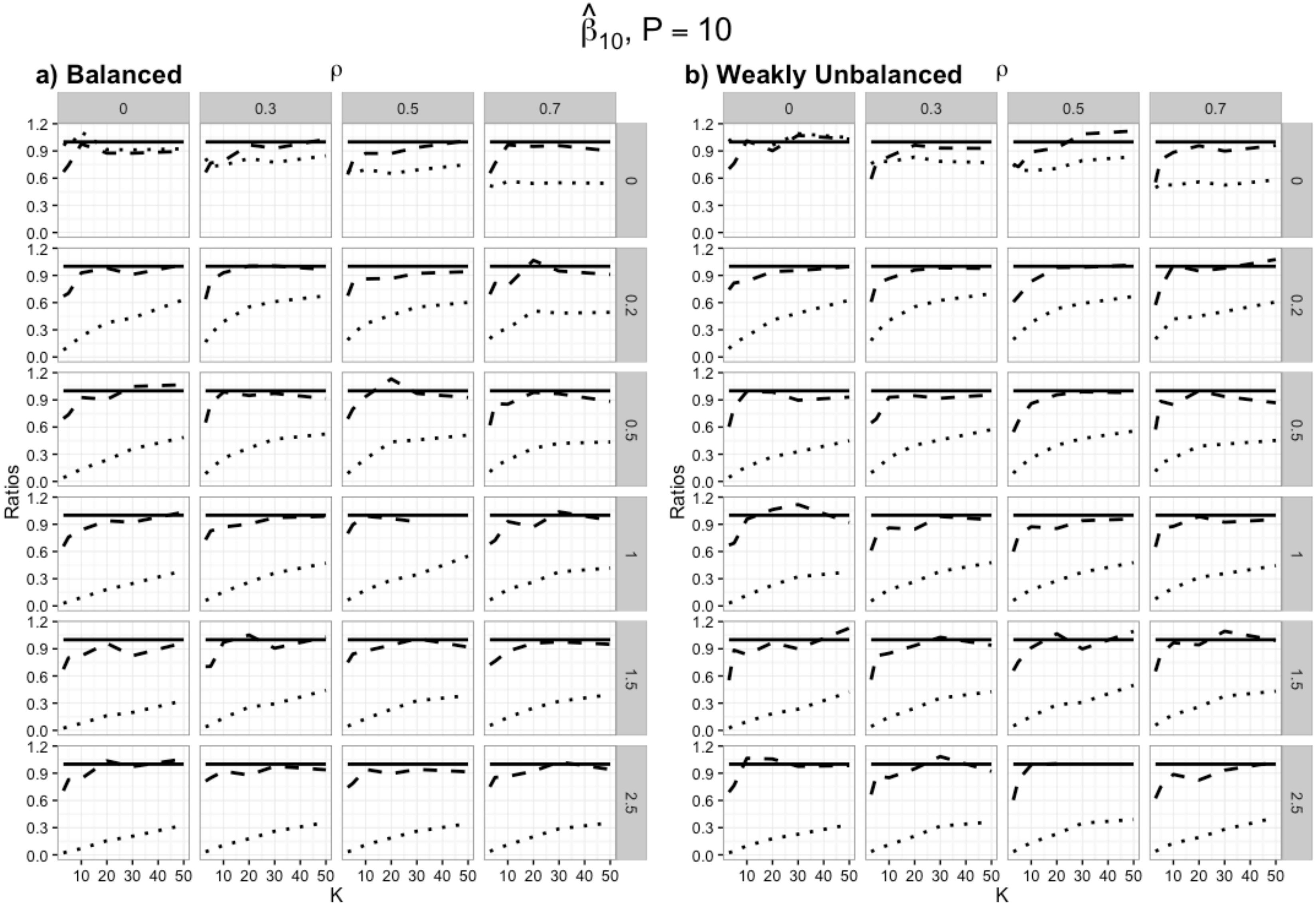

$\hat{\beta}_{10}$, P = 10
a) Balanced
b) Weakly Unbalanced
ρ
0
0.3
0.5
0.7
0
0.2
0.5
1
1.5
2.5
Ratios
K
1.2
0.9
0.6
0.3
0.0
10 20 30 40 50

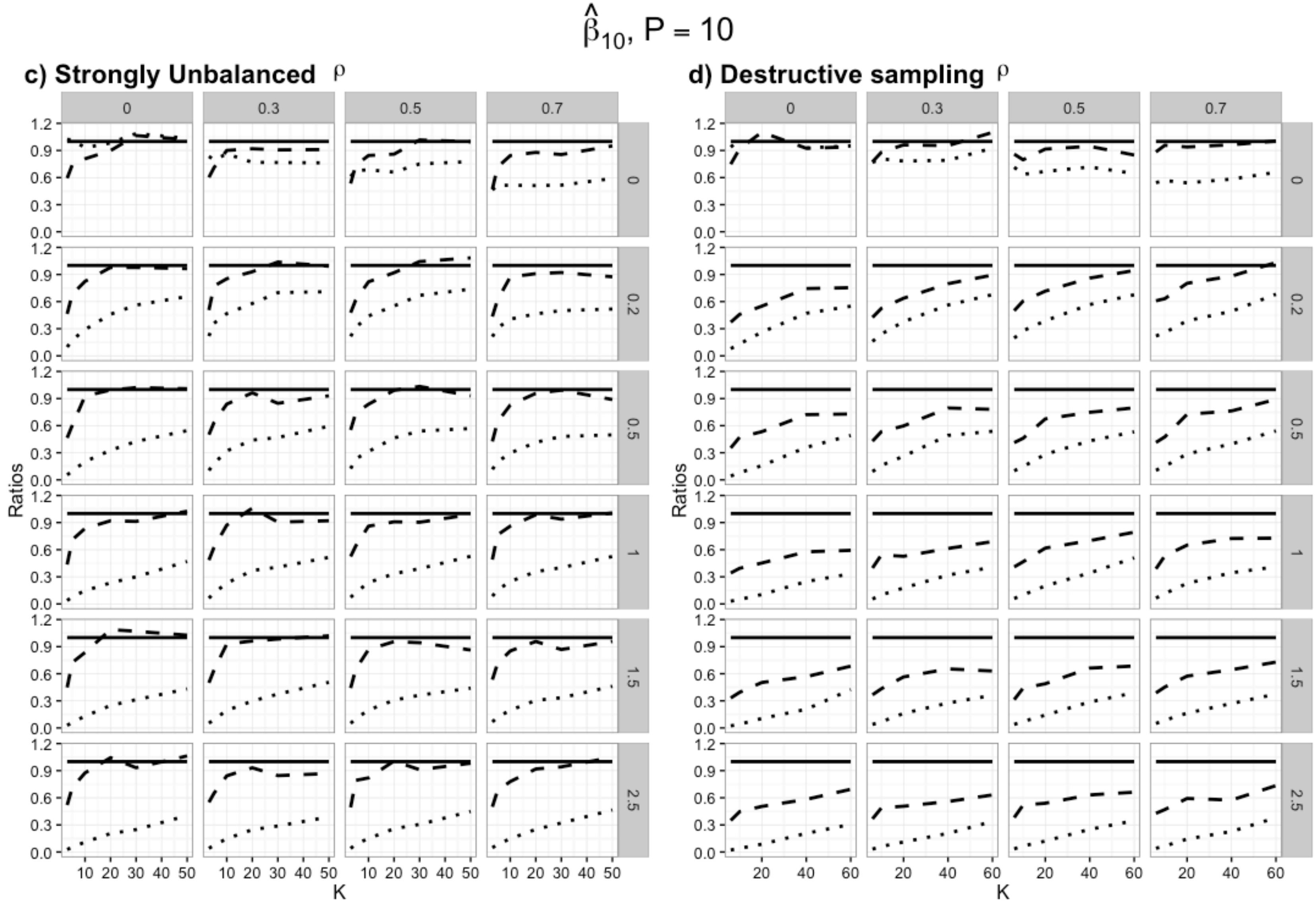
$\hat{\beta}_{10}$, P = 10
c) Strongly Unbalanced
d) Destructive sampling
ρ
0
0.3
0.5
0.7
0.2
1
1.5
2.5
Ratios
K
1.2
0.9
0.6
0.3
0.0
10
20
30
40
50
60
