# Supplementary material for: Robust Inference from Conditional Logistic Regression Applied to Movement and Habitat Selection Analysis
Source: PLoS One. 2017 Jan 12;12(1):e0169779. doi: 10.1371/journal.pone.0169779 (PMC5233429; doi:10.1371/journal.pone.0169779)
Supplement: S3 Fig — Average estimates of β^p for different number of covariates (P) and different number of clusters (K), as a function of temporal autocorrelation (ρ) and inter-individual heterogeneity (σH2 on the left side of the panels) as well as different data processing: a) Balanced, b) Weakly Unbalanced, c) Strongly Unbalanced and d) Destructive sampling. Fixed values of βp, p ∈ {1,…,P} were equal to: β1fixed=0.75 and β2fixed=0.5 when P = 2 and β1fixed=0.75; β2fixed=0.75; β3fixed=0.75; β4fixed=0.5; β5fixed=0.5; β6fixed=0.5; β7fixed=0.2; β8fixed=0.2; β9fixed=0.2 and β10fixed=0.2 when P = 10. (PDF) [file pone.0169779.s004.pdf]

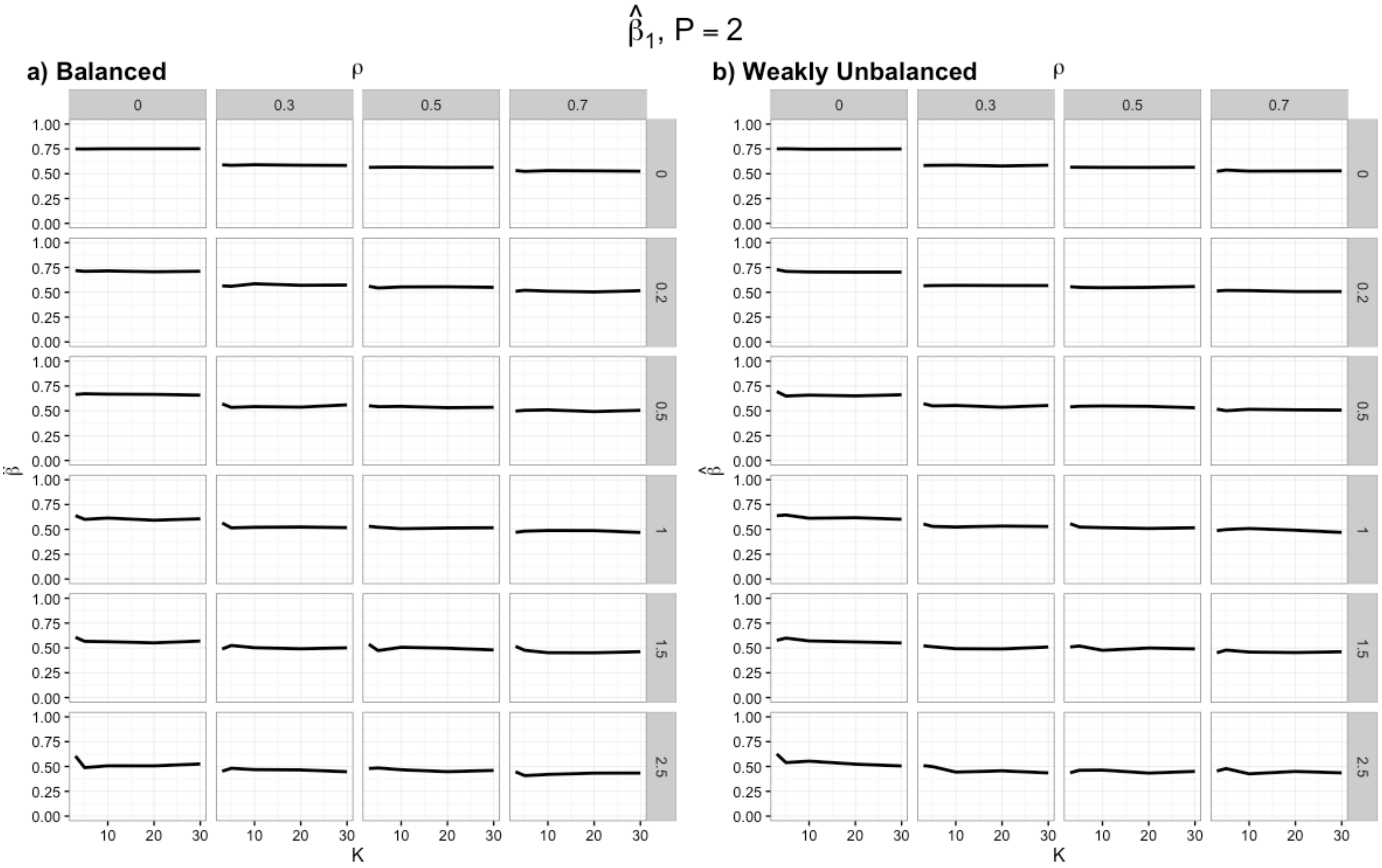

$\hat{\beta}_1$, P = 2
a) Balanced
b) Weakly Unbalanced
ρ
0
0.3
0.5
0.7
0
0.2
0.5
1
1.5
2.5
$\hat{\beta}$
K
1.00
0.75
0.50
0.25
0.00
10
20
30

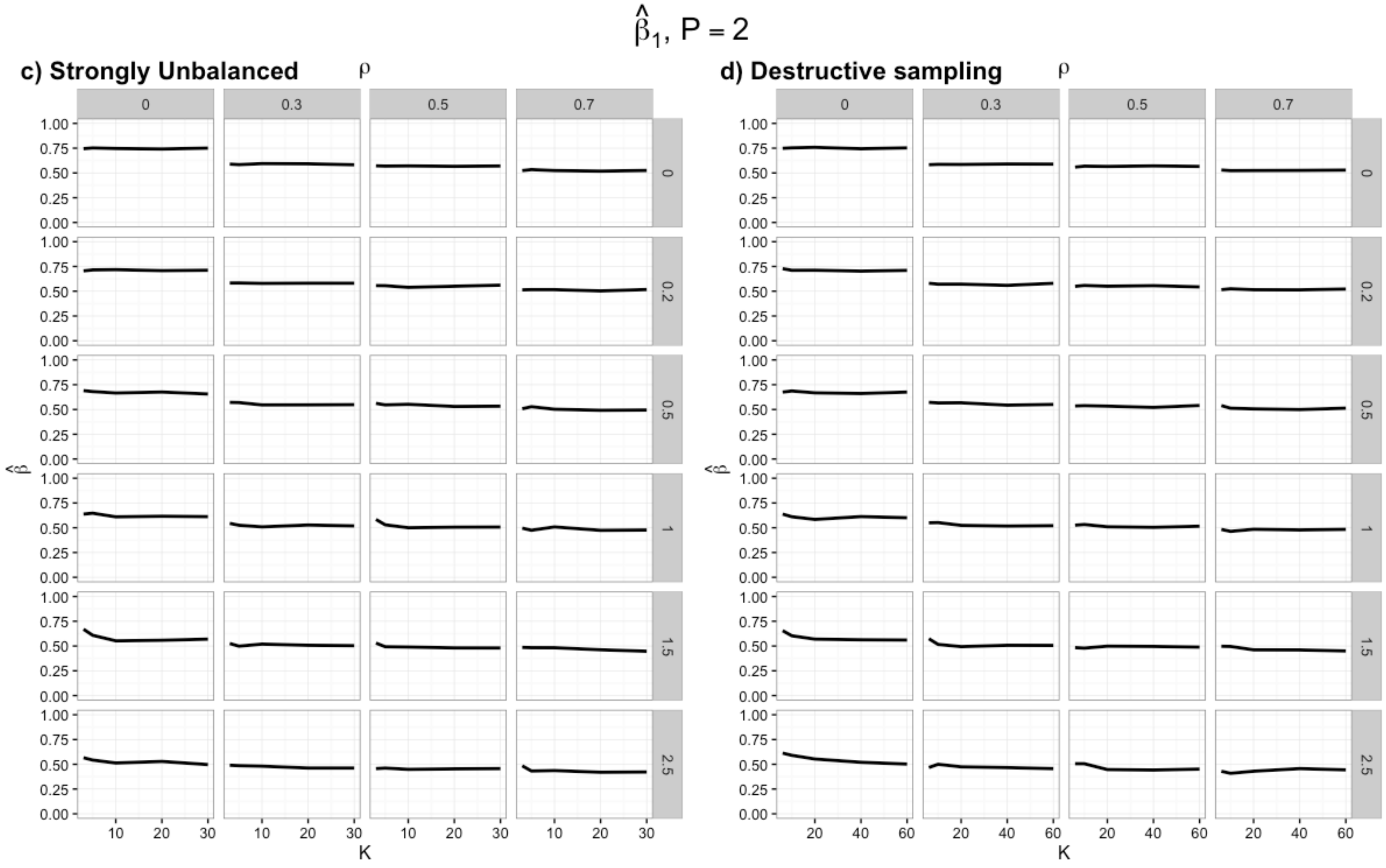

β̂1, P = 2
c) Strongly Unbalanced
d) Destructive sampling
ρ
0
0.3
0.5
0.7
0
0.2
0.5
1
1.5
2.5
β̂
K

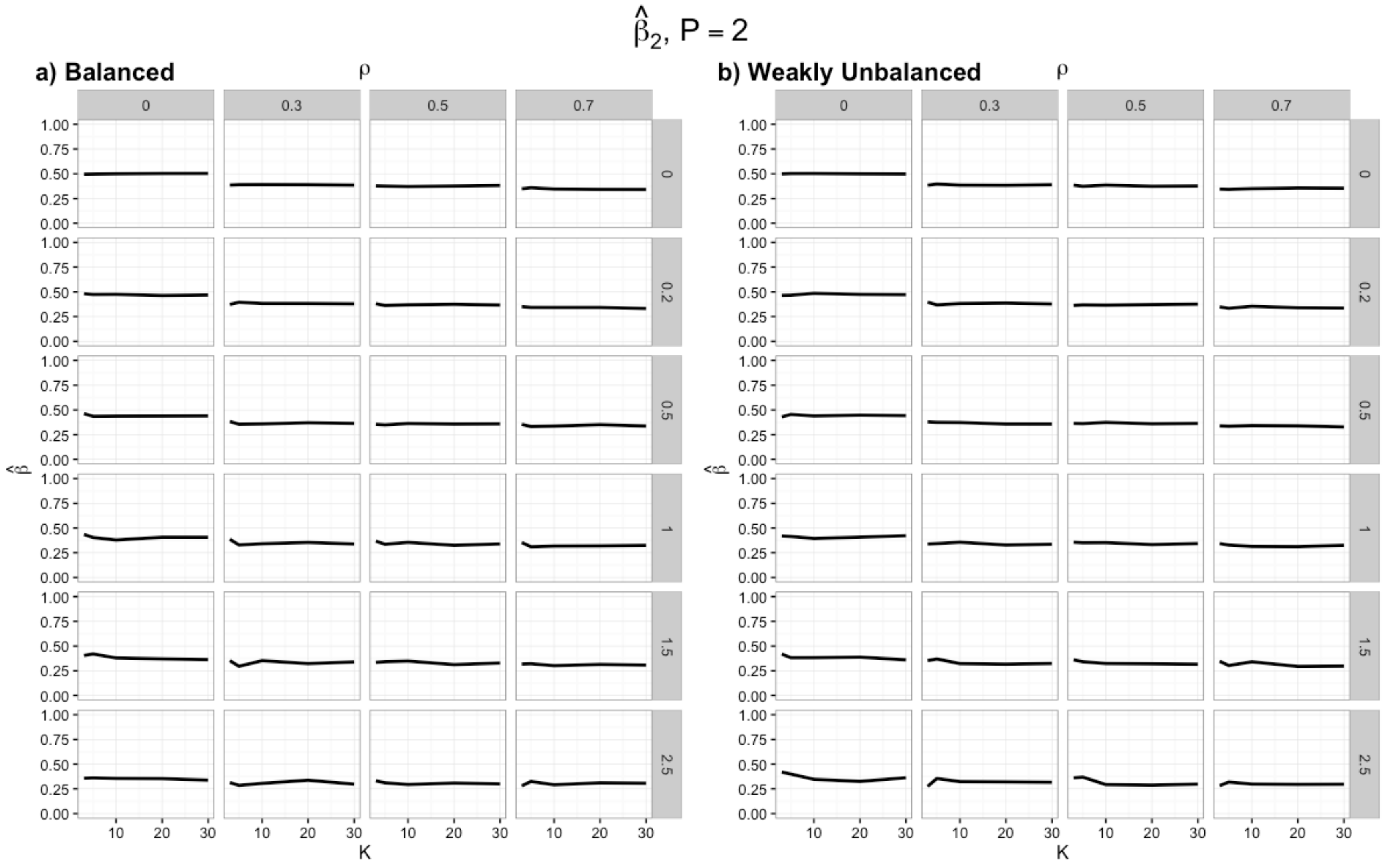
$\hat{\beta}_2$, P = 2
a) Balanced
b) Weakly Unbalanced
ρ
0
0.3
0.5
0.7
0
0.2
0.5
1
1.5
2.5
$\hat{\beta}$
K
1.00
0.75
0.50
0.25
0.00
10
20
30

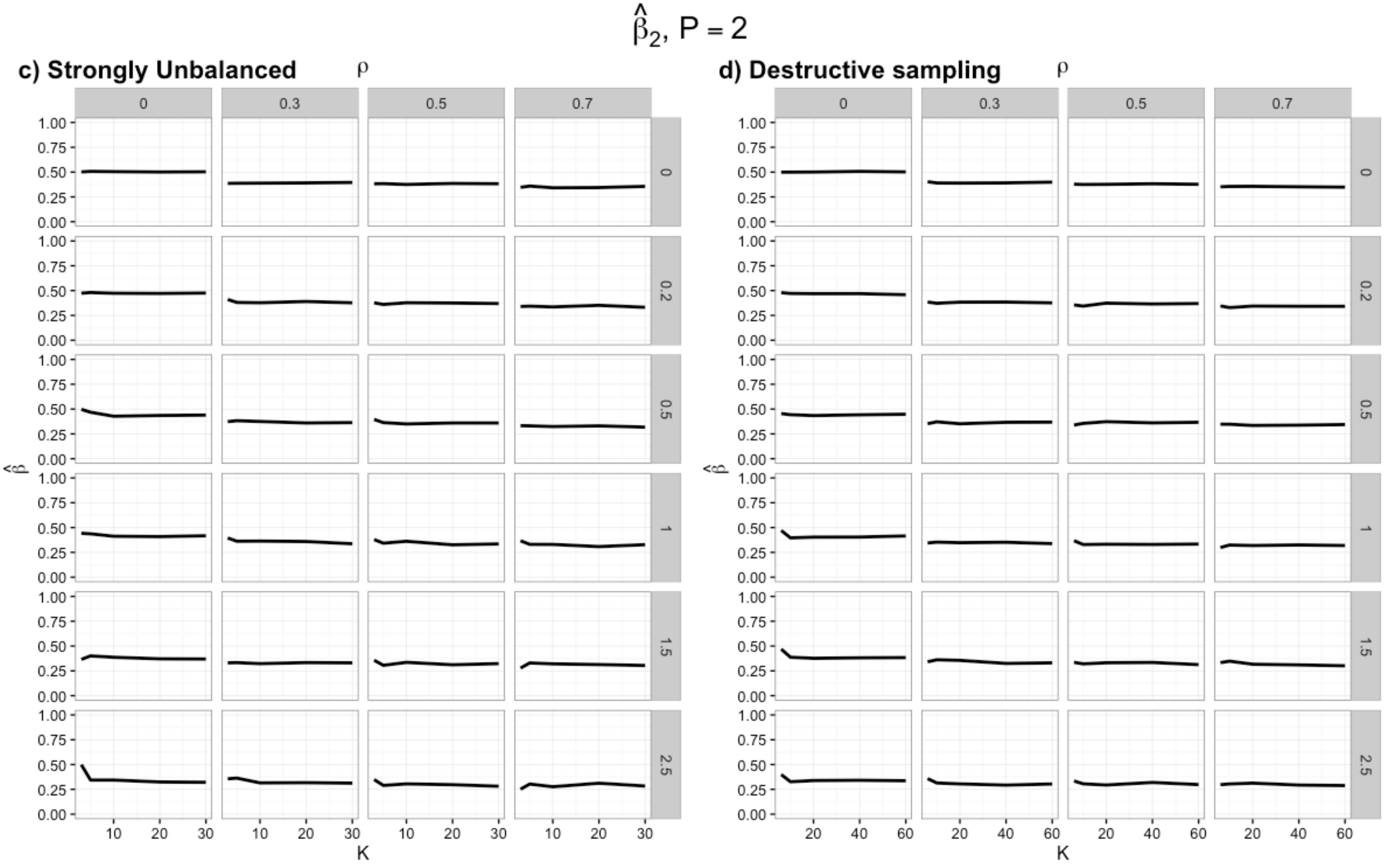
$\hat{\beta}_2$, P = 2
c) Strongly Unbalanced
d) Destructive sampling
ρ
0
0.3
0.5
0.7
0
0.2
0.5
1
1.5
2.5
1.00
0.75
0.50
0.25
0.00
$\hat{\beta}$
10
20
30
40
60
K

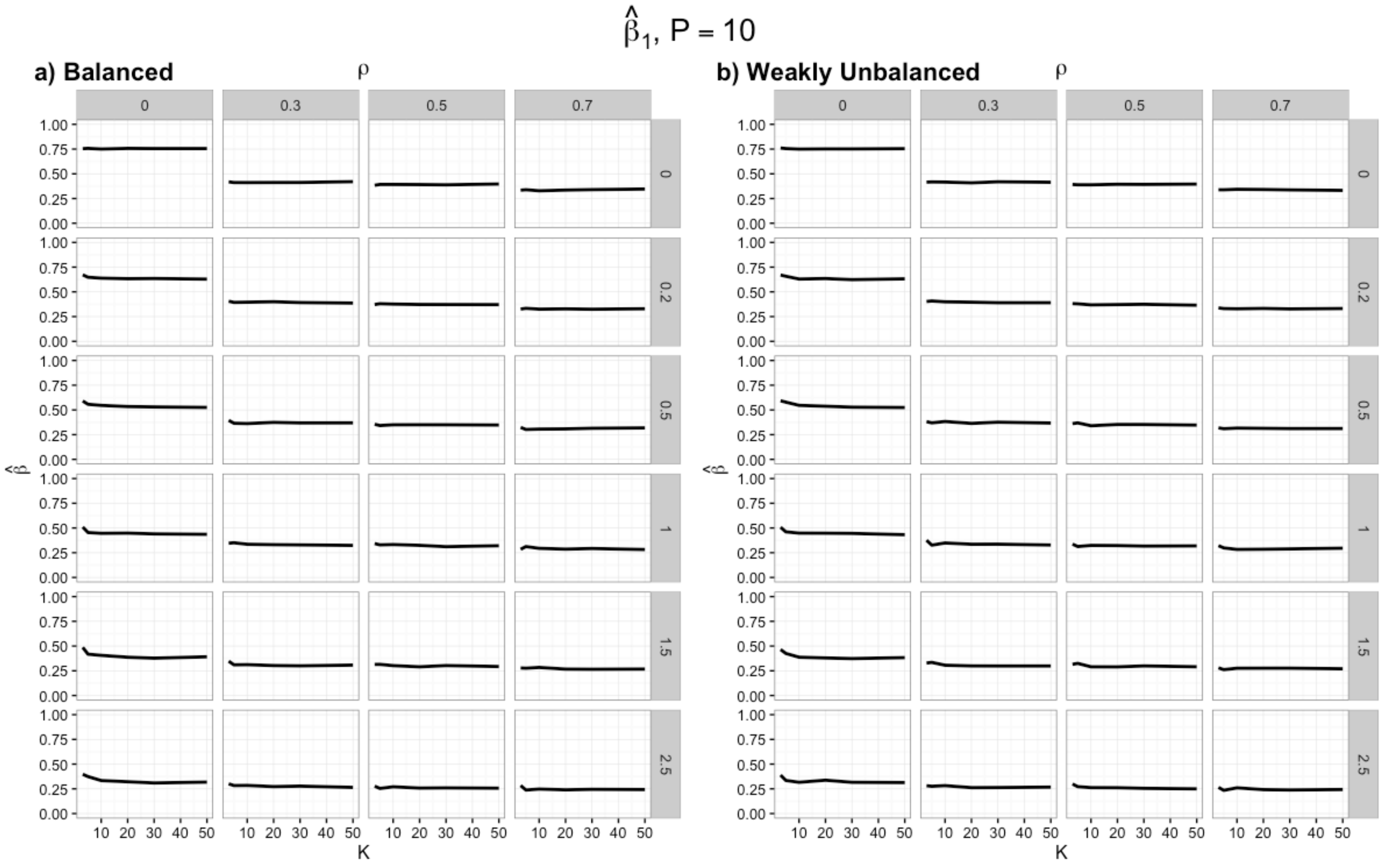
$\hat{\beta}_1$, P = 10
a) Balanced
b) Weakly Unbalanced
ρ
0
0.3
0.5
0.7
0
0.2
0.5
1
1.5
2.5
1.00
0.75
0.50
0.25
0.00
$\hat{\beta}$
10
20
30
40
50
K

$\hat{\beta}_1$, P = 10

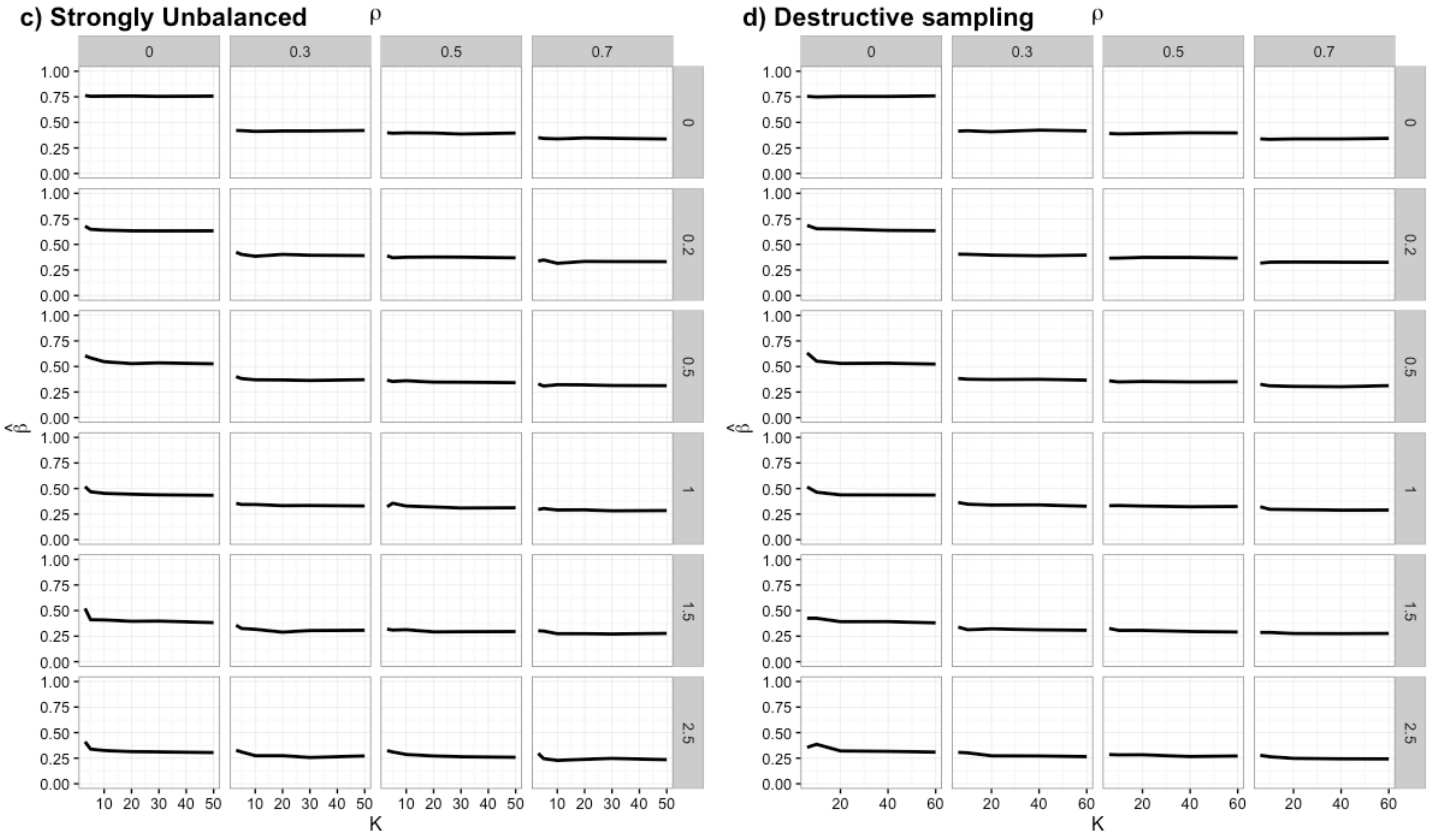

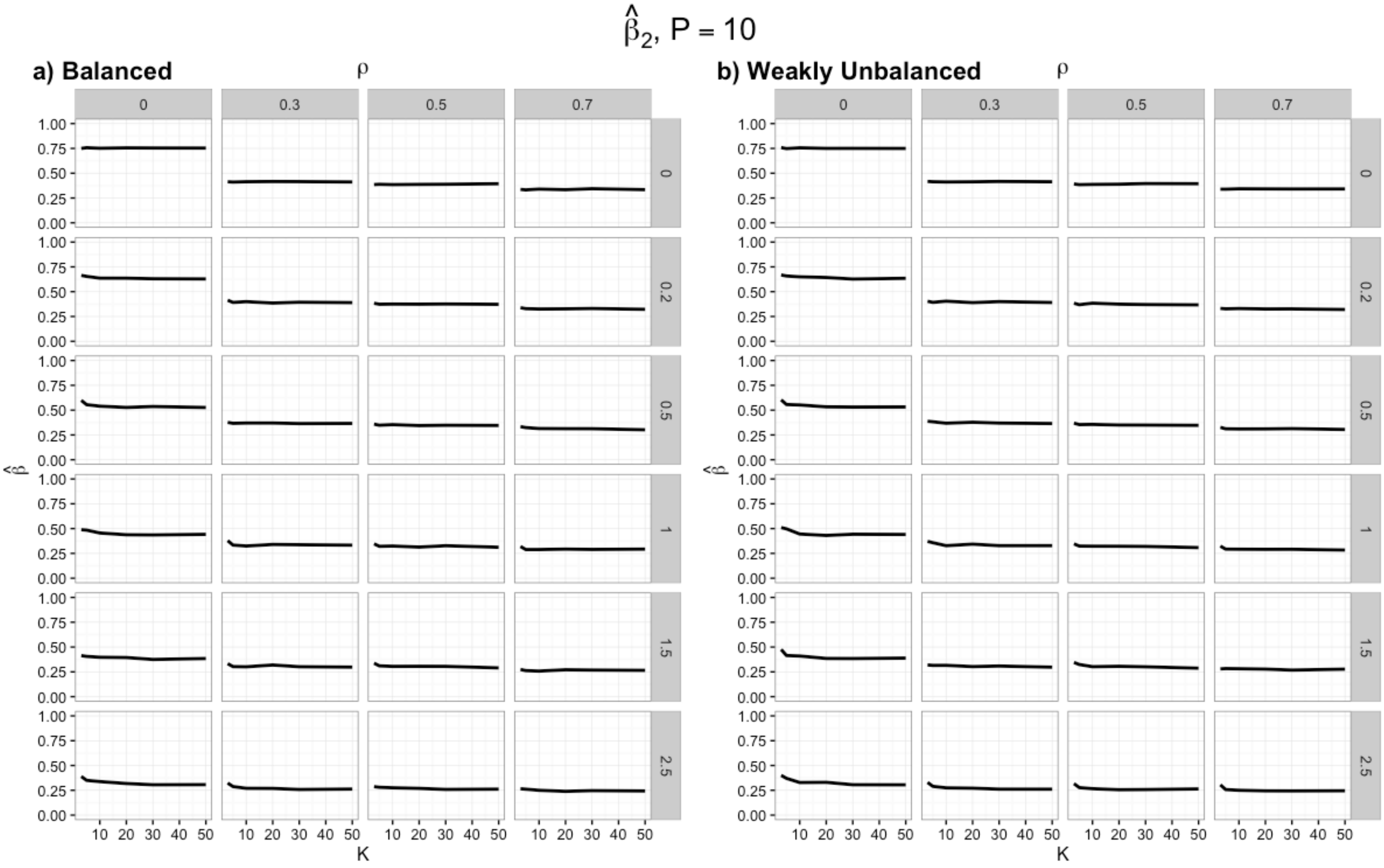
$\hat{\beta}_2$, P = 10
a) Balanced
b) Weakly Unbalanced
ρ
0
0.3
0.5
0.7
0
0.2
0.5
1
1.5
2.5
1.00
0.75
0.50
0.25
0.00
$\hat{\beta}$
10 20 30 40 50
K

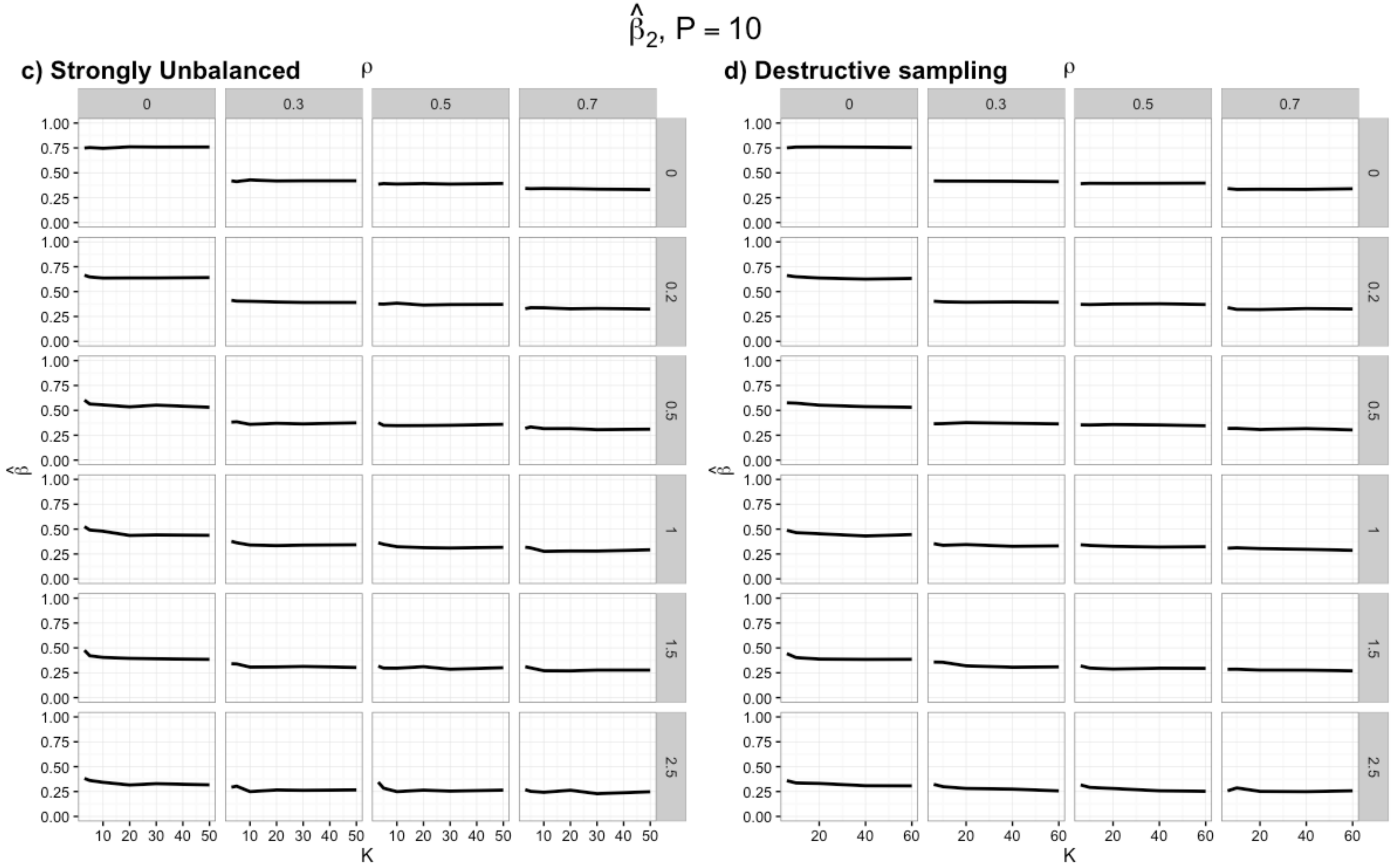

$\hat{\beta}_2$, P = 10
c) Strongly Unbalanced
d) Destructive sampling
ρ
0
0.3
0.5
0.7
0
0.2
0.5
1
1.5
2.5
$\hat{\beta}$
1.00
0.75
0.50
0.25
0.00
10 20 30 40 50
20 40 60
K

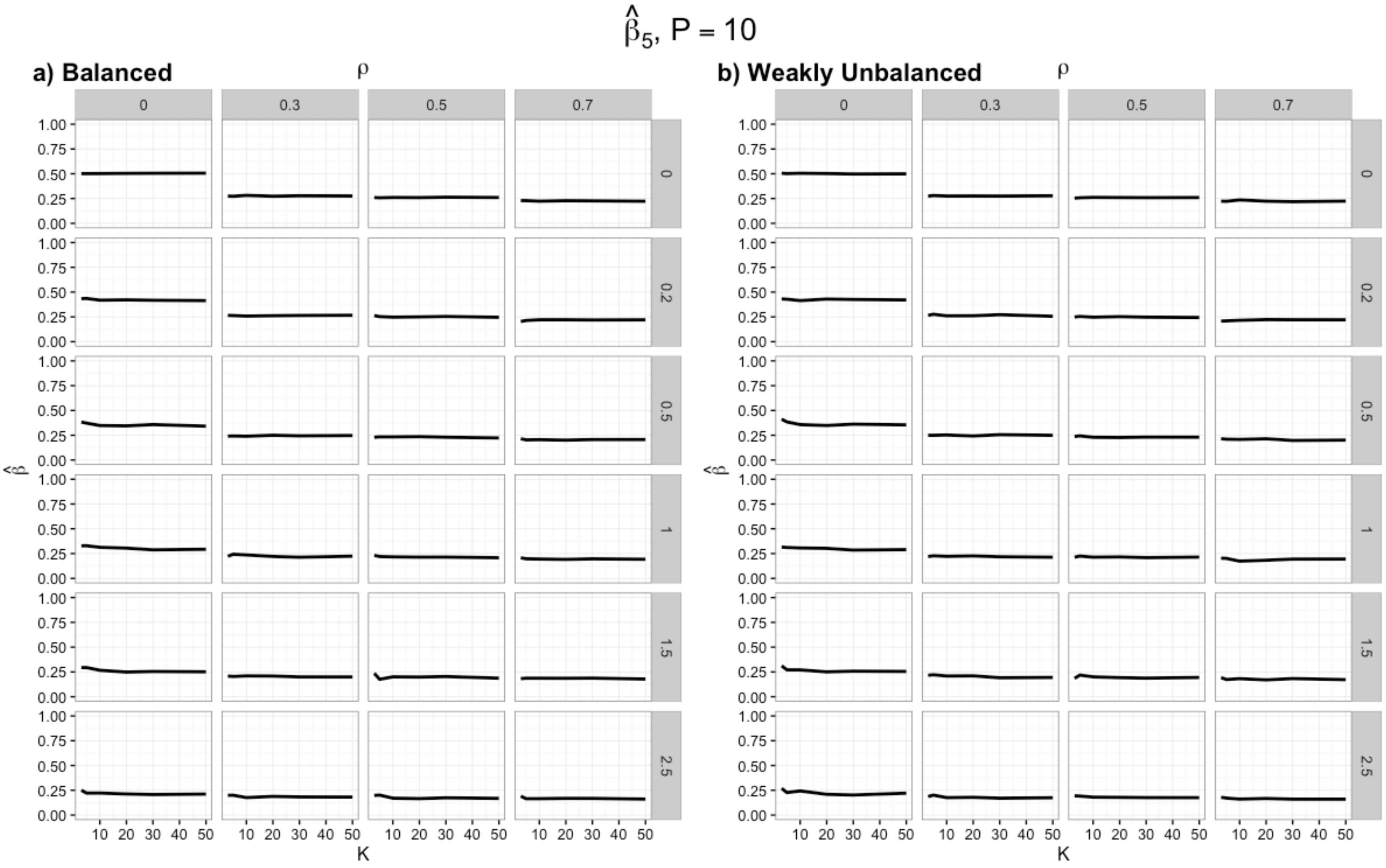
$\hat{\beta}_5$, P = 10
a) Balanced
b) Weakly Unbalanced
ρ
0
0.3
0.5
0.7
0
0.2
0.5
1
1.5
2.5
1.00
0.75
0.50
0.25
0.00
$\hat{\beta}$
10 20 30 40 50
K

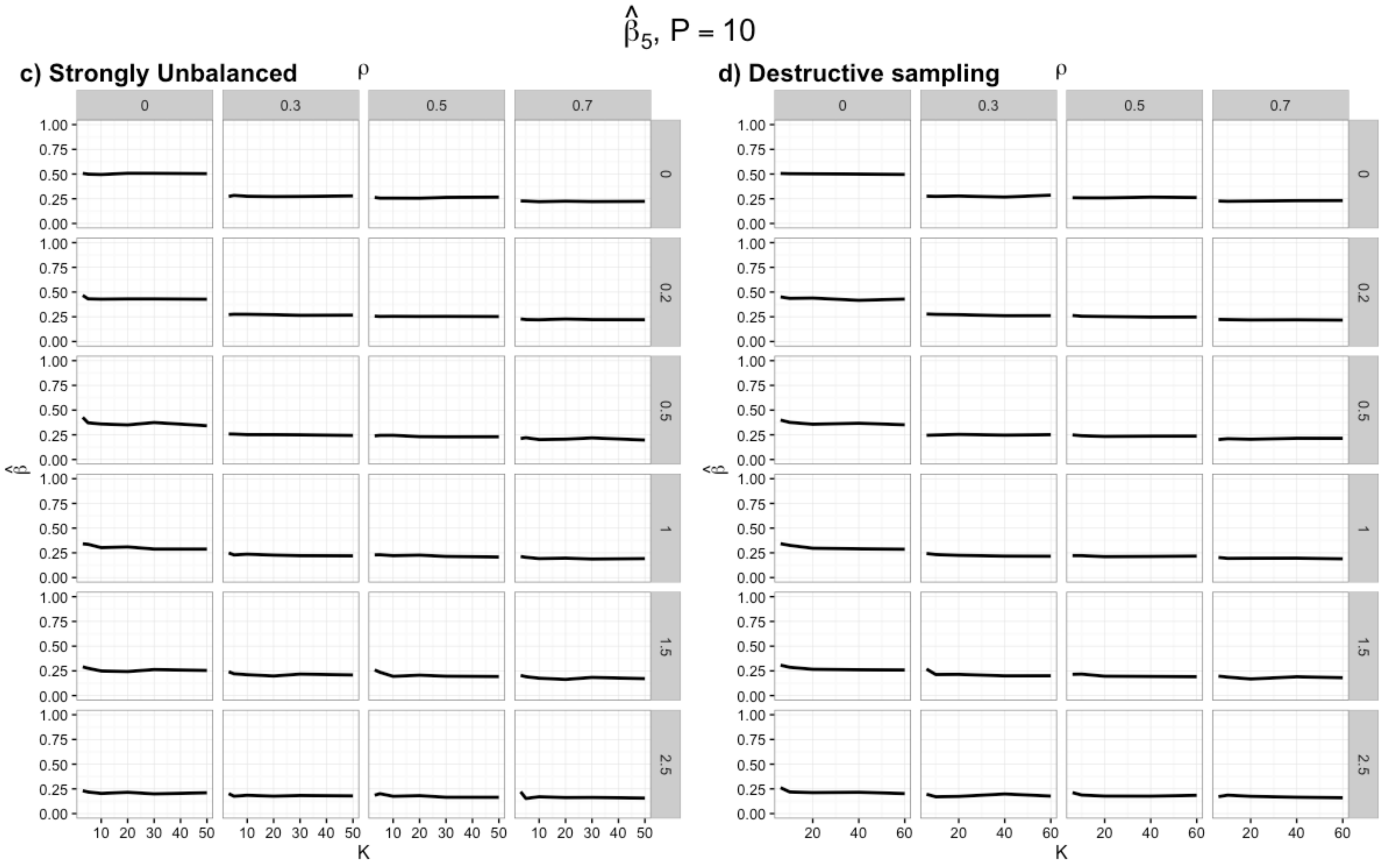

$\hat{\beta}_5$, P = 10
c) Strongly Unbalanced
d) Destructive sampling
ρ
0
0.3
0.5
0.7
0
0.2
0.5
1
1.5
2.5
1.00
0.75
0.50
0.25
0.00
$\hat{\beta}$
K
10
20
30
40
50
60

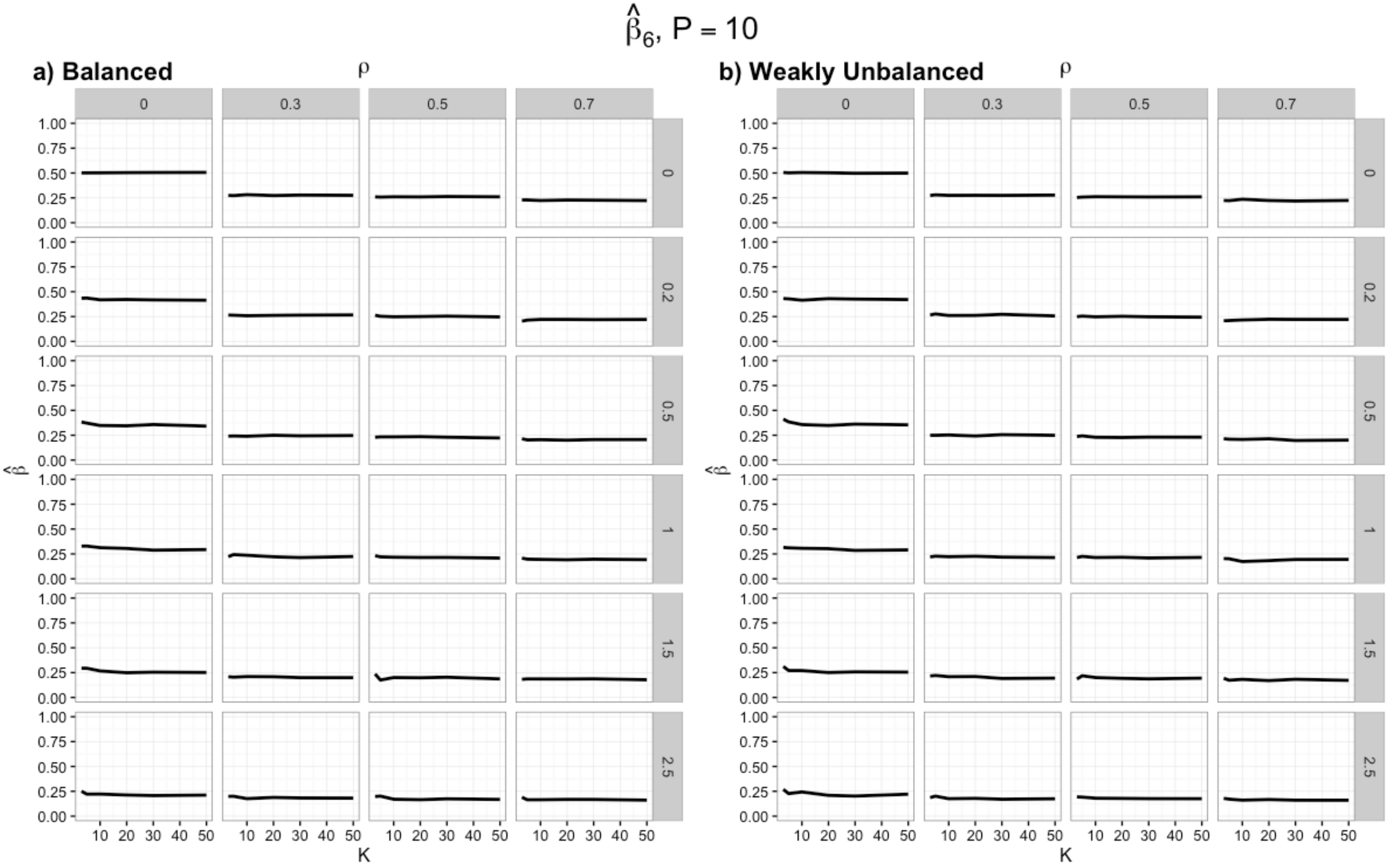

$\hat{\beta}_6$, P = 10
a) Balanced
b) Weakly Unbalanced
ρ
0
0.3
0.5
0.7
0
0.2
0.5
1
1.5
2.5
1.00
0.75
0.50
0.25
0.00
$\hat{\beta}$
10
20
30
40
50
K

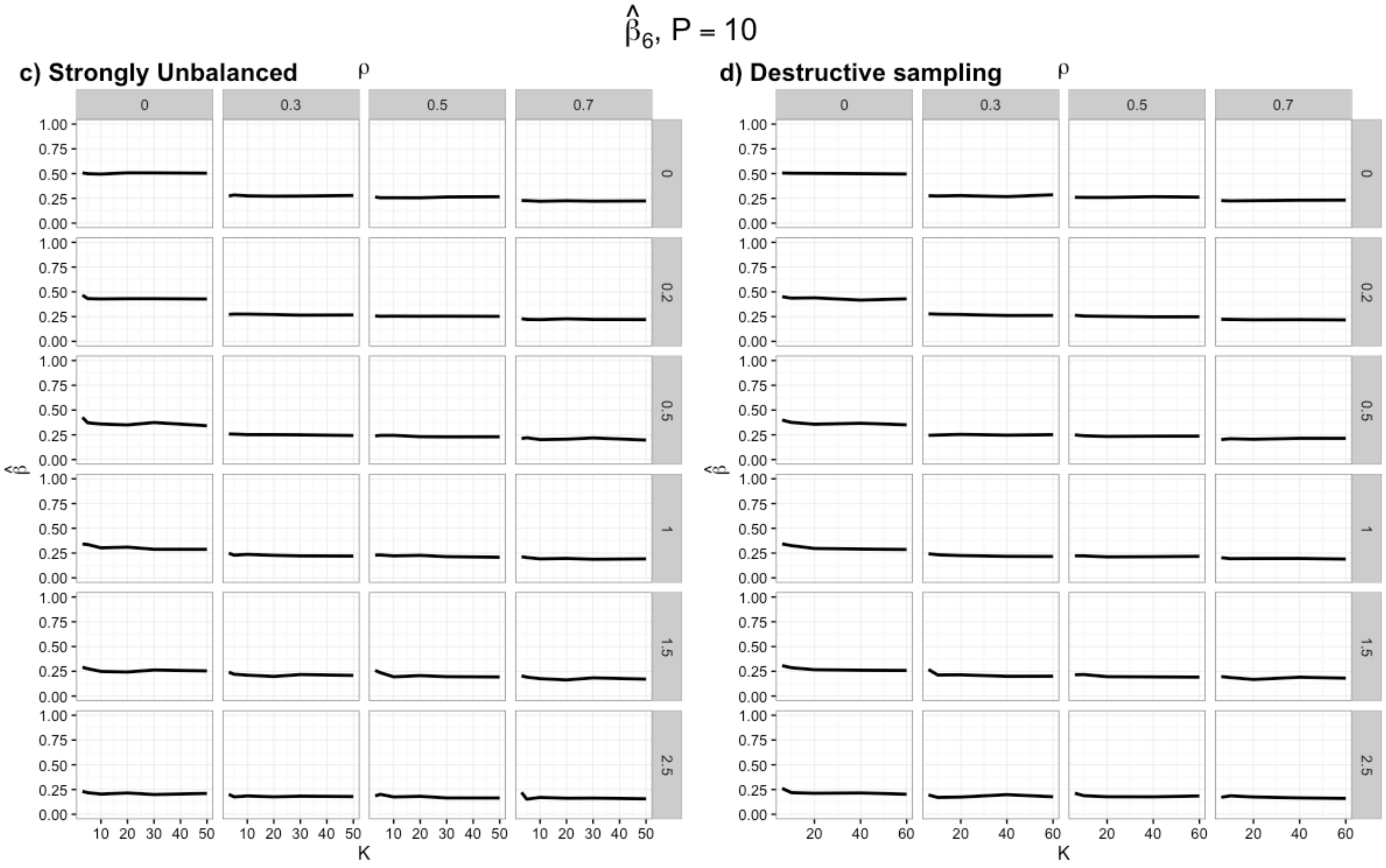

$\hat{\beta}_6$, P = 10
c) Strongly Unbalanced
d) Destructive sampling
ρ
0
0.3
0.5
0.7
0
0.2
0.5
1
1.5
2.5
1.00
0.75
0.50
0.25
0.00
$\hat{\beta}$
10 20 30 40 50
20 40 60
K

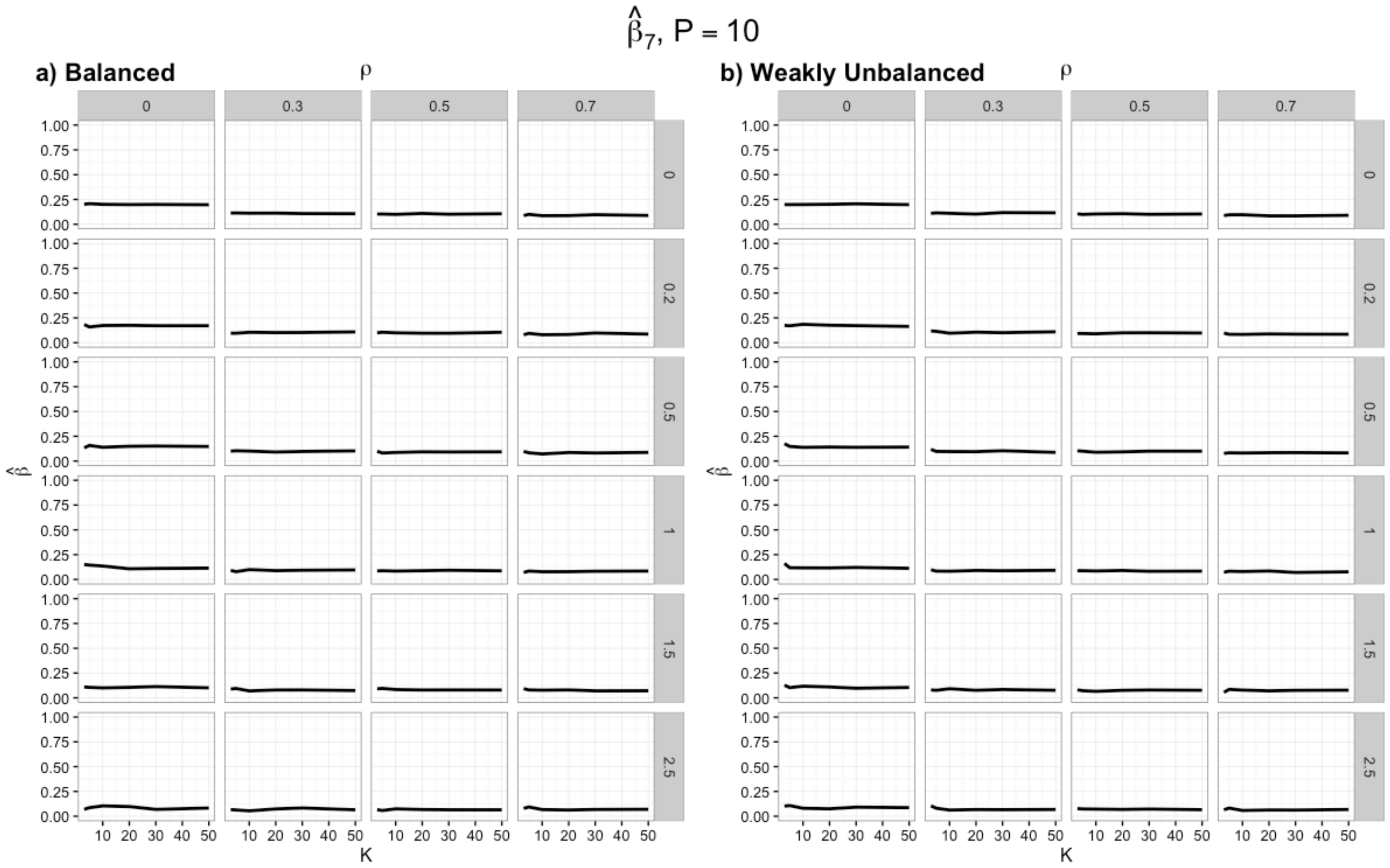

$\hat{\beta}_7$, P = 10
a) Balanced
b) Weakly Unbalanced
ρ
0
0.3
0.5
0.7
0
0.2
0.5
1
1.5
2.5
1.00
0.75
0.50
0.25
0.00
$\hat{\beta}$
10 20 30 40 50
K

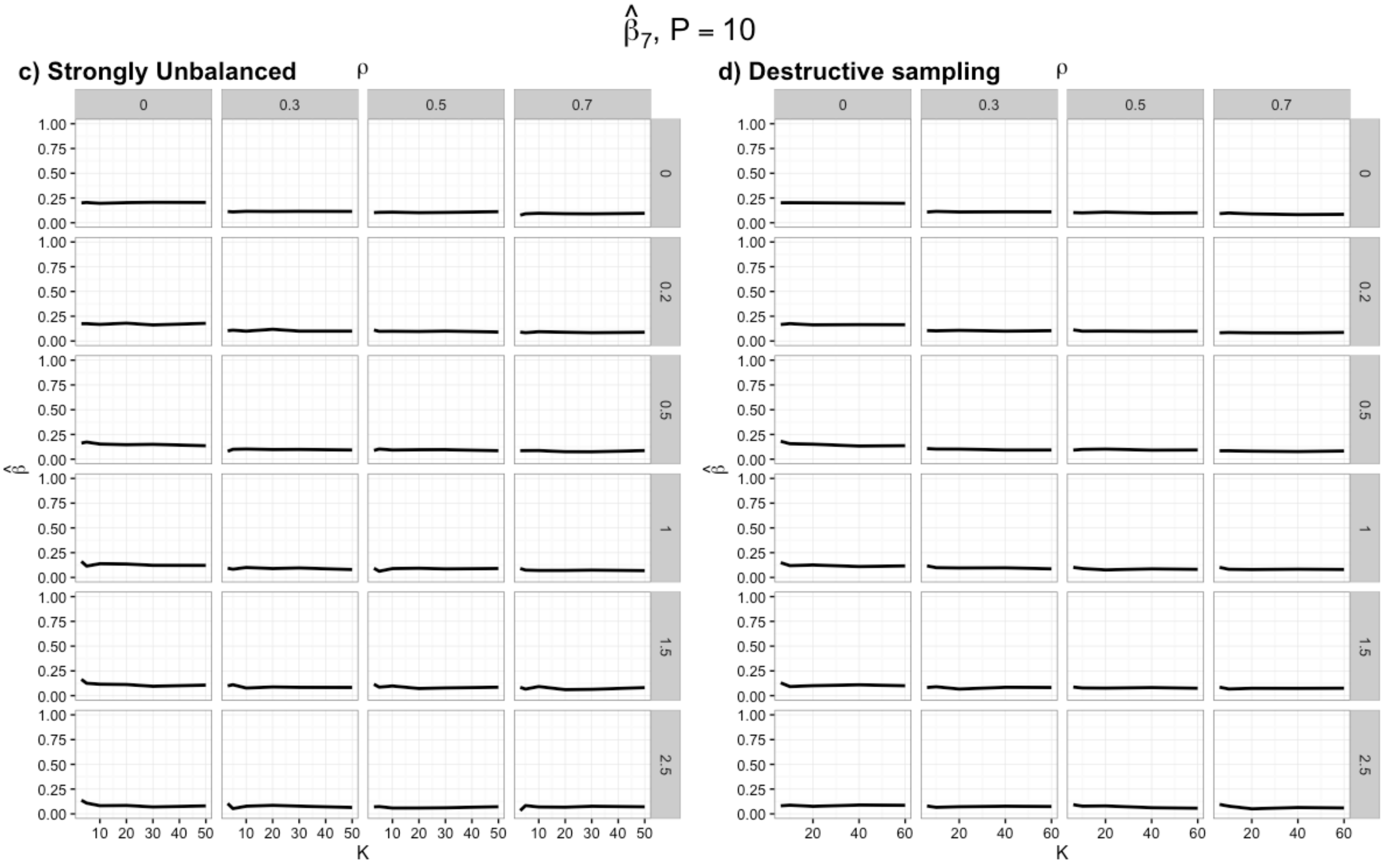

$\hat{\beta}_7$, P = 10
c) Strongly Unbalanced
d) Destructive sampling
ρ
0
0.3
0.5
0.7
0
0.2
0.5
1
1.5
2.5
1.00
0.75
0.50
0.25
0.00
$\hat{\beta}$
10 20 30 40 50
20 40 60
K

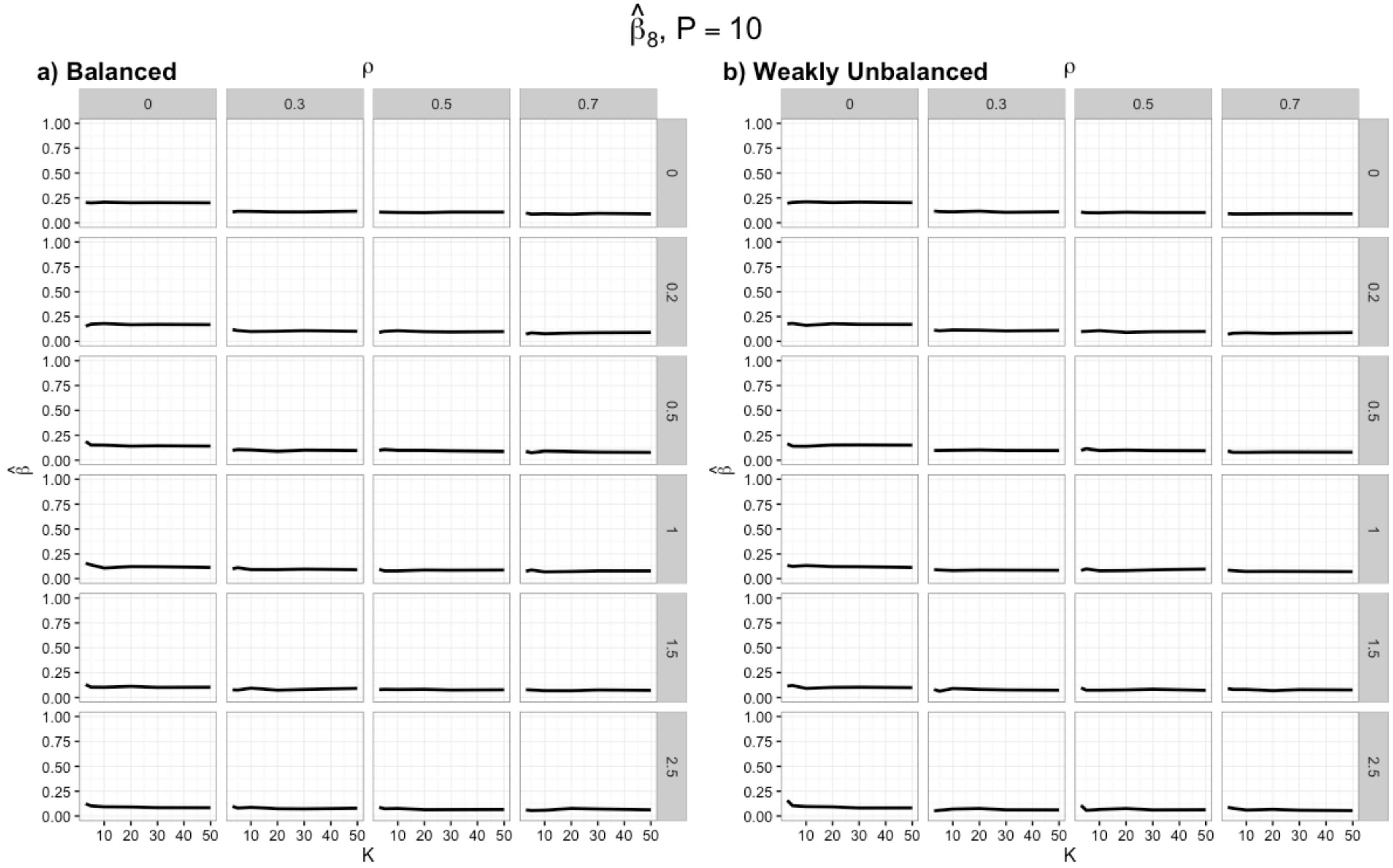
$\hat{\beta}_8$, P = 10
a) Balanced
b) Weakly Unbalanced
ρ
0
0.3
0.5
0.7
0
0.2
0.5
1
1.5
2.5
1.00
0.75
0.50
0.25
0.00
$\hat{\beta}$
10 20 30 40 50
K

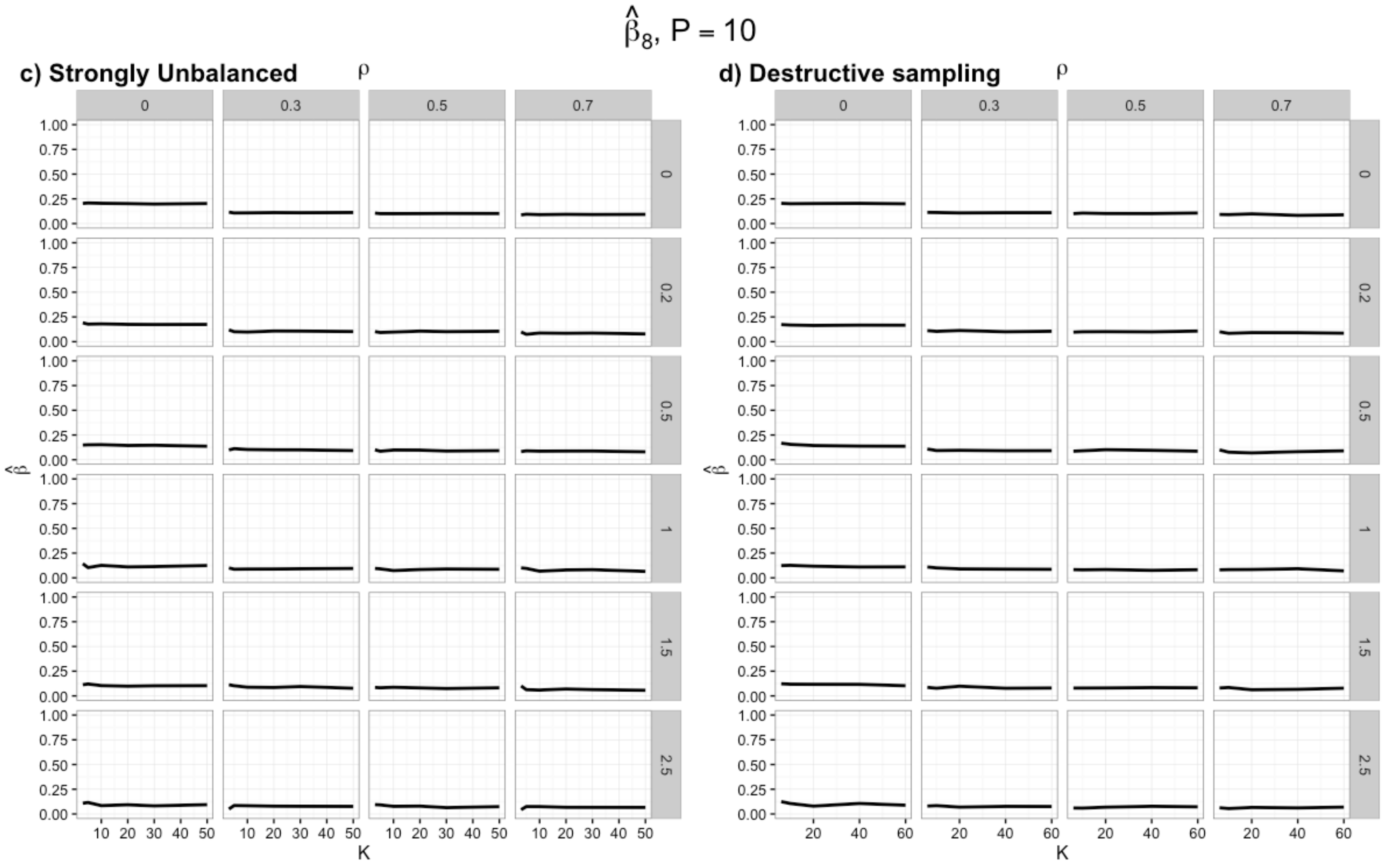

$\hat{\beta}_8$, P = 10
c) Strongly Unbalanced
d) Destructive sampling
ρ
0
0.3
0.5
0.7
0
0.2
0.5
1
1.5
2.5
1.00
0.75
0.50
0.25
0.00
$\hat{\beta}$
10 20 30 40 50
20 40 60
K

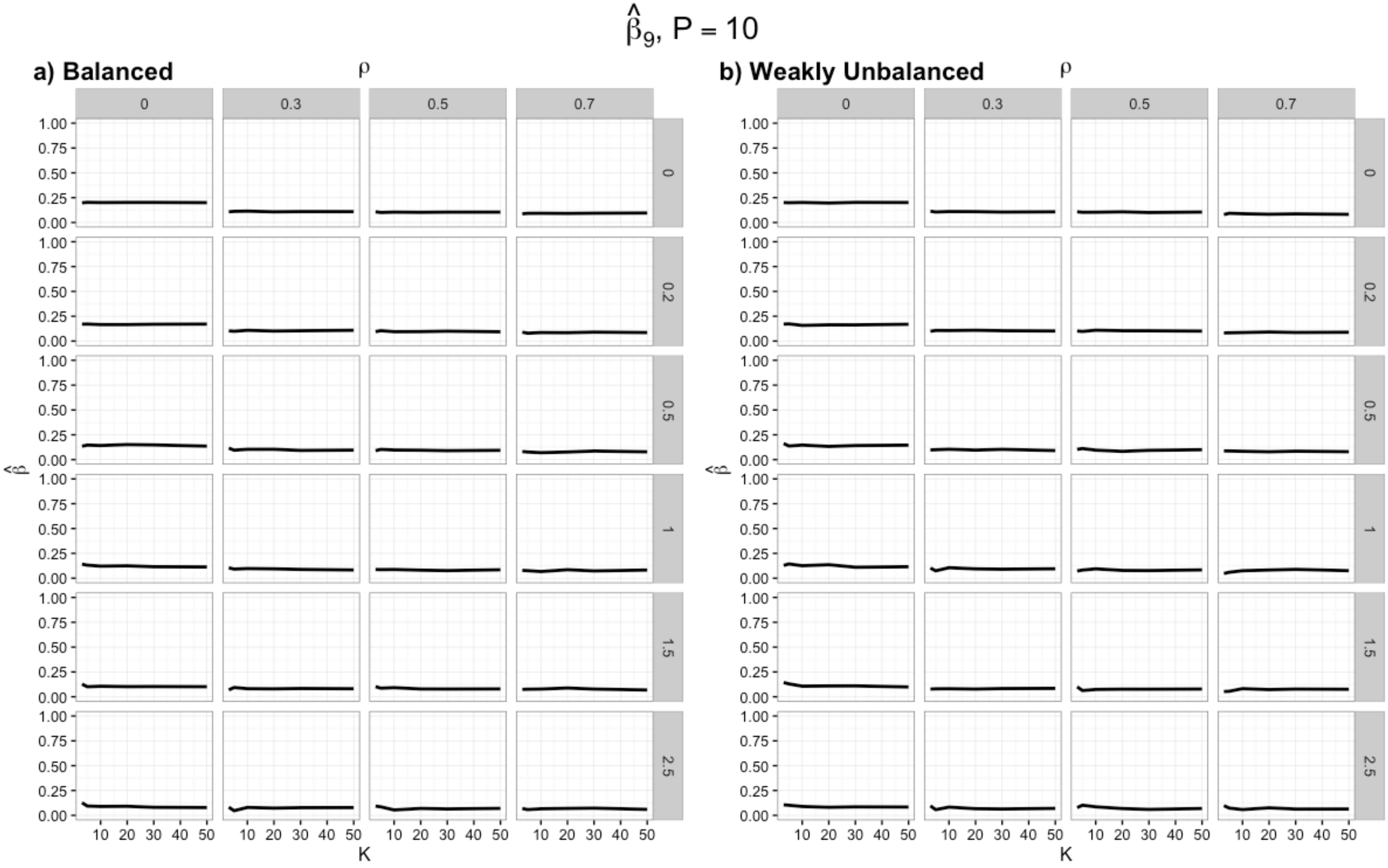
$\hat{\beta}_9$, P = 10
a) Balanced
b) Weakly Unbalanced
ρ
0
0.3
0.5
0.7
0
0.2
0.5
1
1.5
2.5
1.00
0.75
0.50
0.25
0.00
$\hat{\beta}$
10 20 30 40 50
K

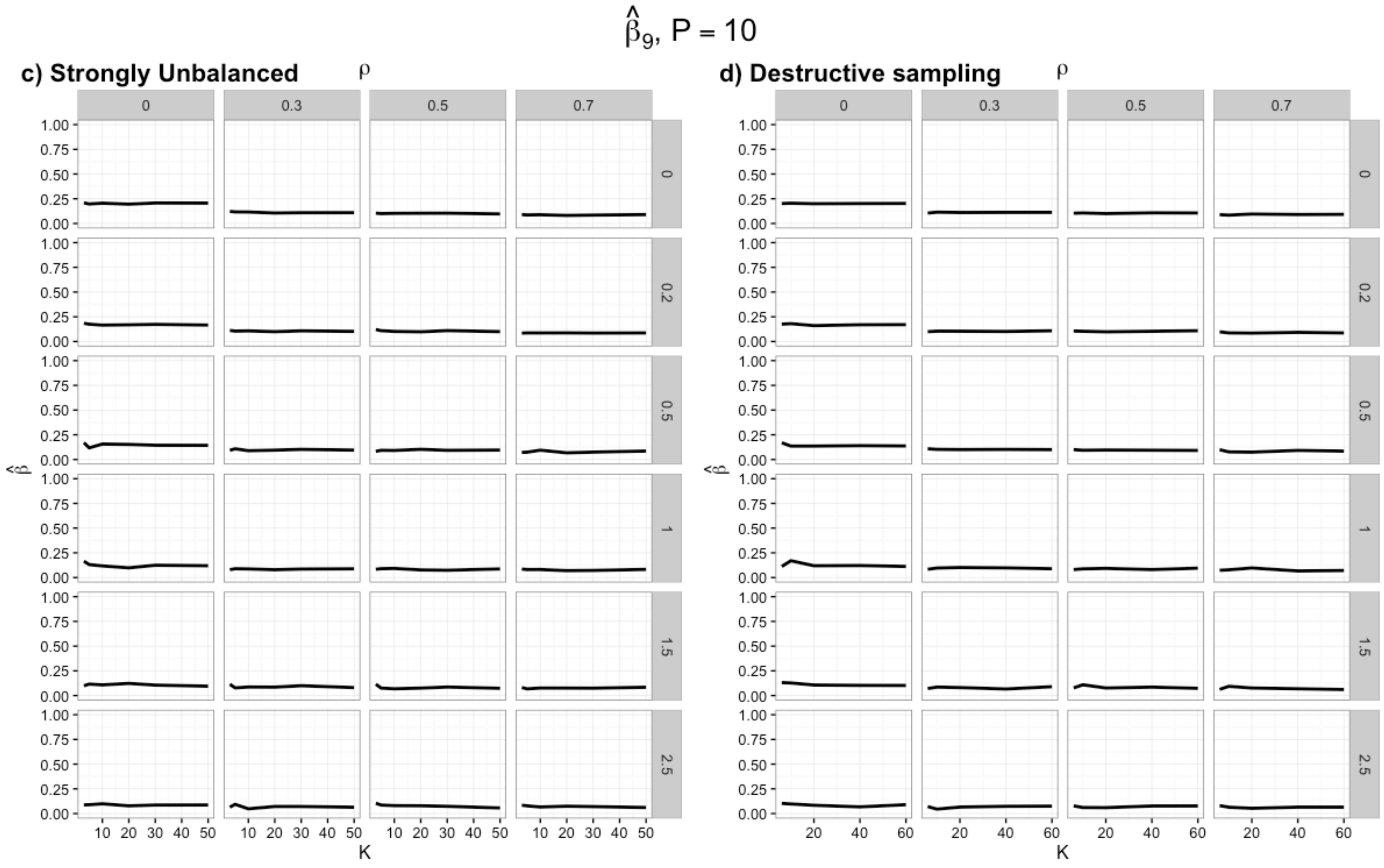
$\hat{\beta}_9$, P = 10
c) Strongly Unbalanced
d) Destructive sampling
ρ
0
0.3
0.5
0.7
0.2
1
1.5
2.5
0.00
0.25
0.50
0.75
1.00
$\hat{\beta}$
10
20
30
40
50
60
K

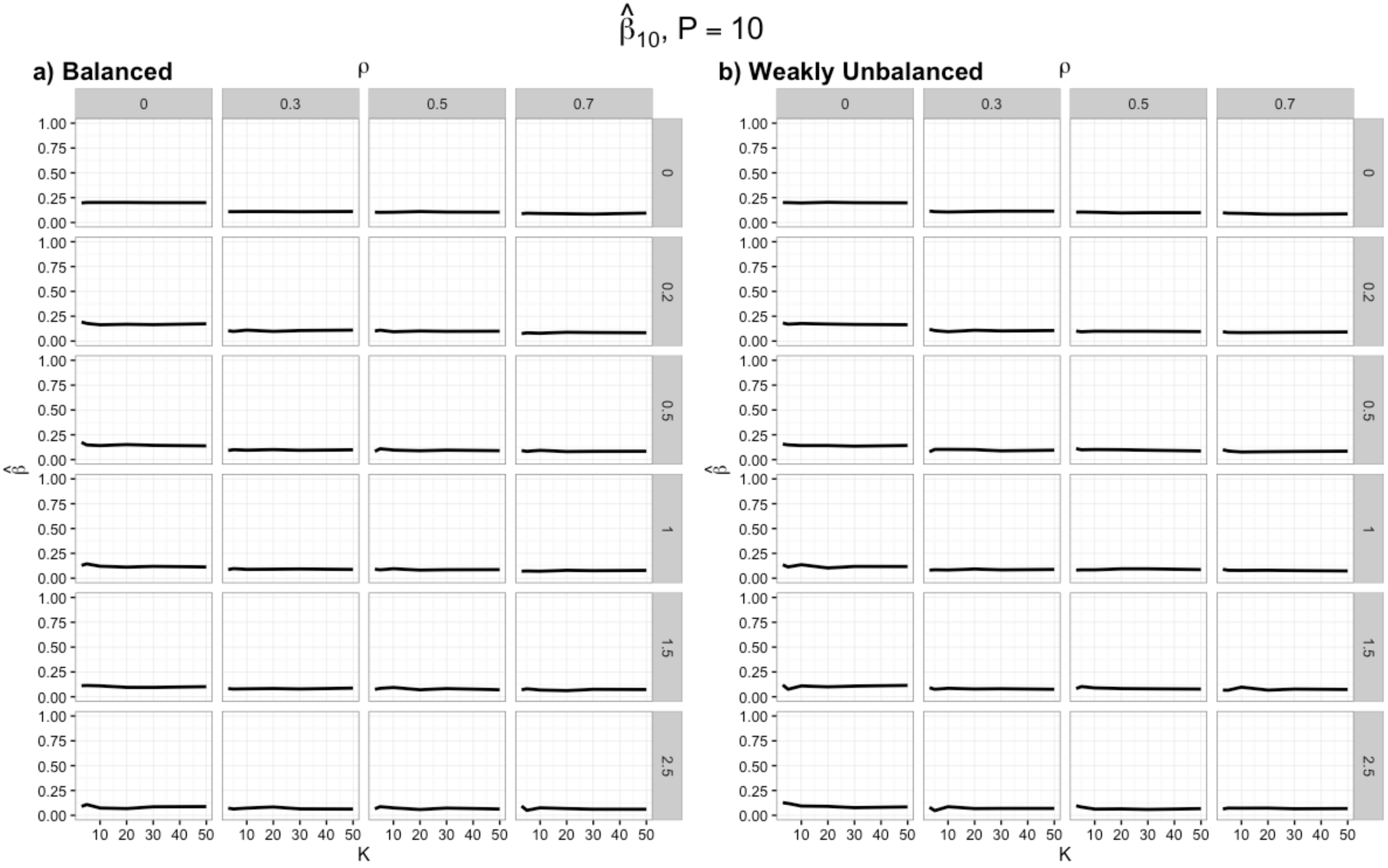

$\hat{\beta}_{10}$, P = 10
a) Balanced
b) Weakly Unbalanced
ρ
0
0.3
0.5
0.7
0
0.2
0.5
1
1.5
2.5
1.00
0.75
0.50
0.25
0.00
$\hat{\beta}$
10 20 30 40 50
K

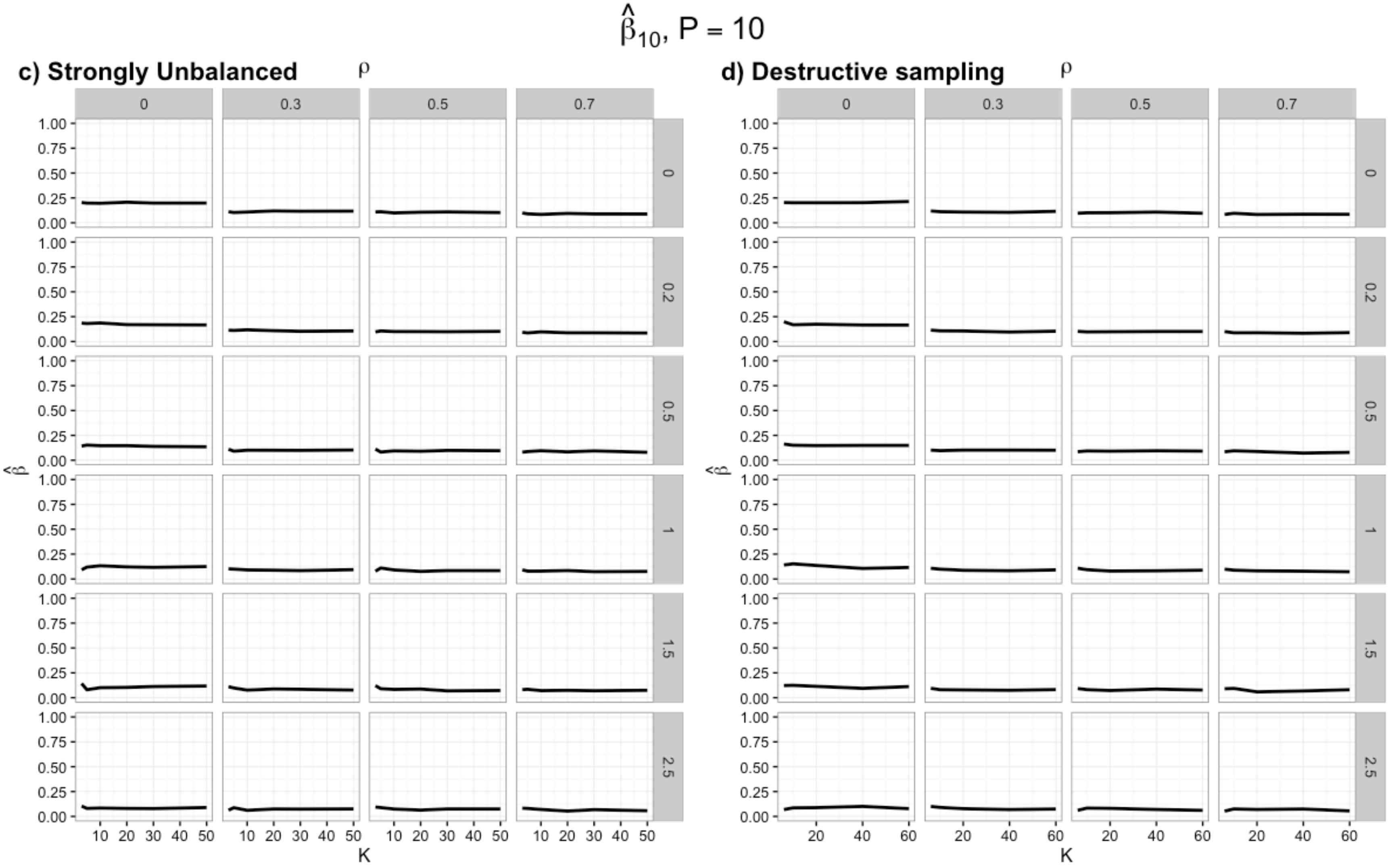

$\hat{\beta}_{10}$, P = 10
c) Strongly Unbalanced
d) Destructive sampling
ρ
0
0.3
0.5
0.7
0
0.2
0.5
1
1.5
2.5
1.00
0.75
0.50
0.25
0.00
$\hat{\beta}$
10
20
30
40
50
60
K
